# Morphological Characterization and DNA Barcoding of Duckweed Species in Saudi Arabia

**DOI:** 10.3390/plants10112438

**Published:** 2021-11-12

**Authors:** Mohammed Al-Dakhil, Salem Alghamdi, Hussein Migdadi, Muhammad Afzal, Ahmed Abdelrahim Ali

**Affiliations:** 1 Natural Resources and Environmental Research Institute, King Abdulaziz City for Science and Technology, Riyadh 11442, Saudi Arabia; 2 Department of Plant Production, College of Food and Agriculture Sciences, King Saud University, Riyadh 11451, Saudi Arabia; salem@ksu.edu.sa (S.A.); mmushtaq@ksu.edu.sa (M.A.); arahim.eng@gmail.com (A.A.A.); 3 National Agricultural Research Center, Baqa, Amman 19381, Jordan

**Keywords:** duckweeds, identification, morphological, DNA barcoding, phylogenetic analysis

## Abstract

Duckweeds, or Lemnaceae, are widespread aquatic plants. Morphology-based identification of duckweed species is difficult because of their structural complexity. Hence, molecular tools provide significant advantages for characterizing and selecting species or clones for sustainable commercial use. In this study, we collected and characterized ten duckweed isolates from nine different regions in Saudi Arabia (SA). Based on the morphological characterization and phylogenetic analysis of intergenic spacer sequences of chloroplast DNA using six barcoding markers, the clones were classified into three genera, represented by seven species: *Lemna gibba* L., *Lemna minor* L., *Lemna japonica* Landolt, *Lemna aequinoctialis* Welw., *Lemna perpusilla* Torr., *Spirodela polyryiza* (L.) Schleid., and *Landoltia punctate* G. Mey. *Lemna gibba* was revealed to be a distinct dominant duckweed species in many regions of SA. Five barcoding markers showed that *L. gibba*, *L. minor*, and *L. punctata* were the most widely distributed species in the country. However, *L. punctata, L. perpusilla*, and *S. polyryiza* were the dominant species in the Al-Qassim, Madinah-1, and Madinah-2 regions, respectively. Moreover, the morphological traits revealed variations for these clones, relative to other studied duckweed clones. According to the results obtained in this study, three out of six plastid markers (*trn*H-*psb*A, *matK*, and *atp*F-*atp*H) helped to identify the dominant duckweed species in Saudi Arabia. Further evaluation based on adaptability, molecular genetic studies, and functional genomics is needed for these species to be used at the commercial level in Saudi Arabia.

## 1. Introduction

Under the arid conditions of Saudi Arabia, the agricultural sector plays a significant role in food security for humans and animals, as well as economic, social, and sustainable development. Saudi Arabia, with a Vision 2030 strategic plan, aims to enact sustainable development in food production by saving freshwater resources, the environment, and energy. The government supports research on new feed resources for livestock and poultry that maintain high protein value and high water and fertilizer use efficiency. Duckweed is an aquatic plant that contains crude protein up to 45% of its dry weight under optimum conditions and may be used, without further processing, as a complete feed for animals and fish [1]. Duckweed starch content has been reported to be from 5% to 70% of its dry weight. The high level of starch and low lignin and cellulose levels make duckweed a promising feedstock for biofuel production [2]. Duckweed plants float on the surface or are slightly submerged in water. Leaf-like fronds are modified leaves and stem that mainly function in photosynthesis and vegetative reproduction. As a result, the rate of biomass production is high, providing the basis for various practical applications of duckweed in food and feed sustainability [3]. For commercial utilization of duckweed, knowledge of its taxonomy is required. At the morphological level, delineating duckweed species has been shown to be difficult. Many studies have tried to decrease the anatomical complexity based on the chemical composition of flavonoids [4] and to delineate duckweed species using allozyme, which has helped advance morphology techniques [5,6]. The genome size of duckweed varies 13-fold, ranging from 150 Mb in *Spirodela polyrhiza* (L.), Schleid. to 1881 Mb in *Wolffia arrhiza* Wimm. With the development of sequencing technology and bioinformatics, five duckweed genomes from *Spirodela* and *Lemna* have been sequenced and assembled [7]. Thirty-eight duckweed species have been identified and characterized [8]. With molecular biology tools and techniques (polymerase chain reaction and Q-PCR, DNA barcoding, and high-throughput DNA sequencing tools), a taxonomic analysis at the molecular level offers a more profound understanding than chemotaxonomy. Therefore, among the various DNA analysis methods for taxonomic definition, molecular taxonomy is considered the most suitable method for identifying and evaluating the genetic potential of duckweed species [9]. There are only a few studies in the literature on identifying duckweed genera and species in Saudi Arabia. Masrahi et al. [10] reported the first record of *Wolffiella hyalina* (Delile) Monod in Saudi Arabia and regarded it as an addition to the flora of Saudi Arabia. The present study aimed to assess the genetic variability based on morphological traits and molecular (DNA barcoding) markers among duckweed clones collected from different ecosystems.

## 2. Results

**Figure 1:**
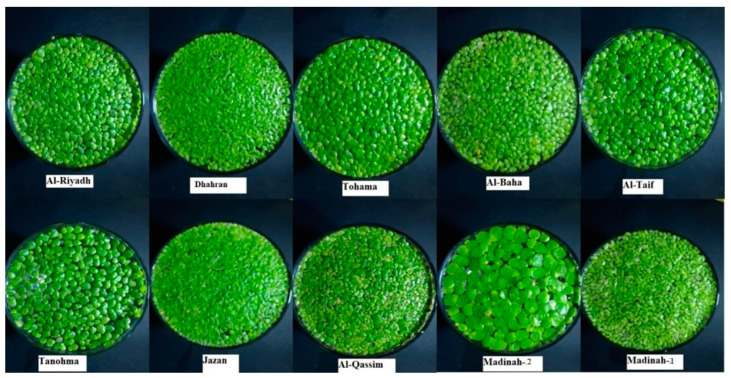
Duckweed clones collected from Saudi Arabia were used for morphological characterization and molecular identification.

The identification of duckweed clones, collected from ten locations in Saudi Arabia, was made through morphological and molecular characterization (Figure 1). Morphological variation was recorded based on the different measurements (Table 1 and Table 2). All clones showed obovate fronds shape and rounded frond apex, and the turions were present. The frond shape of the clones from the Riyadh, Taif, Tohama, Tanomah, Qassim, and Madinah-1 regions was asymmetric, while those from the remaining regions were symmetric. The frond color of clones from five regions (Riyadh, Tohama, Al-Baha, Qassim, and Madinah-1) were dark green, and, in five regions (Taif, Jazan, Tanomah, Dhahran, and Madinah-2), the frond color was recorded as light green. All the studied duckweed clones showed frond apexes and turions. The analysis of variance for frond and root traits showed highly significant differences among the clones (Table 2). The number of roots ranged from 1.67 to 9.22; moreover, the Madinah-2 and Al-Qassim clones had the highest number of roots and differed significantly from other clones. The maximum number of roots, i.e., 9.22, was recorded for the Madinah-2 clones, while the Al-Baha clones had the lowest number of roots, i.e., 1.67. The Tanomah clones had the longest roots (3.76 mm), and the Madinah-2 clones had the shortest roots (0.74 mm). Regarding the characteristics of fronds, clones collected from the Al-Qassim region had the highest number of fronds (6.0), while clones from the Riyadh region had the longest fronds (7 mm). The widest and thickest clones were 8.5 mm from the Madinah-1 region and 3.2 mm from the Al-Taif region, respectively.

**Figure 2:**
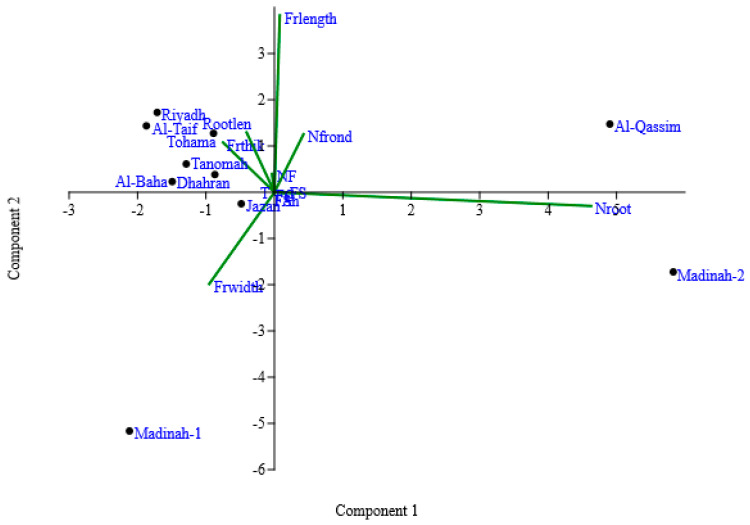
Two-dimensional biplot ordination of the duckweed clones on principal component axes according to qualitative and rescaled quantitative data traits. Principal component 1 (PC1) represented 46.4% of the total variance, and PC2 represented 24.3% of the total variance. Frond shape (FSh); frond symmetry (FS); frond color (FC); frond apex (FA); turions (T); the number of roots—Nroot; frond width—Frwidth; frond thickness (Frthick); frond length (Frlength); the number of contiguous fronds (NCF); root length (Rootlen); the number of fronds (Nfrond).

To facilitate interpretation of the correlation matrix, produced from the quantitative and qualitative morphological traits for ten duckweed clones, a principal component analysis (PCA) was applied. The PCA plots provided a visual overview of how different traits influenced the distribution of the duckweed clones. The first three principal components explained 88.27% of the total variance (i.e., PC1 46.4%, PC2 24.3%, and PC3 17.51%). The plot for the PC1 and PC2 showed that some traits described the same variation among the clones (Figure 2). The number of roots formed the underlying dimension for PC1, with a value of 0.95 positive loadings; whereas, frond width (−0.2) was negatively loaded and placed on the left-hand side of the plot. PC2 explained 24.3% of the variance and showed a positive correlation with frond traits, such as frond length (0.79), number of fronds (0.26), and frond thickness (0.22), besides root length (0.26). PC3 was loaded with frond width with positive loading (0.76). The Al-Qassim and Madina-2 clones showed the most positive loading in PC1, with 4.9 and 5.8, respectively, while Madina-1 clones had the most negative loading on PC1 and PC2. Those clones were considered to be the most divergent clones.

The results generated from the six plastid markers (*psb*K-*psb*I, *trn*H-*psb*A, *atp*F-*atp*H, *mat*K, *rpo*C1, and *rbc*L) for species identification, and their accessions numbers, are presented in Table 3, and the sequences generated from each sample are shown in Supplementary Table S1. The clones collected from the Riyadh, Dhahran, Tohama, Al-Baha, Al-Taif, and Tanomah regions represented the same species (Table 3). However, the duckweed clones collected from the Jazan region represented *L. aequinoctialis*, and all the sequence data markers confirmed its exact identification, except that *rbc*L identified *L. minor*. Similarly, clones collected from the Al-Qassim and Madinah (Madinah-1 and Madinah-2) regions were identified as *L. punctata*, *L. perpusilla*, and *S. polyryiza*, respectively.

**Figure 3:**
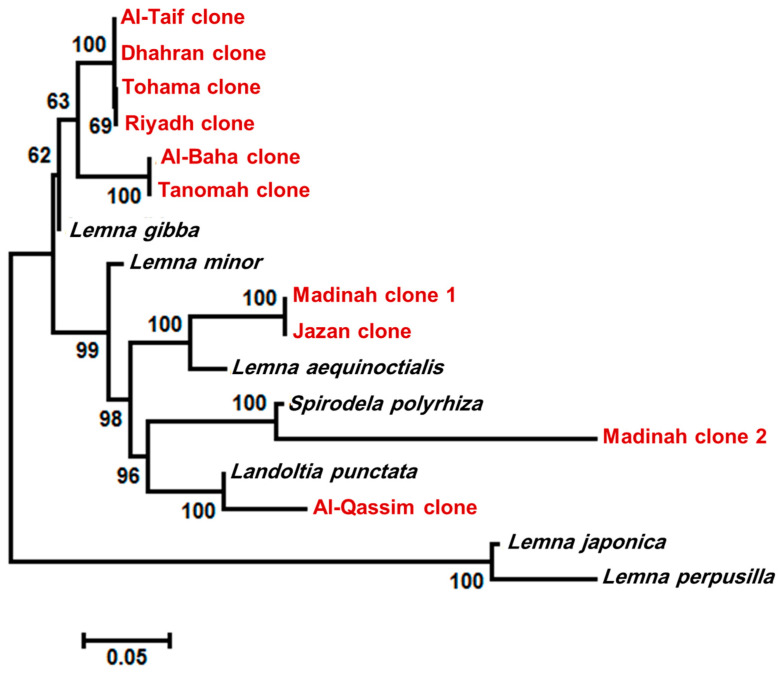
Phylogenetic tree based on maximum likelihood methods with 1000 bootstraps constructed in MEGA 10.0 using the concatenated sequences of *psb*K-*psb*I, *trn*H-*psb*A, and *atp*F-*atp*H intergenic spacer region, and *mat*K, *rpo*C1, and *rbc*L gene. The numbers on the nodes represent the percentage of bootstrap values from 1000 replicates. The genetic distances are indicated by the horizontal bar.

After sequencing, the data were cleaned and aligned using the Bioedit software, and the species-level phylogenetic tree was constructed. According to the six markers, the duckweed clones, isolated from the Riyadh region, were identified as *L. gibba* by four markers (*trn*H-*psb*A, *mat* K, *atp*F-*atp*H, and *rbc*L), while *L. japonica* was recognized by *trn*H-*psb*A, and *L. minor* was recognized by *rpo*C1. Similar results were recorded for the Dhahran, Tohama, Al-Baha, Al-Taif, and Tanomah regions. Most markers identified the Jazan isolate as *L. aequinoctialis*, but it was identified as *L. minor* only by the *rbcL* marker. However, the duckweed clones isolated from Al-Qassim and Madinah-2 were identified as *L. punctata* and *S. polyryiza* by all markers, respectively. The clones from Madinah-1 were identified as *L. perpusilla* with three markers (*psb*K-*psb*I, *trn*H-*psb*A, and *mat*K). In comparison, two markers (*atp*F-*atp*H and *rpo*C1) identified the isolate as *L. aequinoctialis*, and *rbc*L identified it as *L. minor*. Based on multiple nucleotide sequence alignment (Figure S1), divergence score matrix (Figure S2), and the phylogenetic tree (Figure 3), four species were mostly distributed in Saudi Arabia, which were *L. gibba*, *L. aequinoctialis*, *L. punctata*, and *S. polyryiza* (Figure 3).

## 3. Discussion

Out of seven different haplotypes identified, four were dominant in the ten regions, identified using DNA barcoding and phylogenetic analysis. The frond shape, frond apex, and turions registered the same obovate, rounded, and present, respectively, for all duckweeds clones. In this study, the number of roots and frond length significantly contributed to the genetic variability among collected clones, confirming that they were the most influential variability components. A similar morphological identification was made earlier by [11] using morphological characterization for species identification among duckweed. This grouping included *S. polyrhiza*, *L. aequinoctialis*, *L. punctata*, and *W. globose*. It was also suggested that the low degree of differentiation among members of the Lemnaceae family most likely owes to adaptation to the highly specialized way of aquatic life [12].

Similar results were reported by Azer [13], who classified the *Lemna* genus into three different clusters, based on morphological characterization. The first cluster included *L. gibba* and *L. minor*, while the second and third clusters included *L. trisulca* and *L. aequinoctialis*. Bog et al. [14] classified the *Lemna* genus into four sections. It was also suggested that the similarities in specific morphological characteristics were closely connected to *L. gibba* and *L. minor*. A molecular study based on DNA barcoding results also recorded *L. japonica*, *L. gibba*, and *L. minor* aggregated in the same group. However, the phylogenetic analysis confirmed this relationship and reported that *L. japonica*, *L. gibba*, and *L. minor* were present in the same group.

We can infer that the samples collected from the Riyadh, Dhahran, Tohama, Al-Baha, Al-Taif, and Tanomah regions had similar traits (morphological characterization). The traits include long roots, having many fronds with veins that inflate, and few seeds ribbed with *L. minor* and fewer with *L. gibba*, but not in *L. aequinoctialis* and *L. trisulca* [14]. Our study recorded similar results—i.e., samples collected from the Qassim, Madinah-1, and Madinah-2 regions belonged to three different genera and recorded unique characteristics. relative to other duckweed strains. In contrast, a detailed key is available for identifying species based on morphological traits [11]. A lack of experience and expertise in morphological taxonomy limits using this key to identify duckweed species worldwide. We also used six different plastid markers to identify duckweed species in Saudi Arabia because of limited morphological markers.

Similarly, Klaus et al. [15] suggested that plastid spacer barcoding markers have shown to be reliable tools for identifying duckweed species, especially when used in conjunction with several markers. However, all clones of the same species cannot be differentiated by these markers alone. In this context, several other plastid and mitochondrial markers are good options for obtaining excellent results [8,16], or another global marker (i.e., AFLP) could serve the purpose [14]. Zhang and Azizullah [17] stated that, although duckweeds can occasionally be found in single or more complex communities, they often exist in two or three species communities. Using ISSR markers to assess the genetic variability in populations of seven species in genus *Lemna* and *Spirodela* from China and Vietnam, Xue et al. [18] reported genetic differentiation of duckweeds, suggesting that geographic differentiation had a significant influence on the genetic diversity of duckweeds. In a previous report, duckweed clones from tropical Africa showed higher genetic diversity than those from other regions, probably because of higher temperatures and radiation in the equatorial region [19].

A comprehensive phylogenetic analysis has also been carried out using the morphological, anatomical characters, flavonoids, and molecular analysis (chloroplast and introns) sequences were used to classify the Lemnaceae species and phylogenetic analysis confirms the *lemna*, *landoltia*, *spirodela*, *wolffian*, and *wolffiella* in the grouping [20]. The current study also identifies similar results, i.e., *L. gibba*, *L. minor*, *L. japonica*, and *L. aequinoctialis* using the morphological and molecular combined data. Wang et al. [8] reported delineating the genus *Lemna*, based on several plastid sequences. Tippery et al. [21] also investigated the use of various plastid and nuclear markers (ETS and ITS), and Borisjuk et al. [22] used *psb*K-*psb*I and *atp*F-*atp*H. Similar results were recorded during our study, *L. gibba*, *L. minor*, and *L. japonica* (Riyadh, Dhahran, Tohama, and Al-Baha), while other clones were recorded in different genera (*L. perpusilla*/*L. aequinoctialis*, *L. punctata*, *S. polyryiza*) using the plastid markers. Landolt [23] assigned the highest morphological primitiveness to *L. gibba*, while the lowest was assigned to *L. minuta* and *L. valdiviana*, which possibly signifies the genus *Lemna* as the basal species, while the latter represents the most derived species. In several projects, most *Lemna* species could be easily identified using DNA barcoding.

Xu et al. [24] suggested that duckweeds were the only occasionally observed aquatic plants present in large reservoirs and natural rivers. Our observations show that *L. aequinoctialis* is the best candidate for future regeneration applications in the Jazan region, where samples are collected from rain pools (a small area of still water, typically one formed naturally), usually receive heavy rain during the year. These pools are distributed on top of mountains and are, perhaps, the best among the pool. The genus *Lemna* has been studied for several years. However, the taxonomic identification boundaries are not yet adequately resolved, and there are still several botanists who disagree with the identification of *Lemna* species. Most of its features are changed and significantly overlap. Because of the variability of this genus, taxonomists have had controversy for over a century and treated the *Lemna* genus into several species [14,25]. It is known that *S. punctata, S. intermedia*, and *S. polyrhiza* represent the same genus, called *Spirodela* [23]. According to morphological traits, *S. intermedia* is the most primitive *Lemnaceae* species, and the highest primitiveness is found in this species [9]. By applying molecular tools for taxonomy to the *Lemnaceae* family, these three species were separated into two different genera, i.e., *Spirodela* and *Landoltia* (changing the nomenclature of the species *S. punctata* to *L. punctata*), after the analysis of the plastid *rbc*L sequence [26]. The next generation sequencing technique may be considered, since it is less expensive; additionally, the entire chloroplast genome opens a new avenue to resolve species identification issues. Recent advances in organelles [8] and sequencing of nuclear genomes [27] could mine potential markers further.

According to the *rps*16 data of [16], the *W. globosa* grouping is composed of the six genera of the *W. globosa* species. While the plastid markers *rp16* and *atp*F-*atp*H showed genetic differences in *L. aequinoctialis* and *W. globosa* recorded relatively low diversity in a cosmopolitan environment [28], it is questionable if a longer sequence of clones would significantly increase this genetic diversity. While the genetic background of these clones was also widely distributed, *S. polyrhiza* was dispersed. The geographical influence on genetic diversity appears to be higher, as variation was found among all duckweed clones in the Jazan south region compared with clones in the northern regions, especially in the *L. aequinoctialis* species [24]. Our results recorded that the exact geographical positions (Qassim (*L. punctata*), Madinah-1 (*L. perpusilla*), Madinah-2 (*S. polyryiza*)), met the geographic environment. The lower latitude and higher temperatures, and ultraviolet radiation to the south of the Hainan Island could be responsible for this similarity [29]. The presence of *L. punctata*, *L. perpusilla*, and *S. polyryiza* could also contribute to their widespread distribution and adaptability to different environmental conditions in the region.

## 4. Materials and Methods

### 4.1. Plant Material

**Figure 4:**
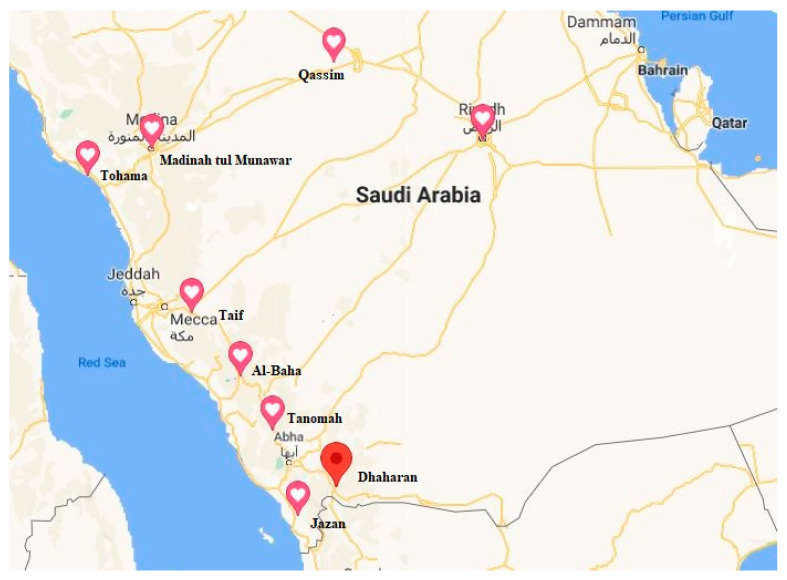
Saudi duckweed collection sites. The latitude (N), longitude (E), and altitude in meters of the locations are as follows: Riyadh→24°39′31″→46° 36′26″→614; Dhahran→17°40′36″→43°20′53″→1717; Tohama→19°48′52.4″→41°37′14.6″→2070; Al-Baha→20°00′46.3″→41°29′12″→2103; Jazan→17°01′56″→43°09′04″→947; Al-Taif→21°12′45″→40°27′17″→1672; Tanomah→18°54′37.2″→42°12′29.4″→2228; Al-Qassim→26°24′22″→44°01′04″→594; Madinah-1→24°37′38″→39°54′08″→821; Madinah-2→24°37′38″→39°54′08″→821.

The duckweed clones analyzed in this study were collected from ten different regions of Saudi Arabia (Figure 4). The plants were rinsed in clean water, cultured in half-strength Hoagland solution, and acclimated for one week before starting the experiments. A single plant from each clone was cultured at 28 °C, in 14 h of light per day, for two weeks for multiplication (Figure 1). The study was conducted at the greenhouse of the College of Food and Agriculture Sciences, King Saud University, Riyadh, Saudi Arabia.

### 4.2. Morphological Characteristics

The duckweed clones were transferred to a polypropylene container (15 × 10.5 × 7.5 cm^3^) filled with 750 mL of half-strength Hoagland solution. Three replicates were represented in each clone. Two weeks after establishment, from each replicate, ten plants for each clone were harvested. The following data were recorded, according to Ceschin et al. [30] as follows: qualitative characteristics (such as frond shape, symmetry, color, apex, and turions), while quantitative characteristics (frond length (mm), width (mm), thickness of aerenchyma layers, number of contiguous fronds (n), length, width, number of roots, and root length) were used in the evaluation.

All data was subjected to the analysis of variance (ANOVA) for the quantitative morphological traits of the studied duckweed clones. Mean and standard deviation of the evaluated traits (*n* = 10 samples and three replications, total = 30). Mean separation was based on Tukey HSD at the 0.05 probability level.

### 4.3. Molecular Markers Analysis: DNA Barcoding 

Genomic DNA was extracted from a single plant from each replicated clones after grinding duckweed fronds in liquid nitrogen using a DNA extraction kit (Qiagen, Hilden, Germany), following the company’s instructions. For DNA barcoding, six plastid markers (psbK-psbI, trnH-psbA, atpF-atpH, matK, rpoC1, and rbcL) were used for DNA barcoding and identified different clones of duckweeds. The markers were selected based on duckweed reference sequences, as proposed by the plant working group of the Consortium for the Barcode of Life (CBOL), including three non-coding spacers (psbK-psbI, trnH-psbA, and atpF-atpH) and four plastid coding genes (matK, rpoB, rpoC1, and rbcL) were used to identify and characterize duckweed clones [31,32,33]. The amplicon sizes were estimated according to Wang et al. [8]. The PCR reaction comprised 50 ng genomic DNA, five pmol of each marker, 1X PCR Master Mix ((Promega, Fitchburg, WI, USA), and the volume completed to 25 µL. The PCR reaction was set as follows: initial denaturation at 94 °C for 5 min, denaturation at 94 °C for 1 min, with the annealing temperature set for each marker, and extension at 72 °C for 1 min for 35 cycles. However, the final extension time was 72 °C for 5 min. The PCR products were purified using Wizard SV gel and a PCR clean-up system (Promega, Fitchburg, WI, USA). The purified PCR samples were sent to Macrogen, South Korea (https://www.macrogen.com/en/main/index.php) (accessed on 6 December 2020) for sequencing by ABI3730 automated sequencer using the same markers as in the PCR reactions. The strands were both sequenced and checked for ambiguous nucleotides. 30 bp at the ends of each read were removed, the length of amplicon products was measured, and multiple DNA sequence alignments were generated using ClustalW alignment software [34,35]. The phylogenetic tree was constructed using MEGA X to group the clones with following matrix: DNA weight matrix = IUB, transition weight = 0.50, delay divergent cutoff 30% [36].

### 4.4. Phylogenetic Analysis Based on DNA Sequence

The evolutionary history was inferred by using the maximum likelihood method and the Tamura–Nei model [36]. The optimal tree with the sum of branch length = 10.41207259 is shown. The evolutionary distances were computed using the maximum composite likelihood method and are in the units of the number of base substitutions per site. This analysis involved 82 nucleotide sequences with the following Genbank accession numbers: OK546023, OK571365, OK350360, OK350359, OK546024, OK350358, OK350361, OK350363, OK350362, OK546025, KJ921758, KJ136047, GU454305, GU454300, MG000445, GU454502, GU454494, GU454491, OK103562, OK383787, OK383788, OK103563, OK383786, OK383789, OK383790, OK247674, OK571366, OK247675, OK095301, OK375248, OK546020, OK571364, AY034197.1, OK375249, OK546021, OK546026, OK375250, KX526519, OK546022, KP017666.1, OK376426, Ok546019, OK376427, OK493446, KF726306.1, OK493447, KP017715.1, OK493448, OK493449, OK493450, KP017720, OK493451, OK493452, KP017721.1, OK095300, OK383787, OK598959, KX212889, OK598958, OK598957, OK598956, KJ630548, OK598955, KJ630555, OK598954, OK247676, KJ630513.1, OK571367, OK598946, OK598947, OK598949, JN114815.1, OK598948, GQ436374, OK598951, OK598952, OK571368, AY034223, OK571369, OK598950, and KC584885. All ambiguous positions were removed for each sequence pair (pairwise deletion option). There were a total of 1611 positions in the final dataset. Evolutionary analyses were conducted in MEGA X [37] using Matrix Representation with Parsimony method [38]. The numbers on the nodes represent the percentage of bootstrap values from the 1000 replicates.

## 5. Conclusions

This study characterized duckweed clones collected from ten different Saudi Arabia regions at both morphological and molecular levels. The results should help to inform research on the indigenous duckweed clones adapted to the climate of Saudi Arabia. Three out of six plastid markers (*trn*H-*psb*A, *matK*, and *atp*F-*atp*H) helped to identify the dominant duckweed species in Saudi Arabia. Evaluation of performing *L. punctata*, *L. perpusilla*, and *S. polyryiza* clones in environments such as in Saudi Arabia is needed for future uses such as animal feed supplies and bioremediation programs.

## Figures and Tables

**Table 1:** Qualitative morphological traits for studied duckweed clones of the ten locations of Saudi Arabia (*n* = 10 samples from each clone averaged and replicated three times, total samples = 30 reads). Frond shape—FSh; frond symmetry—FS; frond color—FC; frond apex—FA; turions—T.

	FSh	FS	FC	FA	T
Riyadh	Obovate	Asymmetry	Dark Green	Rounded	Present
Dhahran	Obovate	Symmetry	Light Green	Rounded	Present
Tohama	Obovate	Asymmetry	Dark Green	Rounded	Present
Al-Baha	Obovate	Symmetry	Dark Green	Rounded	Present
Al-Taif	Obovate	Asymmetry	Light Green	Rounded	Present
Tanomah	Obovate	Asymmetry	Light Green	Rounded	Present
Jazan	Obovate	Symmetry	Light Green	Rounded	Present
Al-Qassim	Obovate	Asymmetry	Dark Green	Rounded	Present
Madinah-1	Obovate	symmetry	Light Green	Rounded	Present
Madinah-2	Obovate	Asymmetry	Dark Green	Rounded	Present

**Table 2:** Analysis of variance (ANOVA) for the quantitative morphological traits of the studied duckweed clones. Mean and standard deviation of the evaluated traits (*n* = 10 samples and three replications, total = 30).

	Number of Roots	Frond Width (mm)	Frond Thickness (mm)	Frond Length (mm)	NCF Number of Contiguous Fronds	Root Length (mm)	Number of Fronds
Riyadh	2.00 ± 0.01 b	5.7 ± 0.12 b	2.3 ± 0.17 c	7.0 ± 0.02 a	4.0 ± 0.01 b	2.35 ± 0.13 b	4.00 ± 0.01 b
Al-Taif	2.00 ± 0.01 b	5.8 ± 0.06 b	3.2 ± 0.06 a	6.5 ± 0.06 b	5.0 ± 0.01 a	2.02 ± 0.32 b	3.67 ± 0.50 b
Tohama	3.00 ± 0.01 b	5.8 ± 0.06 b	3.0 ± 0.00 b	6.5 ± 0.04 b	3.0 ± 0.01 c	2.29 ± 0.37 b	4.00 ± 0.01 b
Jazan	2.44 ± 0.53 b	3.5 ± 0.10 d	0.5 ± 0.06 f	4.6 ± 0.04 e	3.0 ± 0.01 c	0.84 ± 0.05 c	3.67 ± 0.50 b
Tanomah	1.89 ± 0.33 b	3.4 ± 0.06 d	0.5 ± 0.0 f	4.5 ± 0.09 e	4.0 ± 0.01 b	3.76 ± 0.49 a	3.56 ± 0.53 b
Al-Baha	1.67 ± 0.50 b	3.5 ± 0.06 d	1.0 ± 0.0 d	4.7 ± 0.09 e	4.0 ± 0.01 b	1.98 ± 0.45 b	3.00 ± 0.01 bc
Dhahran	2.00 ± 0.01 b	3.0 ± 0.01 e	0.4 ± 0.0 f	5.0 ± 0.07 d	3.0 ± 0.01 c	1.54 ± 0.28 b	3.22 ± 0.44 bc
Al-Qassim	8.11 ± 0.78 a	4.0 ± 0.01 c	0.7 ± 0.0 e	6.0 ± 0.06 c	5.0 ± 0.01 a	2.14 ± 0.09 b	6.00 ± 0.01 a
Madinah-1	2.00 ± 0.01 b	8.5 ± 0.01 a	0.9 ± 0.0 d	1.0 ± 0.06 g	4.0 ± 0.01 b	0.84 ± 0.09 c	2.89 ± 0.33 bc
Madinah-2	9.22 ± 2.39 a	4.0 ± 0.06 c	0.4 ± 0.0 f	4.0 ± 0.05 f	3.0 ± 0.01 c	0.74 ± 0.09 c	2.44 ± 0.53 bc
Tukey HSD	1.60	0.38	0.18	0.45	0.42	0.60	1.10

Means followed with the same letter in the column were not significantly different based on Tukey HSD, *p* < 0.05.

**Table 3:** DNA barcoding-based identifications and accessions number among 10 duckweeds clones.

Region	*psb*K-*psb*I	*trn*H-*psb*A	*mat*K	*atp*F-*atp*H	*rpo*C1	*rbc*L
Riyadh	*L. japonica* (OK546023)	*L. gibba* (OK103562)	*L. gibba* (OK095301)	*L. gibba* (OK095300)	*L. minor* (OK376426)	*L. gibba* (OK571367)
Dhahran	*L. japonica* (OK571365)	*L. gibba* (OK383787)	*L. gibba* (OK375248)	*L.gibba* (OK598953)	*L. gibba* (Ok546019)	*L. gibba* (OK598946)
Tohama	*L. japonica* (OK350360)	*L. gibba* (OK383788)	*L. gibba* (OK546020)	*L. gibba* (OK598959)	*L. minor* (OK376427)	*L. gibba* (OK598947)
Al-Baha	*L. japonica* (OK350359)	*L. gibba* (OK103563)	*L. gibba* (OK571364)	-----	*L. minor* (OK493446)	*L. gibba* (OK598949)
Jazan	*L. perpusilla* (OK350358)	*L. aequinoctialis* (OK383786)	*L. aequinoctialis* (OK375249)	*L. aequinoctialis* (OK598956)	*L. aequinoctialis* (OK493447)	*L. minor* (OK598948)
Al-Taif	*L. japonica* (OK546024)	*L. gibba* (OK383789)	*L. gibba* (OK546021)	*L. gibba* (OK598958)	*L. gibba* (OK493448)	*L. gibba* (OK598951)
Tanomah	*L. japonica* (OK350361)	*L. gibba* (OK383790)	*L. gibba* (OK546026)	*L. gibba* (OK598957)	*L. minor* (OK493449)	*L. gibba* (OK598952)
Al-Qassim	*L. punctata* (OK350363)	*L. punctata* (OK247674)	*L. punctata* (OK375250)	*L. punctata* (OK598955)	*L. punctata* (OK493450)	*L. punctata* (OK571368)
Madinah-1	*L. perpusilla* (OK350362)	*L.aequinoctialis* (OK571366)	*L.aequinoctialis* (OK546022)	*L. aequinoctialis* (OK598954)	*L. aequinoctialis* (OK493451)	*L. minor* (OK571369)
Madinah-2	*S. polyryiza* (OK546025)	*S. polyryiza* (OK247675)	-----	*S. polyryiza* (OK247676)	*S. polyryiza* (OK493452)	*S. polyryiza* (OK598950)

## Data Availability

All the data supporting this article were included in the main text.

## Supplementary Materials

The following are available online at https://www.mdpi.com/article/10.3390/plants10112438/s1. Figure S1: Multiple nucleotide sequence alignments of the concatenated sequences of psbK-psbI, trnH-psbA, and atpF-atpH intergenic spacer region, and *matK*, *rpoC1*, and *rbcL* genes generated using ClustalW alignment software. The identical nucleotides are shaded in black; Figure S2: Divergence score matrix of the concatenated sequences of psbK-psbI, trnH-psbA, and atpF-atpH intergenic spacer region, and *matK*, *rpoC1*, and *rbcL* gene, based on a ClustalW alignment with the BLOSUM62 matrix; Table S1: All sequences with their accessions number used for species identification.
