# Supplementary material for: Morphological Characterization and DNA Barcoding of Duckweed Species in Saudi Arabia
Source: Plants (Basel). 2021 Nov 12;10(11):2438. doi: 10.3390/plants10112438 (PMC8620982; doi:10.3390/plants10112438)
Supplement: Supplementary file 1 [file plants-10-02438-s001.zip › plants-1398840-supplementary.pdf]

```
                                   10        20        30        40        50        60        70        80        90
                           ....|....|....|....|....|....|....|....|....|....|....|....|....|....|....|....|....|....|
Riyadh clone          1    GTTTGGCAAGCTGCTGTAAGTTTTCGATAAAGTTATTAAAACTCTACTGAAAAAATTCATGATTTATTTGATAAAAAAGATT-------- 82
Dhahran clone         1    ----------------------TTCGATAAAGTTATTAAAACTCTACTGAAAAAATTCATGATTTATTTGATAAAAAA-GATT------- 60
Tohama clone          1    -GTTGGCAAGCTGCTGTAAGTTTTCGATAAAGTTATTAAAACTCTACTGAAAAAATTCATGATTTATTTGATAAAAAA-GATT------- 81
Al-Baha clone         1    -TTTGGCAAGCTGCTGTAAGTTTTCGATAAAGTTATTAAAACTCTACTGAAAAAATTCATGATTTATTTGATAAAAAA-GATT------- 81
Jazan clone           1    ----GGCAAGCTGCTGTAAGTTTTCGATAAAGTCATTAAAACTCTACTGAAAAA-TTCATGATTTATTTGATAAAAAAAGATT------- 78
Al-Taif clone         1    -TTTGGCAAGCTGCTGTAAGTTTTCGATAAAGTTATTAAAACTCTACTGAAAAAATTCATGATTTATTTGATAAAAAA-GATT------- 81
Tanomah clone         1    ----TGCAAGCTGCTGTAAGTTTTCGATAAAGTTATTAAAACTCTACTGAAAAAATTCATGATTTATTTGATAAAAAA-GATT------- 78
Al-Qassim clone       1    -GTGGCAAGCTTGCTGTAAGTTTTCGATGAAGTTATTAAAACTCTATTGAAAAAATTCATGATTTATTTGATAAAAAAAGATT------- 82
Madinah clone 1       1    -----------------------------------------CTCTACTGAAAAA-TTCATGATTTATTTGATAAAAAAAGATT------- 41
Madinah clone 2       1    --------AGCTGCTGTAAGTTTTCGATGAAGTTCTTAATACTCTACTGAAAAAATTCATGATTTTTTTGATAAAAAAGGATTCTTATAT 82
Lemna japonica        1    GTTTGGCAAGCTGCTGTAAGTTTTCGATAAAGTTATTAAAACTCTACTGAAAAAATTCATGATTTATTTGATAAAAAA-GATT------- 82
Lemna perpusilla      1    -----------------------------------------CTCTACTGAAAAA-TTCATGATTTATTTGATAAAAAAAGATT------- 41
Landoltia punctata    1    ---------------GTAAGTTTTCGATGAAGTTATTAAAACTCTATTGAAAAAATTCATGATTTATTTGATAAAAAAAGATT------- 68
Spirodela polyrhiza   1    ---------------GTAAGTTTTCGATGAAGTTCTTAATACTCTACTGAAAAAATTCATGATTTTTTTGATAAAAAAGGATTCTTATAT 75
Lemna gibba           1    ---------------------------------------AACTCTACTGAAAAAATTCATGATTTATTTGATAAAAAA-GATT------- 43
Lemna aequinoctialis  1    ---------------GTAAGTTTTCGATAAAGTCATTAAAACTCTACTGAAAAAATTCATGATTTATTTGATAAAAAAAGATTCTAATAA 75
Lemna minor           1    ---------------GTAAGTTTTCGATAAAGTTATTAAAACTCTACTGAAAAAATTCATGATTTATTTGATAAAAAAGATT-------- 67

                                  100       110       120       130       140       150       160       170       180
                           ....|....|....|....|....|....|....|....|....|....|....|....|....|....|....|....|....|....|
Riyadh clone          83   C--------------TAATAAAAATTGATAACGTAATAACAATCTTAGTTTATACAT------CCTCAT-----AAAAAATA--TTTGAA 145
Dhahran clone         61   C--------------TAATAAAAATTGATAACGTAATAACAATCTTAGTTTA------TACATCCTCAT-----AAAAAAT--ATTTGAA 123
Tohama clone          82   C--------------TAATAAAAATTGATAACGTAATAACAATCTTAGTTTA------TACATCCTCAT-----AAAAAAT--ATTTGAA 144
Al-Baha clone         82   C--------------TAATAAAAATTGATAACGTAATAACAATCTTAGTTTA------TACATCCTCAT-----AAAAAAT--ATTTGAA 144
Jazan clone           79   C--------------TAATAATAATTGATAACGTAATAGCAATCTTAGTTTA------TACATCCTCAT-----AAAAAAT--AGGTGAA 141
Al-Taif clone         82   C--------------TAATAAAAATTGATAACGTAATAACAATCTTAGTTTA------TACATCCTCAT-----AAAAAAT--ATTTGAA 144
Tanomah clone         79   C--------------TAATAAAAATTGATAACGTAATAACAATCTTAGTTTA------TACATCCTCAT-----AAAAAAT--ATTTGAA 141
Al-Qassim clone       83   C--------------TAATAAA----GATAAGATAATATCAATTTTACGTTA------TACATCCTCAT-----TCCAAAA--TTTGGAA 141
Madinah clone 1       42   C--------------TAATAATAATTGATAACGTAATAGCAATCTTAGTTTA------TACATCCTCAT-----AAAAAAT--AGGTGAA 104
Madinah clone 2       83   C--------------TAATAATAATTGATAACATAATATGAGTCTTAGCTTATAAATATAAATCCTCATTAGAAAAGAAATAAATTTAAA 158
Lemna japonica        83   C--------------TAATAAAAATTGATAACGTAATAACAATCTTAGTTTA------TACATCCTCAT-----AAAAAAT--ATTTGAA 145
Lemna perpusilla      42   C--------------TAATAATAATTGATAACGTAATAGCAATCTTAGTTTA------TACATCCTCAT-----AAAAAAT--AGGTGAA 104
Landoltia punctata    69   C--------------TAATAAA----GATAAGATAATATCAATTTTACGTTA------TACATCCTCAT-----TCCAAAA--TTTGGAA 127
Spirodela polyrhiza   76   C--------------TAATAATAATTGATAACATAATATGAGTCTTAGCTTATAAATATAAATCCTCATTAGAAAAGAAATAAATTTAAA 151
Lemna gibba           44   C--------------TAATAAAAATTGATAACGTAATAACAATCTTAGTTTA------TACATCCTCAT-----AAAAAAT--ATTTGAA 106
Lemna aequinoctialis  76   TAATTGATAAGGTAATAGTAATAATTGATAAGGTAATAGCAATCTTAGTTTATACAG------CCTCAT-----AAAAAATA--TGTGAA 152
Lemna minor           68   C--------------TAATAAAAATTGATAACGTAGTAGCAATCTTAGTTTA------TACATCCTCAT-----AAAAAAT--ATTTGAA 130

                                  190       200       210       220       230       240       250       260       270
                           ....|....|....|....|....|....|....|....|....|....|....|....|....|....|....|....|....|....|
Riyadh clone          146  TTCTTGTATATTGGATAAAAAGAGCGATAAGTTTGGATCAGTCCATTTCACCGTTCTGGACGCTCTTCCAGTGAGGAAGTACTTTATTTT 235
Dhahran clone         124  TTCTTGTATATTGGATAAAAAGAGCGATAAGTTTGGATCAGTCCATTTCACCGTTCTGGACGCTCTTCCAGTGAGGAAGTACTTTATTTT 213
Tohama clone          145  TTCTTGTATATTGGATAAAAAGAGCGATAAGTTTGGATCAGTCCATTTCACCGTTCTGGACGCTCTTCCAGTGAGGAAGTACTTTATTTT 234
Al-Baha clone         145  TTCTTGTATATTGGATAAAAAGAGCGATAAGTTTGGATCAGTCCATTTCACCGTTCTGGACGCTCTTCCAGTGAGGAAGTACTTTATTTT 234
Jazan clone           142  TTCTTGTATATTGGATAAAAAGAGCGCTAAGTTTGGATC--------TTGCCGTTCTAGCCGCTCTTCCAGTGAGTAACTACTT------ 217
Al-Taif clone         145  TTCTTGTATATTGGATAAAAAGAGCGATAAGTTTGGATCAGTCCATTTCACCGTTCTGGACGCTCTTCCAGTGAGGAAGTACTTTATTTT 234
Tanomah clone         142  TTCTTGTATATTGGATAAAAAGAGCGATAAGTTTGGATCAGTCCATTTCACCGTTCTGGACGCTCTTCCAGTGAGGAAGTACTTTATTTT 231
Al-Qassim clone       142  TTCTTGTATATTGAATAAAAAGAGCGATAAGTTTTGATCAGTCCATTTCGGCCTTCTGGAGGCTCTTCCAGTGAGCAAGTACTTTATTT- 230
Madinah clone 1       105  TTCTTGTATATTGGATAAAAAGAGCGCTAAGTTTGGATC--------TTGCCGTTCTAGCCGCTCTTCCAGTGAGTAACTACTTTATT-- 184
Madinah clone 2       159  TTCTTGTATATTGGATAAAAGGAGCGATAAGTTTGGATCAGTCAATTTCCCCGTTCCGTACGCTCTTCCAGTGAGCAAGTACTTT----C 244
Lemna japonica        146  TTCTTGTATATTGGATAAAAAGAGCGATAAGTTTGGATCAGTCCATTTCACCGTTCTGGACGCTCTTCCAGTGAGGAAGTACTTTATTTT 235
Lemna perpusilla      105  TTCTTGTATATTGGATAAAAAGAGCGCTAAGTTTGGATC--------TTGCCGTTCTAGCCGCTCTTCCAGTGAGTAACTACTT------ 180
Landoltia punctata    128  TTCTTGTATATTGAATAAAAAGAGCGATAAGTTTTGATCAGTCCATTTCGGCCTTCTGGAGGCTCTTCCAGTGAGCAAGTACTTTATT-- 215
Spirodela polyrhiza   152  TTCTTGTATATTGGATAAAAGGAGCGATAAGTTTGGATCAGTCAATTTCCCCGTTCCGTACGCTCTTCCAGTGAGCAAGTACTTTTCTTTA 241
Lemna gibba           107  TTCTTGTATATTGGATAAAAAGAGCGATAAGTTTGGATCAGTCCATTTCACCGTTCTGGACGCTCTTCCAGTGAGGAAGTACTTTATTTT 196
Lemna aequinoctialis  153  TTCTTGTATATTGGATAAAAAGAGGGCTAAGTTTGGATC--------TTGCCGTTCTAGCCGCTCTTCCAGTGAGTAACTACTTT----- 229
Lemna minor           131  TTCTTGTATATTGGATAAAAAGAGCGATAAGTTTGGATCAGTCCATTTCGCCGTTCTGGCCGCTCTTCCAGTGAGGAAGTACTTTATTTA 220

                                  280       290       300       310       320       330       340       350       360
                           ....|....|....|....|....|....|....|....|....|....|....|....|....|....|....|....|....|....|
Riyadh clone          236  TTTATTAGCTTTTGTTTTAC-ACAATACTTTATTGTTATA-------ATTACAGTTAATTTTAGAATAAGCTTTTCCGTAATGAACAAGT 317
Dhahran clone         214  TTTATTAGCTTTTGTTTTAC-ACAATACTTTATTGTT--ATA-----ATTACAGTTAATTTTAGAATAAGCTTTTCCGTAATGAACAAGT 295
Tohama clone          235  TTTATTAGCTTTTGTTTTAC-ACAATACTTTATTGTT--ATA-----ATTACAGTTAATTTTAGAATAAGCTTTTCCGTAATGAACAAGT 316
Al-Baha clone         235  TTTATTAGCTTTTGTTTTAC-ACAATACTTTATTGTTATA-------ATTACAGTTAATTTTAGAATAAGCTTTTCCGTAATGAACAAGT 316
Jazan clone           217  --TATTAGCTTTTGTTTTAC-ACAAAACTTTATTGTTATATT-----ATTAGAGTTAATAGTCGAATAACCTTTTGGATAATGAACAAGT 299
Al-Taif clone         235  TTTATTAGCTTTTGTTTTAC-ACAATACTTTATTGTTATA-------ATTACAGTTAATTTTAGAATAAGCTTTTCCGTAATGAACAAGT 316
Tanomah clone         232  TTTATTAGCTTTTGTTTTAC-ACAATACTTTATTGTTATA-------ATTACAGTTAATTTTAGAATAAGCTTTTCCGTAATGAACAAGT 313
Al-Qassim clone       230  ---ATTAGCTTCTGTTTTCC-ACAATAATTTATTG----AAT-----ATTAGAGTTAGTATTAGAAAAACCTTTTGGGTAACGAACAAAT 307
Madinah clone 1       184  ------AGCTTTTGTTTTAC-ACAAAACTTTATTGTTATATT-----ATTAGAGTTAATAGTCGAATAACCTTTTGGATAATGAACAAGT 262
Madinah clone 2       245  TTTATTAGCTTATGTTTTTCCACAATACTTTATTGTTA-------------------ATATTAGAATAACCATTTTGGTAACGAACAAAT 315
Lemna japonica        236  TTTATTAGCTTTTGTTTTAC-ACAATACTTTATTGTTATA-------ATTACAGTTAATTTTAGAATAAGCTTTTCCGTAATGAACAAGT 317
Lemna perpusilla      180  --TATTAGCTTTTGTTTTAC-ACAAAACTTTATTGTTATATT-----ATTAGAGTTAATAGTCGAATAACCTTTTGGATAATGAACAAGT 262
Landoltia punctata    215  --TATTAGCTTCTGTTTTCC-ACAATAATTTATTGA-ATA--------TTAGAGTTAGTATTAGAAAAACCTTTTGGGTAACGAACAAAT 293
Spirodela polyrhiza   242  TT----AGCTTATGTTTTTCCACAATACTTTATTGTTAAT-------ATTAGA------------ATAACCATTTTGGTAACGAACAAAT 308
Lemna gibba           197  TTTATTAGCTTTTGTTTTAC-ACAATACTTTATTGTTATA-------ATTACAGTTAATTTTAGAATAAGCTTTTCCGTAATGAACAAGT 278
Lemna aequinoctialis  229  ---ATTAGCTTTTGTTTTAC-ACAAAACTTTATTGTTATATTATATTATTAGAGTTAATAGTCGAATAACCTTTTGGATAATGAACAAGT 315
Lemna minor           221  TTTATTAGCTTTTGTTTTAC-ACAATACTTTATTGTTAAT-------ATTAGAGTTAATATTAGAATAACCTTTTTGGTAACGAACAAGT 302

                                  370       380       390       400       410       420       430       440       450
                           ....|....|....|....|....|....|....|....|....|....|....|....|....|....|....|....|....|....|
Riyadh clone          318  CATAATCTTAATTT--AA---AATGCATTCATGAGTTTGAAAATTCAGTTTTTGTAGAAAAAAA------ACTTAACTTAAGACTATAAA 396
Dhahran clone         296  CATAATCTTAATTTAAAATGCA-----TTCATGAGTTTGAAAATTCAGTTTTTGTAGAAAAAAA------ACTTAACTTAAGACTATAAA 374
```

```
Tohama clone          317 CATAATCTTAATTTAAAATGCA-----TTCATGAGTTTGAAAATTCAGTTTTTGTAGAAAAAAA------ACTTAACTTAAGACTATAAA 395
Al-Baha clone         317 CATAATCTTAATTTAAA-----ATGCATTCATGAGTTTGAAAATTCAGTTTTTGTAGAAAAAAA------ACTTAACTTAAGACTATAAA 395
Jazan clone           300 TATAATCTTAATTCAAA-------AAATTCATGAATTTGAAAATTCAGTTTTTCTAGAAAAAAC------ACTGAATACTTAATT-CAAT 375
Al-Taif clone         317 CATAATCTTAATTTAAA-----ATGCATTCATGAGTTTGAAAATTCAGTTTTTGTAGAAAAAAA------ACTTAACTTAAGACTATAAA 395
Tanomah clone         314 CATAATCTTAATTTAAA-----ATGCATTCATGAGTTTGAAAATTCAGTTTTTGTAGAAAAAAA------ACTTAACTTAAGACTATAAA 392
Al-Qassim clone       308 CATAATCTTAATTTCAACAACAAAAAATTCATGAATTTTGTAATTCAGTTTTTTTAGAAAAAAA------CACT-----------TAAT 379
Madinah clone 1       263 TATAATCTTAATTCAAAAAA-------TTCATGAATTTGAAAATTCAGTTTTTCTAGAAAAAAC------ACTGAATACTTAATT-CAAT 338
Madinah clone 2       316 CATAATCTTAATTTCAGA---AAAAAATTCATGAATTTGAAAATTCAGTTTTTTTAGAAAAAAC------ACTTAATTAAAAA----AAT 392
Lemna japonica        318 CATAATCTTAATTTAAA-----ATGCATTCATGAGTTTGAAAATTCAGTTTTTGTAGAAAAAAA------ACTTAACTTAAGACTATAAA 396
Lemna perpusilla      263 TATAATCTTAATTCAAA-----AA--ATTCATGAATTTGAAAATTCAGTTTTTCTAGAAAAAAC------ACTGAATACTTAATT-CAAT 338
Landoltia punctata    294 CATAATCTTAATTTCAACAACAAAAAATTCATGAATTTTGTAATTCAGTTTTTTTAGAAAAAA------------ACACT------TAAT 365
Spirodela polyrhiza   309 CATAATCTTAATTTCAGA---AAAAAATTCATGAATTTGAAAATTCAGTTTTTTTAGAAAAAA-----------------CACTTAATT 377
Lemna gibba           279 CATAATCTTAATTTAAA-----ATGCATTCATGAGTTTGAAAATTCAGTTTTTGTAGAAAAAAA------ACTTAACTTAAGACTATAAA 357
Lemna aequinoctialis  316 TATAATCTTAATTC-------AAAAAATTCATGAATTTGAAAATTCAGTTTTTCTAGAAAAAAA------CACTGAATACTTAATTCAAT 392
Lemna minor           303 CATAATCTTAATTT--AA---AATGCATTCATGAGTTTGAAAATTCAGTTTTTGTAGAAAAAAACACTAAACTTAATACTATACTTAATA 387

                                 460       470       480       490       500       510       520       530       540
                          ....|....|....|....|....|....|....|....|....|....|....|....|....|....|....|....|....|....|
Riyadh clone          397 AAAGAAAGGATTGTTCTTTATTTTT-TCATAGTTTTTTCTT--------GGCATGCCAAAATAATATATGTGTT-ACATAACTCAAATGG 476
Dhahran clone         375 AAAGAAAGGATTGTTCTTTATTTTTTCATAGTTTTTTCTT---------GGCATGCCAAAATAATATATGTGTT-ACATAACTCAAATGG 454
Tohama clone          396 AAAGAAAGGATTGTTCTTTATTTTTTCATAGTTTTTTCTT---------GGCATGCCAAAATAATATATGTGTT-ACATAACTCAAATGG 475
Al-Baha clone         396 AAAGAAAGGATTGTTCTTTATTTTTTCATAGTTTTTTCTT---------GGCATGCCAAAATAATATATGTGTT-ACATAACTCAAATGG 475
Jazan clone           376 TAAAAAACAATTGTTCTTTTTTTCACTGTTTTTTTTTATT---------CGCATGTCAAACTAATACATGTGTTTACATAACTGAAATGG 456
Al-Taif clone         396 AAAGAAAGGATTGTTCTTTATTTTTTCATAGTTTTTTCTT---------GGCATGCCAAAATAATATATGTGTT-ACATAACTCAAATGG 475
Tanomah clone         393 AAAGAAAGGATTGTTCTTTATTTTTTCATAGTTTTTTCTT---------GGCATGCCAAAATAATATATGTGTT-ACATAACTCAAATGG 472
Al-Qassim clone       380 AAAGAAAAAATTGTTCTTTATTTTTACTAGTTTTTTTTGT---------GGCATGTCAAAATAAGACATGTGTT-ACATAACTCAAATGG 459
Madinah clone 1       339 TAAAAAACAATTGTTCTTTTTTTCACTGTTTTTTTTTATT---------CGCATGTCAAACTAATACATGTGTTTACATAACTGAAATGG 419
Madinah clone 2       393 TTGTTCTTTATTTTTTCATATTTTT-TTTTTGTTTTTGGT---------GGCATGTCAAAATAATACATGTGTT-ACATAACTCAAATGG 471
Lemna japonica        397 AAAGAAAGGATTGTTCTTTATTTTTTCATAGTTTTTTCTT---------GGCATGCCAAAATAATATATGTGTT-ACATAACTCAAATGG 476
Lemna perpusilla      339 TAAAAAACAATTGTTCTTTTTTTCACTGTTTTTTTTTATT---------CGCATGTCAAACTAATACATGTGTTTACATAACTGAAATGG 419
Landoltia punctata    366 AAAGAAAAAATTGTTCTTTATTTTTACTAGTTTTTTTTGT---------GGCATGTCAAAATAAGACATGTGTT-ACATAACTCAAATGG 445
Spirodela polyrhiza   378 AAA--AAAATTGTTCTTTATTTTTTCATATTTTTTTTTGTTTTTGGTGGCATGTCAAAATAATACATGTGTT-ACATAACTCAAATGG 464
Lemna gibba           358 AAAGAAAGGATTGTTCTTTATTTTTTCATAGTTTTTTCTT---------GGCATGCCAAAATAATATATGTGTT-ACATAACTCAAATGG 437
Lemna aequinoctialis  393 TAAAAAACAATTGTTCTTTTTTTCACTGTTTTTTTTTATT--------CGCATGTCAAACTAATACATGTGTT-ACATAACTGAAATGG 473
Lemna minor           388 AAATAAAGAATTGTTCCTTATTTTTTCATAGTTTTTTTTCTT-------GGCATGCCCAAATAATACATGTGTT-ACATAACTCAAATGG 469

                                 550       560       570       580       590       600       610       620       630
                          ....|....|....|....|....|....|....|....|....|....|....|....|....|....|....|....|....|....|
Riyadh clone          477 ATAATCTATTCCCTTTTACCCCAAAAATGATCCTATCTTGGAGATTGTGTAATGCTTAC----TCGTTTGGCAAGCTGCTGTAAGTTTTC 562
Dhahran clone         455 ATAATCTATTCCCTTTTACCCCAAAAATGATCCTATCTTGGAGATTGTGTAATGCTTAAC-GTAATGCTCACAACTTCCCTCTAGACTTA 543
Tohama clone          476 ATAATCTATTCCCTTTTACCCCAAAAATGATCCTATCTTGGAGATTGTGTA-----------------CACAACTTCCCTCTAGACTTA 547
Al-Baha clone         476 ATAATCTATTCCCTTTTACCCCAAAAATGATCCTATCTTGGAGATTGTGTAATGCT---------------------------------- 531
Jazan clone           457 ATAATCTATTCCCTTTTACTCCAAAAATGATCCAATCTTGGAGATTGTGTAATGTCAC--------------AACTTCCCCCTAGAATTA 532
Al-Taif clone         476 ATAATCTATTCCCTTTTACCCCAAAAATGATCCTATCTTGGAGATTGTGTAATGCTTA-TTGTAATGCTCACAACTTCCCTCTAGACTTA 564
Tanomah clone         473 ATAATCTATTCCCTTTTACCCCAAAAATGATCCTATCTTGGAGATTGTGTAATGCTA---TGTAATGCTCACAACTTCCCTCTAGACTTA 559
Al-Qassim clone       460 ATAATCTATTCCCTTTTACCCCAAAAATGATCCTATCTTGGAGATTGTGTAATGCT-----GTAATGCTCACAACTTCCCTCTAGACTTA 544
Madinah clone 1       420 ATAATCTATTCCCTTTTACTCCAAAAATGATCCAATCTTGGAGATTGTGTAATGCTTAC-----TTGGCTACATCCGCCCCCTT-ACTTA 503
Madinah clone 2       472 ATAATCTATTCCCTTTTACCCCAAAAATGATCCTATCTTGGAGATTGTGTAGAACGTAA----TG--CTCACAACTTCCCTCTAGACTTA 555
Lemna japonica        477 ATAATCTATTCCCTTTTACCCCAAAAATGATCCTATCTTGGAGATTGTGTAATGCTTACTCATTCACAATCCACTGCCTTGATCCACTTG 566
Lemna perpusilla      420 ATAATCTATTCCCTTTTACTCCAAAAATGATCCAATCTTGGAGATTGTGTAATGCTT--------------------------------- 476
Landoltia punctata    446 ATAATCTATTCCCATTCACAATCCAC-TGCCTTGATCCACTTGGCTACATCCGCCCC--------------------------------- 501
Spirodela polyrhiza   465 ATAATCTATTCCCATTCACAATCCAC-TGCCTTGATCCACTTGGCTACATCCGCCCCCTTACTTAAGCTTAGCTAAAGTATTTCACCTTT 553
Lemna gibba           438 ATAATCTATTCCCTTTTACCCCAAAAATGATCCTATCTTGGAGATTGTGTAATGCTTA----TTCACAATCCACTGCCTTGATCCACTTG 523
Lemna aequinoctialis  474 ATAATCTATTCCCATTCAC---------AATCC----------ACTGCCTTGA----------------------TCC------ACTTG 515
Lemna minor           470 ATAATCTATTCCCATTCACAATCCAC-TGCCTTGATCCACTTGGCTACATCCGCCCCCTTACTTAAGCTTAGCTAAAGTATTTCACTTTT 558

                                 640       650       660       670       680       690       700       710       720
                          ....|....|....|....|....|....|....|....|....|....|....|....|....|....|....|....|....|....|
Riyadh clone          563 GATAAAGTTATTAAAACTCTACTGAAAAAATTCATGATTTATTTGATAAAAAAGATTCTAATAAAAATTGATAACGTAATAACAATCTTA 652
Dhahran clone         544 GCTGCAGT---TGAAGCTCCATCTACCAT-TGGATAAGACTTTTTGTCTTAGTGT-----ATAGGG------------------------ 600
Tohama clone          548 GCTGCAGT---TGAAGCTCCATCTACCAT-TGGATAAGACTTTTTGTCTTAGTGT-----ATAGGG------------------------ 604
Al-Baha clone         531 ------------------------------------------------------------------------------------------ 531
Jazan clone           533 GGGGCAGT---TGAAGCTCCATCTACCAT-TGGATAAGATTTTTTGTCTTAGTGT-----ATAGGG------------------------ 589
Al-Taif clone         565 GCTGCAGT---TGAAGCTCCATCTACCAT-TGGATAAGACTTTTTGTCTTAGTGT-----ATAGGG------------------------ 621
Tanomah clone         560 GCTGCAGT---TGAAGCTCCATCTACCAT-TGGATAAGACTTTTTGTCTTAGTGT-----ATAGGG------------------------ 616
Al-Qassim clone       545 GCTGCAGT---TGAAGCTCCATCTACCAT-TGGATAAGACTTTTTGTCTTAGTGT-----ATAGGG------------------------ 601
Madinah clone 1       504 AGCTTAGC---TAAAGT---ATTTAA-GT-TTTTTATTTCTTTTTTTTTTTTTAC------TAAAA------------------------ 555
Madinah clone 2       556 GCTGCAGT---TGAAGCTCCATCTACCAT-TGGATAAGACTTTTTGTCTTAGTGT-----ATAGGGCTTGATAAAGGAATAATA------ 630
Lemna japonica        567 GCTACATC---CGCCCCCTTACTTAAGCT-TAGCTAAAGTATTTCACTTTTTTTT-----A----------------------------- 618
Lemna perpusilla      476 ------------------------------------------------------------------------------------------ 476
Landoltia punctata    501 -----------------CTTACTTAAGCT-TAGCTAAAGTATTGCACTTTTTTAT----------------------------------- 538
Spirodela polyrhiza   554 TTTGTTTC---TTTTATTTTTTAAATATT-AAAATTGGATTTCTTTTTTCCTTGA-----ATTATATTATAGATTTAAGATTTAATTATA 634
Lemna gibba           524 GCTACATC---CGCCCCCTTACTTAAGCT-TAGCTAAAGTATTTCACTTTTT--T-----A----------------------------- 573
Lemna aequinoctialis  516 GCTA------------CTCCGCCC----------------------CCTTACT--------TAAGCTTAGCTAAAGTATTTA-------- 555
Lemna minor           559 TTTT----------------------------------ATTTCTTTTTTACT-------------------------------------- 576

                                 730       740       750       760       770       780       790       800       810
                          ....|....|....|....|....|....|....|....|....|....|....|....|....|....|....|....|....|....|
Riyadh clone          653 GTTTATACATCCTCATAAAAAAATATTTGAATTCTTGTATATTGGATAAAAAGAGCGATAAGTTTGGATCAGTCCATTTCACCGTTCTGGA 742
Dhahran clone         600 ------------------------------------------------------------------------------------------ 600
Tohama clone          604 ------------------------------------------------------------------------------------------ 604
Al-Baha clone         531 ------------------------------------------------------------------------------------------ 531
Jazan clone           589 ------------------------------------------------------------------------------------------ 589
Al-Taif clone         621 ------------------------------------------------------------------------------------------ 621
Tanomah clone         616 ------------------------------------------------------------------------------------------ 616
Al-Qassim clone       601 ------------------------------------------------------------------------------------------ 601
Madinah clone 1       555 ------------------------------------------------------------------------------------------ 555
Madinah clone 2       630 --CCAAACCTCCTTATTAGAGGT-TTGGTATTGCTCCTTTTGTGTGAATTAGTGC----ACTCTTATTTTGTCTACATAAGGATTTTTGA 713
```

```
Lemna japonica        618  ---------------------------------------------------------------------------------------TTT 621
Lemna perpusilla      476  ------------------------------------------------------------------------------------------ 476
Landoltia punctata    538  ---------------------------------------------------------------------------------------TTC 541
Spirodela polyrhiza   635  TTATAGTTATAGATTTTATTTAT---ATAATAGATATATTTATATAATATGTAAATATCAAATATGTAAATATAATTAAATAAAGGATTT 721
Lemna gibba           573  ---------------------------------------------------------------------------------------TTT 576
Lemna aequinoctialis  555  ------ACTTTTTTAT---------------TTCTTTTTTTTTTTTTACTAAAAC--------------TACAAATGTAAAAA------A 604
Lemna minor           576  ------------------------------------------------------------------------------------------ 576

                                 820       830       840       850       860       870       880       890       900
                           ....|....|....|....|....|....|....|....|....|....|....|....|....|....|....|....|....|....|
Riyadh clone          743  CGCTCTTCCAGTGAGGAAGTACTTTATTTTTTTATTAGCTTTTGTTTTACA-CAATACTTTATTGTTATAATTACAGTTAATTTTAGAAT 831
Dhahran clone         600  -CTTGATGACGGAAT------------------------------------------------------AATACCAAACCTCTTTTTTAG 635
Tohama clone          604  -CTTGATGACGGAAT------------------------------------------------------AATACCAAACCTCTTTTTTAG 639
Al-Baha clone         531  ------------------------------------------------------------------------------------------ 531
Jazan clone           589  -CTTTATGAAGGAAT------------------------------------------------------AATACCAAACCTCCTTATTAG 624
Al-Taif clone         621  -CTTGATGACGGAAT-----------------------------------------------------AATACCAAACCTCTTTTTT-AG 656
Tanomah clone         616  -CTTGATGACGGAAT-----------------------------------------------------AATACCAAACCTCTTTTTT-AG 651
Al-Qassim clone       601  -CTTGATCAAGGAAT------------------------------------------------------AATACCAAACCTCTAATAAAG 636
Madinah clone 1       555  -CTACAAATGTAAAA------------------------------------------------------AATACTTA--TGTAGACAAAA 588
Madinah clone 2       714  CATT--TGTACTTAGCA--TACTTTGTATTTTTGTCAATTTGTAATTAATGTCCATTCTTTCGTTTTGGAATGAAAGAAATCCTTTATTT 799
Lemna japonica        622  CTTTTTTACTAAAAC--------------------------------------------------TACAAATGTAAAAAATCCTTAT-GT 660
Lemna perpusilla      476  ------------------------------------------------------------------------------------------ 476
Landoltia punctata    542  TTTTATGCCTGAAAC--------------------------------------------------TCGAAATGTCAAAAATCCTTAT-GT 580
Spirodela polyrhiza   722  CTTTCATTCCAAAACGAAAGAATGGACATTAATTACAAATTGACAAAAATACAAAGTATGCTAAGTACAAATGTCAAAAATCCTTAT-GT 810
Lemna gibba           577  CTTTTTTACTAAAAC--------------------------------------------------TACAAATGCAAAAAATACTTAT-GT 615
Lemna aequinoctialis  605  TACTTATGTAGACAAAA-------TATCATTGTACTAACTCATAGAAAA------------------GGAGTAATACCAAACCTCTAATA 669
Lemna minor           576  ----------AAAAC--------------------------------------------------TACAAATGTAAAAAATCCTTAT-GT 605

                                 910       920       930       940       950       960       970       980       990
                           ....|....|....|....|....|....|....|....|....|....|....|....|....|....|....|....|....|....|
Riyadh clone          832  AA--GCTTTTCCGTAATGAACAAGTCATAATCTTAATTTAAAATGCATTCATGAGTTTGAAAATTCAGTTTTTGTAGAAAAAAAACTTAA 919
Dhahran clone         636  AGGTTTGGTATTACTCCTT------GCGT-TAATAGAATGGTATTTTGTCTACA-TAAGTATTTTTTGC---------ATTTGTAGTTTT 708
Tohama clone          640  AGGTTTGGTATTACTCCTT------GCGT-TAATAGAATGGTATTTTGTCTACA-TAAGTATTTTTTGC---------ATTTGTAGTTTT 712
Al-Baha clone         531  ------------------------------------------------------------------------------------------ 531
Jazan clone           625  AGGTTTGGTATTACTCCTTTTCTATGAGT-TAGTACAATGATATTTTGTCTACATAAGTATTTTTTACA---------TTTGTAGTTTTA 704
Al-Taif clone         657  AGGTTTGGTATTACTCCTTGCGTTAAT-------AGAATGGTATTTTGTCTACATAAGTATTTTTTGCA---------TTTGTAGTTTTA 730
Tanomah clone         652  AGGTTTGGTATTACTCCTTGCGTTAAT-------AGAATGGTATTTTGTCTACATAAGTATTTTTTGCA---------TTTGTAGTTTTA 725
Al-Qassim clone       637  AGGTTTGGTATTACTCCTTTTGTATGAGT-TAGTACAATGGTATTTTGTCTACA-TAAGGATTTTTGAC---------ATTTCGAGTTTC 715
Madinah clone 1       589  TACCATTGTACTAACTCAT--------AG-AAAAGGAGTAATACCAAACCTCTAATAAGGAGGTTTGGT---------ATTATTCCTTCA 660
Madinah clone 2       800  AATTATATTTACATATTTGATATTTACATATTATATAAATATATCTATTATATAAATAAAATCTATAACTATAATATAATTAAATCTTAA 889
Lemna japonica        661  AGACAAAATACCATTGTATTAACTCA-------AGGAGTAATACCAAACCTCCTTATTAGAGGTTTGGT---------ATTATTCCGTAA 734
Lemna perpusilla      476  ------------------------------------------------------------------------------------------ 476
Landoltia punctata    581  AGACAAAATACCATTGTACTAACTCATAC-AAAAGGAGTAATACCAAACCTCTTTATTAGAGGTTTGGT---------ATTATTCCTTGA 660
Spirodela polyrhiza   811  AGACAAAATAAGAGTGCACTAATTCACAC-AAAAGGAGCAATACCAAACCTCTAATAAGGAGGTTTGGT---------ATTATTCCTTTA 890
Lemna gibba           616  AGACAAAATACCATTCTATTAACGCA-------AGGAGTAATACCAAACCTCTAAAAAAGAGGTTTGGT---------ATTATTCCGTCA 689
Lemna aequinoctialis  670  AG---------GAGGTTTGGTATTATTCCTTCATAAAGCCCTATACACTA---AGACAAAA---------------------AATCTTA- 725
Lemna minor           606  AGACAAAATACCATTGTATTAACTCA-------AGGAGTAATACCAAACCTCCTTATTAGAGGTTTGGT---------ATTATTCCGTAA 679

                                1000      1010      1020      1030      1040      1050      1060      1070      1080
                           ....|....|....|....|....|....|....|....|....|....|....|....|....|....|....|....|....|....|
Riyadh clone          920  CTTAAGACTATAAAAAAGAAAGGATTGTTCTTTATTTTTTCATAGTTTTTTCTTGGCATGCCAAA-ATAATATATGTGTTACATAACTCA 1008
Dhahran clone         709  AG--TAAAAAAGAAATAAAAAAGTGAAATACTTTAGCT-------------AAGCTTAAGTAAGGGGGCG----------GATGTAGCCA 773
Tohama clone          713  AG--TAAAAAAGAAATAAAAAAGTGAAATACTTTAGCT-------------AAGCTTAAGTAAGGGGGCG----------GATGTAGCCA 777
Al-Baha clone         531  ------------------------------------------------------------------------------------------ 531
Jazan clone           705  GT---AAAAAAAAAAAAAGAAATAAAAAAGTTAAATAC-------------TTTAGC-----------TA----------AGCTTAAGTA 757
Al-Taif clone         731  GT---AAAAAAGAAATAAAAAAGTGAAATACTTTAGCT-------------AAGCTTAAGTAAGGGGGCG----------GATGTAGCCA 794
Tanomah clone         726  GT---AAAAAAGAAATAAAAAAGTGAAATACTTTAGCT-------------AAGCTTAAGTAAGGGGGCG----------GATGTAGCCA 789
Al-Qassim clone       716  AGG-CATAAAAGAAATAAAAAAGTGCAATACTTTAGCT-------------AAGCTTAAGTAAGGGGGCG----------GATGTAGCCA 781
Madinah clone 1       661  TA---AAGCCCTATACACTAAGATAAAAAATCTTATCC-------------AAT------------GGTA----------GATGGAGCT- 711
Madinah clone 2       890  ATCTATAATATAATTCAAGGAAAAAAGAAATCCAATTTT----AATATTTAAAAAATAAAAGAAACAAAAAAGGTGAAATACTTTAGCTA 975
Lemna japonica        735  TC---AAGCCCTATACACTAAGACAAAAAGTCTTATCC-------------AATGGTAGATGGAGCTTCA----------ACTGCAGCTA 798
Lemna perpusilla      476  ------------------------------------------------------------------------------------------ 476
Landoltia punctata    661  TC---AAGCCCTATACACTAAGACAAAAAGTCTTATCC-------------AATGGTAGATGGAGCTTCA----------ACTGCAGCTA 724
Spirodela polyrhiza   891  TC---AAGCCCTATACACTAAGACAAAAAGTCTTATCC-------------AATGGTAGATGGAGCTTCA----------ACTGCAGCTM 954
Lemna gibba           690  TC---AAGCCCTATACACTAAGACAAAAAGTCTTATCC-------------AATGGTAGATGGAGCTTCA----------ACTGCAGCTA 753
Lemna aequinoctialis  725  ------------------------------TCCAAT------------------GGTAGATGGAG----------------CTTCAACTG 751
Lemna minor           680  TC---AAGCCCTATACACTAAGACAAAAAGTCTTATCC-------------AATGGTAGATGGAGCTTCA----------ACTGCAGCTA 743

                                1090      1100      1110      1120      1130      1140      1150      1160      1170
                           ....|....|....|....|....|....|....|....|....|....|....|....|....|....|....|....|....|....|
Riyadh clone         1009  AATGGATAATCTATTCCCTTTTACCCCAAAAATGATCCTATCTTGGAGATTGTGTAATGCTTACTCTTTCCAAAATAAATTACTACACCA 1098
Dhahran clone         774  AG--TGGATCAAGGCAGTGGATTG-------TGAATCC---------ACCACTCGCACACACTCCCTTTCCAAAATAAATTACTACACCA 845
Tohama clone          778  AG--TGGATCAAGGCAGTGGATTG-------T--------------------------------CCTTTCCAAAATAAATTACTACACCA 826
Al-Baha clone         531  ------------------------------------------------------------------------------------------ 531
Jazan clone           758  AGGGGGCGGAGTAGCCAAGTGGAT-------CAAGGC--------------------AGTGGATTGTGATCAAAATAAATCCCTACCCCA 820
Al-Taif clone         795  AG--TGGATCAAGGCAGTGGATTG-------TGAC------------------------------------AAAATAAATTACTACACCA 839
Tanomah clone         790  AG--TGGATCAAGGCAGTGGATTG-------TGACT----------------------------CCTTTCCAAAATAAATTACTACACCA 842
Al-Qassim clone       782  AG--TGGATCAAGGCAGTGGATTG-------TGA------------------TCGCACACACTCCCTTTCCAAAATAAATCACTACACCA 844
Madinah clone 1       711  --------TCAACGCA-CACACT----------------------------------------CCCTTTCCAAAATAAATCCCTACCCCA 752
Madinah clone 2       976  AGCTTA-AGTAAGGGGGCGGATGTAGCCAAGTGGATC--------AAGGCAGTGGATTGTGAATCCTTTCCAAAATAAATCAATACACCA 1056
Lemna japonica        799  AGTCTAGAGGGAAGTTGTGAGCAT-------TACTTTA---------ACAATTTATGGACTGGTTGTGGCATTAGCACTTTTATTTGCGA 872
Lemna perpusilla      476  -----------------------------------------------------------------------TTAGCACTTTTATTTGCGA 495
Landoltia punctata    725  AGTCTAGAGGGAAGTTGTGAGCAT-------TAC----------------ACTCGCACACACTCCCTTTCCAAAATAAATCACTACACCA 791
Spirodela polyrhiza   955  AGTCTAGAGGGAAGTTGTGAGCAT-------TACA----------------CTCGCACACACTCCCTTTCCAAAATAAATCAATACACCA 1021
Lemna gibba           754  AGTCTAGAGGGAAGTTGTGAGCAT-------TAC----------------ACTCGCACACACTCCCTTTCCAAAATAAATTACTACACCA 820
Lemna aequinoctialis  752  CCCCTAATTCTAGGGGGAAGTTGT------GAGCATT-----------ACACTCGCACACACTCCCTTTCCAAAATAAATCCCTACCCCA 824
Lemna minor           744  AGTCTAGAGGGAAGTTGTGAGCAT-------TAC-------------------------------------------------------- 770
```

```
                                       1180      1190      1200      1210      1220      1230      1240      1250      1260
                                  ....|....|....|....|....|....|....|....|....|....|....|....|....|....|....|....|....|....|
Riyadh clone               1099 AGTACTACGCTTAGATTTATTGGATTTGTTGCTAAAATATCGG-TATTAAACCCAAAACCACCGGCGGATGGCCAGTGGC---CCAAGGA 1184
Dhahran clone              846  AGTACTACGCTTAGATTTATTGGATTTGTTGCTAAAATATCGG-TATTAAACCCAAAACCACCGGCGGATGGCCAGTGGC---CCAAGGA 931
Tohama clone               827  AGTACTACGCTTAGATTTATTGGATTTGTTGCTAAAATATCGG-TATTAAACCCAAAACCACCGGCGGATGGCCAGTGGC---CCAAGGA 912
Al-Baha clone              531  -------------ACCTCCCAA---------------------------------AATTCACAATCCACTGCCTTGA------TCCACTT 569
Jazan clone                821  AGTACTACGCTTAGATTTATTGGATTTGTTGCTAAAATATCGG-TATTAAACCCAAAACCCCCGGCAGACGGCCAGTGGC---CCAAGGA 906
Al-Taif clone              840  AGTACTACGCTTAGATTTATTGGATTTGTTGCTAAAATATCGG-TATTAAACCCAAAACCACCGGCGGATGGCCAGTGGC---CCAAGGA 925
Tanomah clone              843  AGTACTACGCTTAGATTTATTGGATTTGTTGCTAAAATATCGG-TATTAAACCCAAAACCACCGGCGGATGGCCAGTGGC---CCAAGGA 928
Al-Qassim clone            845  AGTACTACGCTTAGATTTATTGGATTTGTTGCTAAAATATCGG-TATTAAACCCAAAACCCACGGCGGATGGCCCCTGGG---CCAAGGA 930
Madinah clone 1            753  AGTACTACGCTTAGATTTATTGGATTTGTTGCTAAAATATCGG-TATTAAACCCAAAACCCCCGGCAGACGGCCAGTGGC---CCAAGGA 838
Madinah clone 2            1057 AGTACTACGCTTAGATTTATTGGATTTGTTGCTAAAATATCGG-TATTAAACCCAAAACCCCCGGCGGATGGCCAGTGGC---CCAAGGA 1142
Lemna japonica             873  ATCCATTTGTTTAATTCTAGAATCAAAAAAAGTACGTAATAGACTTTTTGACTTAGACTTGCTTTTTGCTTCTTCGAATTATATCAACAT 962
Lemna perpusilla           496  ATCCTTTTGTTTAATTCTACAA--AAAGAAAGTAC---------TTTTTGACTTAGACTTGCTTTTTGCTTCTTCGAATTCTATCAACAT 574
Landoltia punctata         792  AGTACTACGCTTAGATTTATTGGATTTGTTGCTAAAATATCGG-TATTAAACCCAAAACCCACGGCGGATGGCCCCTGGG---CCAAGGA 877
Spirodela polyrhiza        1022 AGTACTACGCTTAGATTTATTGGATTTGTTGCTAAAATATCGG-TATTAAACCCAAAACCCCCGGCGGATGGCCAGTGGC---CCAAGGA 1107
Lemna gibba                821  AGTACTACGCTTAGATTTATTGGATTTGTTGCTAAAATATCGG-TATTAAACCCAAAACCACCGGCGGATGGCCAGTGGC---CCAAGGA 906
Lemna aequinoctialis       825  AGTACTACGCTTAGATTTATTGGATTTGTTGCTAAAATATCGG-TATTAAACCCAAAACCCCCGGCAGACGGCCAGTGGC---CCAAGGA 910
Lemna minor                770  ----CTACGCTTAGATTTATTGGATTTGTTGCTAAAATATCGG-TATTAAACCCAAAACCGCCGGCGGATGGCCAGTGGC---CCAAGGA 852

                                       1270      1280      1290      1300      1310      1320      1330      1340      1350
                                  ....|....|....|....|....|....|....|....|....|....|....|....|....|....|....|....|....|....|
Riyadh clone               1185 AACAA---AAGAATCAGTTACATTTTGCATATACTCTCCTCTTATAGATAGGACTAAAAAAGAACAGAGTTCTGTTCTTTTTGTATTACT 1271
Dhahran clone              932  AACAA---AAGAATCAGTTACATTTTGCATATACTCTCCTCTTATAGATAGGACTAAAAAAGAACAGAGTTCTGTTCTTTTTGTATTACT 1018
Tohama clone               913  AACAA---AAGAATCAGTTACATTTTGCATATACTCTCCTCTTATAGATAGGACTAAAAAAGAACAGAGTTCTGTTCTTTTTGTATTACT 999
Al-Baha clone              570  GGC-----------TAC--ATCCG-----------CCCCTTA----------CTTAAGCTTAGC------TAAAGTATTTCACTTTTTT 619
Jazan clone                907  AACAA---AAGAATCAGTTACATTTTGCATATACTTTCCTCTTATAGATAGGACTAAAAAAGAACAG-----AGTTCTTTTTGTATTACT 988
Al-Taif clone              926  AACAA---AAGAATCAGTTACATTTTGCATATACTCTCCTCTTATAGATAGGACTAAAAAAGAACAGAGTTCTGTTCTTTTTGTATTACT 1012
Tanomah clone              929  AACAA---AAGAATCAGTTACATTTTGCATATACTCTCCTCTTATAGATAGGACTAAAAAAGAACAGAGTTCTGTTCTTTTTGTATTACT 1015
Al-Qassim clone            931  AACAA---AAGAATCAGTTACATTTTGCATATACTCTCCTCTTATAGATAGGACTAACAAAGAACAGAGTT-----CTTTTTGTATTACT 1012
Madinah clone 1            839  AACAA---AAGAATCAGTTACATTTTGCATATACTTTCCTCTTATAGATAGGACTAAAAAAGAACAGAGTT-----CTTTTTGTATTACT 920
Madinah clone 2            1143 AACAA---AAGAATCAGTTACATTTTTCATATACTCTCCTCTTATAGATAGGACTAACAAAGAACAGAGTT-----CTTTTTGTATCACT 1224
Lemna japonica             963  TGTACTCTAACAATTACTTATTCGTTGAGAGAATACCTCCGGGAAGGACTGATTTTAGGATTAGTAATTAGCAGATCCTCTCGCTTTCTT 1052
Lemna perpusilla           575  TGCACTCTAACAATTACTTATTCGTTGAGAGAATACCTCCGGGAAGGACTGATTTTAGGATTAGTAATTAGCAGATCCTCTCGTTTTCTT 664
Landoltia punctata         878  AACAA---AAGAATCAGTTACATTTTGCATATACTCTCCTCTTATAGATAGGACTAACAAAGAACAG-----AGTTCTTTTTGTATTACT 959
Spirodela polyrhiza        1108 AACAA---AAGAATCAGTTACATTTTTCATATACTCTCCTCTTATAGATAGGACTAACAAAGAACAG-----AGTTCTTTTTGTATCACT 1189
Lemna gibba                907  AACAA---AAGAATCAGTTACATTTTGCATATACTCTCCTCTTATAGATAGGACTAAAAAAGAACAGAGTTCTGTTCTTTTTGTATTACT 993
Lemna aequinoctialis       911  AACAA---AAGAATCAGTTACATTTTGCATATACTTTCCTCTTATAGATAGGACTAAAAAAGAACAGAGTT-----CTTTTTGTATTACT 992
Lemna minor                853  AACAA---AAGAATCAGTTACATTTTGCATATACTCTCCTCTTATAGATAGGACTAAAAAAGAACAG-----AGTTCCTTTTGTATTACT 934

                                       1360      1370      1380      1390      1400      1410      1420      1430      1440
                                  ....|....|....|....|....|....|....|....|....|....|....|....|....|....|....|....|....|....|
Riyadh clone               1272 TCGCCCCCTTTGTTTGATT-----TCTTTTTTTT-TATGGGATTTTAAAATGGAATAGATTAAATTAATTAATTTAA---TTGAGAACTT 1352
Dhahran clone              1019 TCGCCCCCTTTGTTTGATTTCTTTTTTTTT------ATGGGATTTTAAAATGGAATAGATTAAATTAATTAATTTAA---TTGAGAACTT 1099
Tohama clone               1000 TCGCCCCCTTTGTTTGATTTCTTTTTTTTT------ATGGGATTTTAAAATGGAATAGATTAAATTAATTAATTTAA---TTGAGAACTT 1080
Al-Baha clone              620  ATTTCT--TTT--TTACT----------------------------AAAACTACA---------------AATGCAA---AAAATACT-T 658
Jazan clone                989  TCGCCCCTTTTCTTTGATT---TCTTTTTTTTT-TATGGGATTTTAAAATAGAATAGATGACATTAATTAACTTAA---TTGAGAACTT 1071
Al-Taif clone              1013 TCGCCCCCTTTGTTTGATT-----TCTTTTTTTT-TATGGGATTTTAAAATGGAATAGATTAAATTAATTAATTTAA---TTGAGAACTT 1093
Tanomah clone              1016 TCGCCCCCTTTGTTTGATT-----TCTTTTTTTT-TATGGGATTTTAAAATGGAATAGATTAAATTAATTAATTTAA---TTGAGAACTT 1096
Al-Qassim clone            1013 TCGCCCCCTTTGGTTGATTTCTTTTTTTTTTATGGTATGGGATTTTAAAATGGAATATATTGAATTAATTAATTGAGAACTTTTTAATTT 1102
Madinah clone 1            921  TCGCCCCTTTTCTTTGATTTCTTTTTTTTTT----TATGGGATTTTAAAATAGAATAGATGACATTAATTAACTTAA---TTGAGAACTT 1003
Madinah clone 2            1225 TCGCCCCCTTTTTTTGGTTGATTTCTTTTTTTTT-TATGGGATTTTTTAATGGAATAGATTAAATCAATTAATTTAA---TTGAGAACTT 1310
Lemna japonica             1053 CCTTCCCGTTT--TCAGTT-----C---TTAGTA-TAATGTAATGCAAAATTTTTTAGAGTATGCGTTGTAACGCAA---TAAACAAGGT 1128
Lemna perpusilla           665  CCTTCCCGTTT--TTAGTT-----C---TTAGTA-TAATGTAATGGAAAACTTTTTTGAGTATGCGTTGTAACGCAA---CAAACAAGGT 740
Landoltia punctata         960  TCGCCCCCTTTGGTTGATT-----TC--TTTTTT-TTTTATGGTATGGGATTTTAAAA---------TGGAATATAT---TGAATTAATT 1029
Spirodela polyrhiza        1190 TCGCCCCCTTTTTTTGGTTGATTTCTTTTTTTTT-TATGGGATTTTTTAATGGAATAGATTAAATCAATTAATTTAA---TTGAGAACTT 1275
Lemna gibba                994  TCGCCCCCTTTGTTTGATT-----TCTTTTTTTT-TATGGGATTTTAAAATGGAATAGATTAAATTAATTAATTTAA---TTGAGAACTT 1074
Lemna aequinoctialis       993  TCGCCCCTTTTCTTTGATT---TCTTTTTTTTT-TATGGGATTTTAAAATAGAATAGATGACATTAATTAACTTAA---TTGAGAACTT 1075
Lemna minor                935  TCGCCCCCTTTGTTTGATT--TCTTTTTTTTTTT-TATGGGATTTAAAAATGGAATAGATTAAATTAATTAATTTAA---TTGAGAACTT 1018

                                       1450      1460      1470      1480      1490      1500      1510      1520      1530
                                  ....|....|....|....|....|....|....|....|....|....|....|....|....|....|....|....|....|....|
Riyadh clone               1353 T-TTTATT------TATTATTTAA---TTCTAA-------------------TTAAAGTTTACAATTACAAGAACATACTTATTGGGTTA 1413
Dhahran clone              1100 T-TTTATT------TATTATTTAA---TTCTAA-------------------TTAAAGTTTACAATTACAAGAACATACTTATTGGGTTA 1160
Tohama clone               1081 T-TTTATT------TATTATTTAA---TTCTAA-------------------TTAAAGTTTACAATTACAAGAACATACTTATTGGGTTA 1141
Al-Baha clone              659  A-TGTA------------GACAAA---AT--AC-------------------CATTCTATTAACGCAAGGAGTA----------ATACCA 701
Jazan clone                1072 T-TTTATT------TATTATTTTA---TTCTAA-------------------TTAAAGTTTACAATTACAAGAGCATACTTATTGGGTTA 1132
Al-Taif clone              1094 T-TTTATT------TATTATTTAA---TTCTAA-------------------TTAAAGTTTACAATTACAAGAACATACTTATTGGGTTA 1154
Tanomah clone              1097 T-TTTATT------TATTATTTAA---TTCTAA-------------------TTAAAGTTTACAATTACAAGAACATACTTATTGGGTTA 1157
Al-Qassim clone            1103 A-TTTATTATTTTATAATAATTAAATATTCTAAATTAAATATTCTAATTAAATTAAAGTTTACAATTACAAGAAGATACTTATTGGGTTA 1191
Madinah clone 1            1004 T-TTTATT------TATTATTTTA---TTCTAA-------------------TTAAAGTTTACAATTACAAGAGCATACTTATTGGGTTA 1064
Madinah clone 2            1311 T-TTAATT------TATAATTTTA---TTCTAA-------------------TTAAA-TTAAAGTTTACAAGAAGATACTTATTGGGTTA 1370
Lemna japonica             1129 A-TTTACC------AATTGACAAA---AT--AG-------------------CCAGGACCTAACCCAATAAGTATGTTCTTGTAATTGTA 1187
Lemna perpusilla           741  A-TTTA----------TTGACAAA---AT--AG-------------------TCAGGCCCTAACCCAATAAGTATGCTCTTGTAATTGTA 795
Landoltia punctata         1030 A-ATTGAG------AACTTTTTAA---TT-TAT-------------------TTATTATTTTA--TAATAATTAAATATTCTAAATTAAA 1087
Spirodela polyrhiza        1276 T-TTAATT------TATAATTTTA---TTCTAA-------------------TTAAA-TTAAAGTTTACAAGAAGATACTTATTGGGTTA 1335
Lemna gibba                1075 T-TTTATT------TATTATTTAA---TTCTAA-------------------TTAAAGTTTACAATTACAAGAACATACTTATTGGGTTA 1135
Lemna aequinoctialis       1076 T-TTTATT------TATTATTTTA---TTCTAA-------------------TTAAAGTTTACAATTACAAGAGCATACTTATTGGGTTA 1136
Lemna minor                1019 TATTTATT------TATTATTTAA---TTCTAA-------------------TTAAAGTTTACAATTACAAGAACATACTTATTGGGTTA 1080

                                       1540      1550      1560      1570      1580      1590      1600      1610      1620
                                  ....|....|....|....|....|....|....|....|....|....|....|....|....|....|....|....|....|....|
Riyadh clone               1414 GGTCCTGGCTA---TTTTGTCAATTGGTA-AATACCTTGTTTATTGCGTTACAACGCATACTCTAAAAAATTTTGCATTACATTATACTA 1499
Dhahran clone              1161 GGTCCTGGCTA---TTTTGTCAATTGGTA-AATACCTTGTTTATTGCGTTACAACGCATACTCTAAAAAATTTTGCATTACATTATACTA 1246
Tohama clone               1142 GGTCCTGGCTA---TTTTGTCAATTGGTA-AATACCTTGTTTATTGCGTTACAACGCATACTCTAAAAAATTTTGCATTACATTATACTA 1227
Al-Baha clone              702  AACCTC---------------------TA-AAAAAG---------AGGTTTGGTA-------TTA-------TTCCGTCATCAAGCCCTA 746
Jazan clone                1133 GGGCCTGACTA-------TTTTGTCAATA-AATACCTTGTTTGTTGCGTTAGAACGCATACTCAAAAAAGTTTTCCCTTACATTATACTA 1214
Al-Taif clone              1155 GGTCCTGGCTA---TTTTGTCAATTGGTA-AATACCTTGTTTATTGCGTTACAACGCATACTCTAAAAAATTTTGCATTACATTATACTA 1240
```

```
Tanomah clone          1158 GGTCCTGGCTA---TTTTGTCAATTGGTA-AATACCTTGTTTATTGCGTTACAACGCATACTCTAAAAAATTTTGCATTACATTATACTA 1243
Al-Qassim clone        1192 GGTCCTGGGTA---TTTTGTCAATTGATA-AATACCTTGTTTGTTGCGTTGCAACGCATACTAAAAAAGGTTTCCATTA-----TACTA 1272
Madinah clone 1        1065 GGGCCTGACTA---TTTTGTCAA----TA-AATACCTTGTTTGTTGCGTTAGAACGCATACTCAAAAAAGTTTTCCCTTACATTATACTA 1146
Madinah clone 2        1371 GGTCCTGGGTA---TTTTGTCAATTGATA-AATACCTTGTTTGTTGCGTTGCAACGCATACAA-AAAAGGTTTCCGTTACATTATACTA 1455
Lemna japonica         1188 AACTTTAATTAGAATTAAATA-ATAAATA-AAAAAGTTCTCAATTAAATTAATTAATTTAATCTA-------TTCCATTTTAAAATCCCA 1268
Lemna perpusilla       796  AACTTTAATTAGAATAAAATA-ATAAATA-AAAAAGTTCTCAATTAAGTTAATTAATGTCATCTA-------TTCCATTTTAAAATCCCA 876
Landoltia punctata     1088 TATTCTAATTAAATTAAAGTTTACAATTACAAGAAGATACTTATTGGGTTAGGTC-------CTG--------GGTATTTTGTCAATTGA 1162
Spirodela polyrhiza    1336 GGTCCTGGGTA---TTTTGTCAATTGATA-AATACCTTGTTTGTTGCGTTGCAACGCATACAAAAAAGGTTTC-CGTTACATTATACTA 1420
Lemna gibba            1136 GGTCCTGGCTA--TTTTGTCA-ATTGGTA-AATACCTTGTTTATTGCGTTACAACGCATACTCTAAAAAATTTTGCATTACATTATACTA 1221
Lemna aequinoctialis   1137 GGGCCTGACTA---TTTTGTCAA----TA-AATACCTTGTTTGTTGCGTTAGAACGCATACTCAAAAAAGTTTTCCCTTACATTATACTA 1218
Lemna minor            1081 GGTCCTGGCTA---TTTTGTCAATTGGTA-AATACCTTGTTTATTGCGTTACAACGCATACTCAAAAAAGTTTTGCATTACATTATACTA 1166

                                   1630      1640      1650      1660      1670      1680      1690      1700      1710
                            ....|....|....|....|....|....|....|....|....|....|....|....|....|....|....|....|....|....|
Riyadh clone           1500 AGAACTGAAAACGGGAAGGAAGAAAGCGAGAGGATCTGCTAATTACTAATCCTAAAATCAGTCCTTCCCGGAGGTATTCTCTCAACGAAT 1589
Dhahran clone          1247 AGAACTGAAAACGGGAAGGAAGAAAGCGAGAGGATCTGCTAATTACTAATCCTAAAATCAGTCCTTCCCGGAGGTATTCTCTCAACGAAT 1336
Tohama clone           1228 AGAACTGAAAACGGGAAGGAAGAAAGCGAGAGGATCTGCTAATTACTAATCCTAAAATCAGTCCTTCCCGGAGGTATTCTCTCAACGAAT 1317
Al-Baha clone          747  TACAC---------------------------------------TAAGACAAAAAG-----------------------TCT------ 766
Jazan clone            1215 AGAACTAAAAACGGGAAGGAAGAAAGCGAGAGGATCTGCTAATTACTAATCCTAAAATCAGTCCTTCCCGGAGGTATTCTCTCAACGAAT 1304
Al-Taif clone          1241 AGAACTGAAAACGGGAAGGAAGAAAGCGAGAGGATCTGCTAATTACTAATCCTAAAATCAGTCCTTCCCGGAGGTATTCTCTCAACGAAT 1330
Tanomah clone          1244 AGAACTGAAAACGGGAAGGAAGAAAGCGAGAGGATCTGCTAATTACTAATCCTAAAATCAGTCCTTCCCGGAGGTATTCTCTCAACGAAT 1333
Al-Qassim clone        1273 AGAACTAAAAACGGGAAGGAAGAAAGCGAGAGGATCTCCTAATTACTAATCCTAAAATCAGTCCTTCCCGGAGGTATTCTATCAACTAAT 1362
Madinah clone 1        1147 AGAACTAAAAACGGGAAGGAAGAAAGCGAGAGGATCTGCTAATTACTAATCCTAAAATCAGTCCTTCCCGGAGGTATTCTCTCAACGAAT 1236
Madinah clone 2        1456 AGAACTAAAAACGGGAAGGAAGAAAGCGAGAGGATCTGCTAATTACTAATCCTAAAATCAGTCCTTCCCGGAGGTATTCTCTCAACGAAT 1545
Lemna japonica         1269 TAAAAAAAAAGAA---ATCAAACAAAGGGGGCGA----------AGTAATACAAAAAGAACAGAACTCTGTTCTTTTTTAGTCC------ 1339
Lemna perpusilla       877  TAAAAAAAAAAAATAAATCAAAGAAAAGGGGCGA----------AGTAATACAAAAAG-----AACTCTGTTCTTTTTTAGTCC------ 945
Landoltia punctata     1163 TAAATACCTTGTTTGTTGCGTTGCAACGCA----------------TACTAAAAAAAG-----------GTTTCCATTATACTAA----- 1220
Spirodela polyrhiza    1421 AGAACTAAAAACGGGAAGGAAGAAAGCGAGAGGATCTGCTAATTACTAATCCTAAAATCAGTCCTTCCCGGAGGTATTCTCTCAACGAAT 1510
Lemna gibba            1222 AGAACTGAAAACGGGAAGGAAGAAAGCGAGAGGATCTGCTAATTACTAATCCTAAAATCAGTCCTTCCCGGAGGTATTCTCTCAACGAAT 1311
Lemna aequinoctialis   1219 AGAACTAAAAACGGGAAGGAAGAAAGCGAGAGGATCTGCTAATTACTAATCCTAAAATCAGTCCTTCCCGGAGGTATTCTCTCAACGAAT 1308
Lemna minor            1167 AGAACTGAAAACGGGAAGGAAGAAAGCGAGAGGATCTGCTAATTACTAATCCTAAAATCAGTCCTTCCCGGAGGTATTCTCTCAACGAAT 1256

                                   1720      1730      1740      1750      1760      1770      1780      1790      1800
                            ....|....|....|....|....|....|....|....|....|....|....|....|....|....|....|....|....|....|
Riyadh clone           1590 AAGTAATTGTTAGAGTACAATGTTGATATAATTCGAAGAAGCAAAAAGCAAGTCTAAGTCAAAAAG--TCTATTACGTACTTTTTTTGAT 1677
Dhahran clone          1337 AAGTAATTGTTAGAGTACAATGTTGATATAATTCGAAGAAGCAAAAAGCAAGTCTAAGTCAAAAA--GTCTATTACGTACTTTTTTTGAT 1424
Tohama clone           1318 AAGTAATTGTTAGAGTACAATGTTGATATAATTCGAAGAAGCAAAAAGCAAGTCTAAGTCAAAAA--GTCTATTACGTACTTTTTTTGAT 1405
Al-Baha clone          766  ---TATCCA----------------ATGGTA------GATGGAGCT------------------------------------------- 787
Jazan clone            1305 AAGTAATTGTTAGAGTGCAATGTTGATAGAATTCGAAGAAGCAAAAAGCAAGTCTAAGTCAAAAAG-------TACTTTCTTTT-----T 1382
Al-Taif clone          1331 AAGTAATTGTTAGAGTACAATGTTGATATAATTCGAAGAAGCAAAAAGCAAGTCTAAGTCAAAAAG--TCTATTACGTACTTTTTTTGAT 1418
Tanomah clone          1334 AAGTAATTGTTAGAGTACAATGTTGATATAATTCGAAGAAGCAAAAAGCAAGTCTAAGTCAAAAAG--TCTATTACGTACTTT------- 1414
Al-Qassim clone        1363 AAGTAATTGTTAGAGTGCAATGTTGATATAATTCGAAGAAGCAGAAAACAAGTCTAAGTAAAAAAA-GTCTATTATGTACTTTTTTC--T 1449
Madinah clone 1        1237 AAGTAATTGTTAGAGTGCAATGTTGATAGAATTCGAAGAAGCAAAAAGCAAGTCTAAGTCAAAAA-----------GTACTTTCTTTT-T 1314
Madinah clone 2        1546 AAGTAATTGTTAGAGTGCAATGTTGATATAATTCGAAGAAACAAAAAGCAAGTCTAAGTCAAAAAAAATTACGTACGTACTTTTATTTCT 1635
Lemna japonica         1339 ---TATCTATAAGAGGAGAGTAT--ATGCAA------AATGTAACTGATTCTTTTGTTTCCTTGGG--CCACTGGCCATCCGCCGGTGGT 1416
Lemna perpusilla       945  ---TATCTATAAGAGGAAAGTAT--ATGCAA------AATGTAACTGATTCTTTTGTTTCCTTGGG--CCACTGGCCGTCTGCCGGGGGT 1022
Landoltia punctata     1220 ---GAACTAAAAACGGAAGGAAGAAAGCGA-------GAGGATCTCCTAATTACTAATCCTAAAA--TCAGTC-CTTCCCGGAGGTATT 1297
Spirodela polyrhiza    1511 AAGTAATTGTTAGAGTGCAATGTTGATATAATTCGAAGAAACAAAAAGCAAGTCTAAGTCAAAAAAAATTACGTACGTACTTTTATTTCT 1600
Lemna gibba            1312 AAGTAATTGTTAGAGTACAATGTTGATATAATTCGAAGAAGCAAAAAGCAAGTCTAAGTCAAAAAG--TCTATTACGTACTTTTTTTGAT 1399
Lemna aequinoctialis   1309 AAGTAATTGTTAGAGTGCAATGTTGATAGAATTCGAAGAAGCAAAAAGCAAGTCTAAGTCAAAAA-----------GTACTTTC-TTTTT 1386
Lemna minor            1257 AAGTAATTGTTAGAGTACAATGTTGATATAATTCGAAGAAGCAAAAAGCAAGTCTAAGTCAAAAAG--TCTATTACGTACTTT--TTTAT 1342

                                   1810      1820      1830      1840      1850      1860      1870      1880      1890
                            ....|....|....|....|....|....|....|....|....|....|....|....|....|....|....|....|....|....|
Riyadh clone           1678 TCTAGAATTAAA-----CAAATGGATTCGCAAATAAAAGTGCTAATGCCACAACCAGTC------------------------------- 1731
Dhahran clone          1425 TCTAGAATTA-----AACAAATGGATTCGCAAATAAAAGTGCTAATGCCACAACCAGTCCATAAATTGTTAAAGCTTCCATAAAAGC-AT 1508
Tohama clone           1406 TCTAGAATTA-----AACAAATGGATTCGCAAATAAAAGTGCTAATGCCACAACCAGTC------------------------------- 1459
Al-Baha clone          787  -------------------------TCAACTGCAGCT----------------------------------------------------- 799
Jazan clone            1383 TGTAGAATTA-----AACAAATGGATTCGCAAATAAAAGTGCTAATGCCACAACCAGTCCATAAATTGACA-----------------AT 1450
Al-Taif clone          1419 TCTAGAATTA-----AACAAATGGATTCGCAAATAAA----------------------------------------------------- 1450
Tanomah clone          1414 -------------------GGTGGATTT--AAACAAC----------------------------------------------------- 1430
Al-Qassim clone        1450 TATAGAATTA-----AACAAATGGATTCGCAAATAAAAGTGCTAATGCCACGACCAGTC------------------------------- 1503
Madinah clone 1        1315 TGTAGAATTA-----AACAAATGGATTCGCAAATAAAAGTGCTAATGCCACAACCAGTC------------------------------- 1368
Madinah clone 2        1636 TCTAAAATTAAATTAAACAAATGGATTCGCAAATAAAAGTGCTAATGCCACAACCAGTC------------------------------- 1694
Lemna japonica         1417 TTTGGGTTTA----ATACCGATATTTTAGCAACAAATCCAATAAATCTAAGCGTAGTACTTGGTGTAGTAATTTATTTTGGAAAGGGAAT 1502
Lemna perpusilla       1023 TTTGGGTTTA----ATACCGATATTTTAGCAACAAAT-----------------------------------------------------AT 1057
Landoltia punctata     1298 CTATCAACTA----ATAAGTAATTGTTAGAGTGCAATGTTGATA-------------------------TAATTCGAAGAAGCAGAAAAC 1358
Spirodela polyrhiza    1601 TCTAAAATTAAATTAAACAAATGGATTCGCAAATAAAAGTGCTAATGCCACAACCAGTCCATAAATTGTTAAAGCTTCCATAAAAGC-AT 1689
Lemna gibba            1400 TCTAGAATTA----A-ACAAATGGATTCGCAAATAAAAGTGCTAATGCCACAACCAGTCCATAAATTGTTAAAGCTTCCATAAAAGC-AT 1483
Lemna aequinoctialis   1387 TGTAGAATTAAA-----CAAATGGATTCGCAAATAAAAGTGCTAATGCCACAACCAGTC------------------------------- 1440
Lemna minor            1343 TCTAGAATT-----AAACAAATGGATTCGCAAATAAAAGTGCTAATGCCACAACCAGTCCATAAATTGTTAAA--------------AT 1412

                                   1900      1910      1920      1930      1940      1950      1960      1970      1980
                            ....|....|....|....|....|....|....|....|....|....|....|....|....|....|....|....|....|....|
Riyadh clone           1731 --------------------CATAAATTGTTAAAGCTTCCATAAAAGCTGTAATAATGAGAAAGGTTTCTATATATTCTACCAAATCGAT 1801
Dhahran clone          1509 GGAAGAATTCAAAGGATATTTACAAAAAGGTGGATTTAAACAACAACACTTCCTATATCCACTTCTCTTTCAAGAGTATATTTATGG-AC 1597
Tohama clone           1459 ----------------TATTTACAAAAAGGTGGATTTAAACAACAACACTTCCTATATCCACTTCTCTTTCAAGAGTATATTTATGG-AC 1532
Al-Baha clone          799  ---------------AAGTCTAGAGGGAAGT---------------------TGTGAGCATTACT---TCAAGAGTATATTTATGG-AC 848
Jazan clone            1451 GGAAGAATTCAAAGGATATTTACAAAAAGGTGGATTTAAACAACAACACTTCCTATATCCACTTCTCTTTCAAGAGTATATTTATGC-AC 1539
Al-Taif clone          1450 --------------AGTGCTAATGCCACAACCAGTCCATAAA--------TTGTTGAACTGCTGTAAATTCAAGAGTATATTTATGG-AC 1517
Tanomah clone          1430 --------------AACACT--TCCTATATCCA-------------------------CTTCTCT---TTCAAGAGTATATTTATGG-AC 1475
Al-Qassim clone        1503 ------------------------------------------------------------------------------------------ 1503
Madinah clone 1        1368 --------------------CACGAATATCATAACGGGAATAATAGCATTACTCCAA--------------ATAAATCTACTTATGG-TT 1423
Madinah clone 2        1694 --------------------CATAAATTGTT----------------------------------------------------------- 1705
Lemna japonica         1503 GGAAAAATTCAAAGGATATTTACAAAAGGGTGGATTTAAACAACAACACTTCCTATATCCACTTCTCTTTCAAGAGTATATTTATGC-AC 1591
Lemna perpusilla       1058 GGAAGAATTCAAAGGATATTTACAAAAAGGTGGATTTAAACAACAACACTTCCTATATCCACTTCTCTTTCAAGAGTATATTTATGC-AC 1146
Landoltia punctata     1359 AAGTCTAAGTAAAAAAAGTCTATTATGTACT---TTTTTCTTATAGAATTAAACAAATGGATTCGCAAATAAAAGTGCTAATGCCACGAC 1445
Spirodela polyrhiza    1690 GGAAGAATTCAAAGGATATTTACAAAAAGGTGGATTTAAACAACAACACTTCCTATATCCACTTCTCTTTCAAGAGTATATTTACGT-AC 1778
```

```
Lemna gibba             1484 GGAAGAATTCAAAGGATATTTACAAAAAGGTGGATTTAAACAACAACACTTCCTATATCCACTTCTCTTTCAAGAGTATATTTATGG-AC 1572
Lemna aequinoctialis    1440 --------------------CATAAATTGTTAAAGCTTCCATAAAAGCATGTTCCCACTTTACATTTATTACGATTGCTTTTTCACGAAT 1510
Lemna minor             1413 GGAAGAATTCAAAGGATATTTACAAAAAGGTGGATTTAAACAACAACACTTCCTATATCCACTTCTCTTTCAAGAGTATATTTATGC-AC 1501

                                     1990      2000      2010      2020      2030      2040      2050      2060      2070
                             ....|....|....|....|....|....|....|....|....|....|....|....|....|....|....|....|....|....|
Riyadh clone            1802 CAATAATATCAGAATCTGACAATTCTGCCCAAGCTGGCTTGCTAATAGGATTCCCCGATAGATTACAAAATTTGGCTTTTGCCAATGACC 1891
Dhahran clone           1598 TTGCTCACGA----TCAGGGTTTAAATGTAAATGCATCAACTTTTAACGAACCCCCTGAAATTTCAGGTTATGGCAATAAATATAGCTCA 1683
Tohama clone            1533 TTGCTCACGA----TCAGGGTTTAAATGTAAATGCATCAACTTTTAACGAACCCCCTGAAATTTCAGGTTATGGCAATAAATATAGCTCA 1618
Al-Baha clone           849  TTGCTCACGA----TCAGGGTTTAAATGTAAATGCATCAACTTTTAACGAACCCCCTGAAATTTCAGGTTATGGCAATAAATATAGCTCA 934
Jazan clone             1540 TTGCTCATGA----TCAGGGTTTAAATGTAAATGCATCAACTTTTACTGAACCCCCTGAAATTTCAGGTTATCACAATAAATATAGCTCA 1625
Al-Taif clone           1518 TTGCTCACGA----TCAGGGTTTAAATGTAAATGCATCAACTTTTAACGAACCCCCTGAAATTTCAGGTTATGGCAATAAATATAGCTCA 1603
Tanomah clone           1476 TTGCTCACGA----TCAGGGTTTAAATGTAAATGCATCAACTTTTAACGAACCCCCTGAAATTTCAGGTTATGGCAATAAATATAGCTCA 1561
Al-Qassim clone         1503 ----------------------------------CATAAATTGTTAA--AGCTTCC--------------------ATAAA--------- 1528
Madinah clone 1         1424 TTTCAAAAGA-----------TAATCCAAGACTCTATAGGTTCCTATATAATTCTTATGTAGTTGA----ATGCGAATCCATATTTCTTT 1498
Madinah clone 2         1705 ------------------------------------------------------------------------------------------ 1705
Lemna japonica          1592 TTGCTCACGA----TCAGGGTTTAAATGTAAATGCATCAACTTTTAACGAACCCCCTGAAATCTCAGGTTATGGCAAAAAATATAGCTCA 1677
Lemna perpusilla        1147 TTGCTCATGA----TCAGGGTTTAAATGTAAATGCATCAACTTTTACTGAACCCCCTGAAATTTCAGGTTATCACAATAAATATAGCTCA 1232
Landoltia punctata      1446 CAGTCCATAAATTGTTAAAGCTTCCATAAAAGCGCATCAACTTTTAATGAACCCGCTGAAATTTCCGGTTATGACAATAAATATAGCTCA 1535
Spirodela polyrhiza     1779 TTGCTCATGA----TCATGGTTTAAATGTAAATGCATCAACTTTTAATGAACCCTCGGAAATTTCAGGTTATGACAATAAATAATCTAGCTTA 1864
Lemna gibba             1573 TTGCTCACGA----TCAGGGTTTAAATGTAAATGCATCAACTTTTAACGAACCCCCTGAAATTTCAGGTTATGGCAATAAATATAGCTCA 1658
Lemna aequinoctialis    1511 ATCATAACGGGAATAATAGCATTACTCCAAATAAATCTACTTATGGTTTTTCAAAAGATAATCCAAGACTCTATAGGTTCCTATATAATT 1600
Lemna minor             1502 TTGCTCACGA----TCAGGGTTTAAATGTAAATGCATCAACTTTTAACGAACCCCCTGAAATTTCAGGTTATGGCAATAAATATAGCTCA 1587

                                     2080      2090      2100      2110      2120      2130      2140      2150      2160
                             ....|....|....|....|....|....|....|....|....|....|....|....|....|....|....|....|....|....|
Riyadh clone            1892 CAATCATAGGAATAATTGAATATCATTGTTTCAAATTTTGGAGTAAAGGTATCTATTAAAAAGGCATTCTCTAGCATTTGACTCTTCGCGG 1981
Dhahran clone           1684 TTACTTGTGAAACGGTTAATTATTCGAATATACCAACAGAATAGTTTTCTCTATTCTGTCAATAATTCTAAGCAAAATAGATTCGTAGGA 1773
Tohama clone            1619 TTACTTGTGAAACGGTTAATTATTCGAATATACCAACAGAATAGTTTTCTCTATTCTGTCAATAATTCTAAGCAAAATAGATTCGTAGGA 1708
Al-Baha clone           935  TTACTTGTGAAACGGTTAATTATTCGAATATACCAACAGAATAGTTTTCTCTATTCTGTCAATAATTCTAAGCAAAATAGATTCGTAGGA 1024
Jazan clone             1626 TTACTTGTGAAACGGTTAATTAAGCGAATATACCAACAGAATCGTTTTATCTATTCTGTTACTAATTCTAAGCAAAATAGATTTGTTGGA 1715
Al-Taif clone           1604 TTACTTGTGAAACGGTTAATTATTCGAATATACCAACAGAATAGTTTTCTCTATTCTGTCAATAATTCTAAGCAAAATAGATTCGTAGGA 1693
Tanomah clone           1562 TTACTTGTGAAACGGTTAATTATTCGAATATACCAACAGAATAGTTTTCTCTATTCTGTCAATAATTCTAAGCAAAATAGATTCGTAGGA 1651
Al-Qassim clone         1528 ----------AGCGGTTAATTACTCGAATATACCAACAGAATAGTTTGATCCATTCTGTTAATGATTCTAAGCAAAATAGATTCATTGGA 1608
Madinah clone 1         1499 TTCTTC-----GTAAATCATC-CTCTTATTTACGATCAACATCTTTTGGACCCCTTCTTGAGCGAACACA-GTTCTATGGAAAAATGAAA 1581
Madinah clone 2         1705 ------------------------------------------------------------------------------------------ 1705
Lemna japonica          1678 TTACTTGTGAAACGGTTAATTACTCGAATATACCAACAGAATAGTTTTATCTATTCTGTCAATAATTCTAAGCAAAATAGATTCGTTGGA 1767
Lemna perpusilla        1233 TTACTTGTAAAACGGTTAATTAAGCGAATATACGAACAGAATAGTTTTATCTATTCTGTTAATAATTCTAAGCAAAATAGATTTGTTGGA 1322
Landoltia punctata      1536 TTACTTGTGAAACGGTTAATTACTCGAATATACCAACAGAATAGTTTGATCCATTCTGTTAATGATTCTAAGCAAAATAGATTCATTGGA 1625
Spirodela polyrhiza     1865 TTACTTGTGAAACGTTAATAACTCGAATATACCAACAGAATAGTTTGATCCATTCTGTTAATGATTCTAACCAAAATATATTCGTTGGA 1954
Lemna gibba             1659 TTACTTGTGAAACGGTTAATTATTCGAATATACCAACAGAATAGTTTTCTCTATTCTGTCAATAATTCTAAGCAAAATAGATTCGTAGGA 1748
Lemna aequinoctialis    1601 CTTATGTAGTTGAATGCGAATCCATATTTCTTTTTCTTCGTAAATCATCCTCTTATTTACGATCAACATCTTTTGGACCCCTTCTTGAGC 1690
Lemna minor             1588 TTACTTGTGAAACGGTTAATTACTCGAATATACCAACAGAATAGTTTTATCTATTCTGTCAATAATTCTAAGCAAAATAGATTCGTTGGA 1677

                                     2170      2180      2190      2200      2210      2220      2230      2240      2250
                             ....|....|....|....|....|....|....|....|....|....|....|....|....|....|....|....|....|....|
Riyadh clone            1982 ATGAAAGATTTATTGGCACACTTGAAAGATAACCCAAAAAATAGAAAGAAAAATGGGGGAATTGATTTATGTGTATCCTACTGGGTTGAG 2071
Dhahran clone           1774 CACAATAAGAATTATTATTATAAAATGATATGTGAGGGTTTTTCTATTGTTGTAGAAATTCCCTTTTCACTGCGATTAGTATCTTCTATC 1863
Tohama clone            1709 CACAATAAGAATTATTATTATAAAATGATATGTGAGGGTTTTTCTATTGTTGTAGAAATTCCCTTTTCACTGCGATTAGTATCTTCTATC 1798
Al-Baha clone           1025 CACAATAAGAATTATTATTATAAAATGATATGTGAGGGTTTTTCTATTGTTGTAGAAATTCCCTTTTCACTGCGATTAGTATCTTCTATC 1114
Jazan clone             1716 CACAACAATCATTTTTATTCTCAAATGATATCAGAGGGTTTTTCTAGTGTTGTCGAAATTCCGTTTTCACCGCGCTTAGTATCTTCTCTC 1805
Al-Taif clone           1694 CACAATAAGAATTATTATTATAAAATGATATGTGAGGGTTTTTCTATTGTTGTAGAAATTCCCTTTTCACTGCGATTAGTATCTTCTATC 1783
Tanomah clone           1652 CACAATAAGAATTATTATTATAAAATGATATGTGAGGGTTTTTCTATTGTTGTAGAAATTCCCTTTTCACTGCGATTAGTATCTTCTATC 1741
Al-Qassim clone         1609 CACAACAAGAATTTTTATTATCAAATGATATCCGAGGGTTTTGCTATCGTAGTAGAAATTCCGTTTTCAATGCGATTAGTATCTTCTCTC 1698
Madinah clone 1         1582 CATATTGGAGTAACTTGTTGT--AATGATTTCAGAAAACCCTATGTTTATTCAAGGATCC---TTTCA-TGC-ATTA-TGTTAGGTATC 1663
Madinah clone 2         1705 ------------------------------------------------------------------------------------------ 1705
Lemna japonica          1768 CACAATAAGACTTTTTATTATAAAATGATATCAGAGGGTTTTGCTATTGTTGTAGAAATTCCGTTTTCACTGCGATTAGTATCTTCTCGC 1857
Lemna perpusilla        1323 CACAACAATCATTTTTATTCTCAAATGATATCAGAGGGTTTTTCTAGTGTTGTCGAAATTCCGTTTTCACCGCGCTTAGTATCTTCTCTC 1412
Landoltia punctata      1626 CACAACAAGAATTTTTATTATCAAATGATATCCGAGGGTTTTGCTATCGTAGTAGAAATTCCGTTTTCAATGCGATTAGTATCTTCTCTC 1715
Spirodela polyrhiza     1955 CACAACAAGAATTTTTATTATCAAATGATATCAGAGGGTTTTGCTATCGTTGTGGAAATTCCTTTTTCGCTGCGATTAGTATCTTCTTTC 2044
Lemna gibba             1749 CACAATAAGAATTATTATTATAAAATGATATGTGAGGGTTTTTCTATTGTTGTAGAAATTCCCTTTTCACTGCGATTAGTATCTTCTATC 1838
Lemna aequinoctialis    1691 GAACACAGTTCTATGGAAAAATGAAACATATTGGAGTAACTTGTTGTAATGATTTTCAGAAAACCCTATGTTTATTCAAGGATCCTTTCA 1780
Lemna minor             1678 CACAATAAGAATTTTTATTATAAAATGATATCAGAGGGTTTTGCTATTGTTGTAGAAATTCCGTTTTCACTGCGATTAGTATCTTCTCTC 1767

                                     2260      2270      2280      2290      2300      2310      2320      2330      2340
                             ....|....|....|....|....|....|....|....|....|....|....|....|....|....|....|....|....|....|
Riyadh clone            2072 ACCAAAAGTGAAAATGACATTGCCATAAATTCACAAA-GTAAGATTTCCATTTCTTCATCAGAAGATGAGTCCCTTTTGAAGCTATAATG 2160
Dhahran clone           1864 AAAGAAACAAAAGAAATAATGAAATTTCAGAATTTAC-GATCTATTCATTCACTATTTCCTTTTTTAGAGGATAAATTTTCACATTTAAA 1952
Tohama clone            1799 AAAGAAACAAAAGAAATAATGAAATTTCAGAATTTAC-GATCTATTCATTCACTATTTCCTTTTTTAGAGGATAAATTTTCACATTTAAA 1887
Al-Baha clone           1115 AAAGAAACAAAAGAAATAATGAAATTTCAGAATTTAC-GATCTATTCATTCACTATTTCCTTTTTTAGAGGATAAATTTTCACATTTAAA 1203
Jazan clone             1806 AAAAAAAAAAAAGAAATACCGAAATATCAGAATTTAC-GATCTATTCATTCACTATTTCCATTTTTAGAGGATAAATTTACACATTTAAC 1894
Al-Taif clone           1784 AAAGAAACAAAAGAAATAATGAAATTTCAGAATTTAC-GATCTATTCATTCACTATTTCCTTTTTTAGAGGATAAATTTTCACATTTAAA 1872
Tanomah clone           1742 AAAGAAACAAAAGAAATAATGAAATTTCAGAATTTAC-GATCTATTCATTCACTATTTCCTTTTTTAGAGGATAAATTTTCACATTTAAA 1830
Al-Qassim clone         1699 AAAAAA---AAAGAAATACCGAAATATCAGAATTTAC-GATCTATTCATTCAATATTCCCATTTTTAGAGGATAAATTTGCACATTTAAA 1784
Madinah clone 1         1664 AAGGAA------AATCCATTATGGCTTCAAAAGGGACTGATTTCCTGATGAAAAAATGGAAATCTTACTTTGTGAATTT--ATGGCAATA 1745
Madinah clone 2         1705 ------------------------------------------------------------------------------------------ 1705
Lemna japonica          1858 AAAGAAAAAAAAGAAATAACGAAATCTCAGAATTTAC-GATCTATTCATTCACTATTTCCATTTTTAGAGGATCAATTTTCACATTTAAA 1946
Lemna perpusilla        1413 AAAAAAAAAAAAGAAATACCGAAATATCAGAATTTAC-GATCTATTCATTCACTATTTCCATTTTTAGAGGATAAATTTACACATTTAAA 1501
Landoltia punctata      1716 AAAAAAA---AAGAAATACCGAAATATCAGAATTTAC-GATCTATTCATTCAATATTCCCATTTTTAGAGGATAAATTTGCACATTTAAA 1801
Spirodela polyrhiza     2045 AAAGAAAAAAAAGAAATAACGAAATCTCAGAATTTAC-GATCTATTCATTCACTATTTCCATTTTTAGAGGATAAATTTGCACATTTAAA 2133
Lemna gibba             1839 AAAGAAACAAAAGAAATAATGAAATTTCAGAATTTAC-GATCTATTCATTCACTATTTCCTTTTTTAGAGGATAAATTTTCACATTTAAA 1927
Lemna aequinoctialis    1781 TGCATTATGTTAGGTATCAAGGAAAATCCATTATGGC-TTCAAAAGGGACTGATTTCCTGATGAAAAAATGGAAATCTTACTTTGTGAAT 1869
Lemna minor             1768 AAAGAAAAAAAAGAAATAACGAAATCTCAGAATTTAC-GATCTATTCATTCACTATTTCCATTTTTAGAGGATAAATTTTCACATTTAAA 1856
```

```
                               2350      2360      2370      2380      2390      2400      2410      2420      2430
                           ....|....|....|....|....|....|....|....|....|....|....|....|....|....|....|....|....|....|
Riyadh clone          2160 ------------CATTTTCCTTGATACCTAACATAATGCATGAAAGGATCCTTGAACAACCATAGGGTTTTCTGAAAATCATTACAACAA 2238
Dhahran clone         1953 TTCTGTATCAGATATAGTA-ATACCTTATCCAATCCATCTAGAAATATTGCTTCAAATTCTACAATGCTGGATACAAGATGTTC--CCAC 2039
Tohama clone          1888 TTCTGTATCAGATATAGTA-ATACCTTATCCAATCCATCTAGAAATATTGCTTCAAATTCTACAATGCTGGATACAAGATGTTC--CCAC 1974
Al-Baha clone         1204 TTCTGTATCAGATATAGTA-ATACCTTATCCAATCCATCTAGAAATATTGCTTCAAATTCTACAATGCTGGATACAAGATGTTC--CCAC 1290
Jazan clone           1895 TTATGTATCAGATATACTG-ATACCCTATCCGGTTCATCTAGAAATATTGGTTCAAATTCTACAATGCTGGATACAAGATGTTC--CCAC 1981
Al-Taif clone         1873 TTCTGTATCAGATATAGTA-ATACCTTATCCAATCCATCTAGAAATATTGCTTCAAATTCTACAATGCTGGATACAAGATGTTC--CCAC 1959
Tanomah clone         1831 TTCTGTATCAGATATAGTA-ATACCTTATCCAATCCATCTAGAAATATTGCTTCAAATTCTACAATGCTGGATACAAGATGTTC--CCAC 1917
Al-Qassim clone       1785 TTATGTATCAGATATACTG-ATACCTTATCCGGTTCATCTAGAAATATTGGTTCAAATTCTACAATGCTGGGTACAGGATGTTC--CCGC 1871
Madinah clone 1       1746 TCGT-TTCACTTTTGGTCTGAACCTAGTAGGATACACATAAACCAATTCCCCCATTTTTCTTTCTATTTTTTAGGTTATCTTT--CAAG 1832
Madinah clone 2       1705 ----------------------------------------------------------------------------------------- 1705
Lemna japonica        1947 TTATGTATCAGATATACTA-ATACCTTATCCGGTCCATCTAGAAATATTGGTTCAAATTCTACAATGCTGGATACAAGATGTTC--CCAC 2033
Lemna perpusilla      1502 TTATGTATCAGATATACTG-ATACCCTATCCGGTTCATCTAGAAATATTGGTTCAAATTCTACAATGCTGGATACAAGATGTTC--CCAC 1588
Landoltia punctata    1802 TTATGTATCAGATATACTG-ATACCTTATCCGGTTCATCTAGAAATATTGGTTCAAATTCTACAATGCTGGGTACAGGATGTTC--CCGC 1888
Spirodela polyrhiza   2134 TTATGTATCAGATATACTA-ATACCTTATCCGTCCATCTAGAAATATTGGTTCAAATTCTACAATGCTGGATACAAGATGTTC--CCTC 2220
Lemna gibba           1928 TTCTGTATCAGATATAGTA-ATACCTTATCCAATCCATCTAGAAATATTGCTTCAAATTCTACAATGCTGGATACAAGATGTTC--CCAC 2014
Lemna aequinoctialis  1870 TTATGGCAATATCGTTTTCACTTTTGGTCTGAACCTAGTAGGATACACATAAACCAATTCCCCCATTTTTCTTTTCTATTTTTTAGGTTAT 1959
Lemna minor           1857 TTATGTATCAGATATACTA-ATACCTTATCCGGTCCATCTAGAAATATTGGTTCAAATTCTACAATGCTGGATACAAGATCTTC--CCAC 1943

                               2440      2450      2460      2470      2480      2490      2500      2510      2520
                           ....|....|....|....|....|....|....|....|....|....|....|....|....|....|....|....|....|....|
Riyadh clone          2239 GTTACTCCAATATGTTTCATTTTTCCATAGAAGTGTGTTCGCTCAAGAAGGGGTC---------------------------------GA 2295
Dhahran clone         2040 TTTACATTTATTACGATTGATTTTTTACGACTATCACAATCGGAGTAATAGCATT-ACTCCAAATAAATCTAGTTATGGTTTTTCAAAAG 2128
Tohama clone          1975 TTTACATTTATTACGATTGATTTTTTACGACTATCACAATCGGAGTAATAGCATT-ACTCCAAATAAATCTAGTTATGGTTTTTCAAAAG 2063
Al-Baha clone         1291 TTTACATTTATTACGATTGATTTTTTACGACTATCACAATCGGAGTAATAGCATT-ACTCCAAATAAATCTAGTTATGGTTTTTCAAAAG 1379
Jazan clone           1982 TTTACATTTATTACGATTGCTTTTTCACGAATATCATAACGGGAATAATAGCATT-ACTCCAAATAAATCTACTTATGGTTTTTCAAAAG 2070
Al-Taif clone         1960 TTTACATTTATTACGATTGATTTTTTACGACTATCACAATCGGAGTAATAGCATT-ACTCCAAATAAATCTAGTTATGGTTTTTCAAAAG 2048
Tanomah clone         1918 TTTACATTTATTACGATTGATTTTTTACGACTATCACAATCGGAGTAATAGCATT-ACTCCAAATAAATCTAGTTATGGTTTTTCAAAAG 2006
Al-Qassim clone       1872 TTTACATTTATTACGATTGCTTTTTCATGACTATCATAATGGGAGTAATTGCATT-ACTCCAAAAAAATCTAGTTATGGTTTTTCAAAAG 1960
Madinah clone 1       1833 TGTACCAATA----AATCTTTCATACGCGAAGAGTCAAATGCTAGAGAATTCGTT-TTTAATAGAAACCTTTACTCAAAAATTTGAAACG 1917
Madinah clone 2       1705 ---------------------TTTTTATGG---------------GAGGGGGTC---------------------------------AA 1726
Lemna japonica        2034 TTTACATTTATTACGATTGATTTTTCACGACTATAACAATGGGAGTAATAGCATT-ACTCCAAATAAATCTAGTTATGGTTTTTCAAAAG 2122
Lemna perpusilla      1589 TTTACATTTATTACGATTGCTTTTTCACGAATATCATAACGGGAATAATAGCATT-ACTCCAAATAAATCTACTTATGGTTTTTCAAAAG 1677
Landoltia punctata    1889 TTTACATTTATTACGATTGCTTTTTCATGACTATCATAATGGGAGTAATTGCATT-ACTCCAAAAAAATCTAGTTATGGTTTTTCAAAAG 1977
Spirodela polyrhiza   2221 TTTACATTTATTACGATTCCTTTTTCACCAATATCACAATGGGAATAATAGCATT-ACTCCAAAGAAATCTAGTTATGGTTTTTCAAAAG 2309
Lemna gibba           2015 TTTACATTTATTACGATTGATTTTTTACGACTATCACAATCGGAGTAATAGCATT-ACTCCAAATAAATCTAGTTATGGTTTTTCAAAAG 2103
Lemna aequinoctialis  1960 CTTTCAAGTGTACCAATAAATCTTTCATACGCGAAGAGTCAAATGCTAGAGAATTCGTTTTTAATAGAAACCTTTACTCAAAAATTTGAA 2049
Lemna minor           1944 TTTACATTTATTACGATTGATTTTTCACGACTATCACAATGGGAGTAATAGCATT-CCTCCAAATAAATCTAGTTTTGGTTTTTCAAAAG 2032

                               2530      2540      2550      2560      2570      2580      2590      2600      2610
                           ....|....|....|....|....|....|....|....|....|....|....|....|....|....|....|....|....|....|
Riyadh clone          2296 AAAGAT------GTTGATCGTAAATAAGAGGATGATTTACGAAGAAATAAAA---AATGGATTCACATTCGA----------------- 2359
Dhahran clone         2129 ATAATCCAAGACTCTATAGGTTCCTATATAATTCTTATGTAGTCGAATGTGAATCCATTTTTATTTTCTTCGTAAATCATCCTCTTATT 2218
Tohama clone          2064 ATAATCCAAGACTCTATAGGTTCCTATATAATTCTTATGTAGTCGAATGTGAATCCATTTTTATTTTCTTCGTAAATCATCCTCTTATT 2153
Al-Baha clone         1380 ATAATCCAAGACTCTATAGGTTCCTATATAATTCTTATGTAGTCGAATGTGAATCCATTTTTATTTTCTTCGTAAATCATCCTCTTATT 1469
Jazan clone           2071 ATAATCCAAGACTCTATAGGTTCCTATATAATTCTTATGTAGTTGAATGCGAATCCATATTTCTTTTCTTCGTAAATCATCCTCTTATT 2160
Al-Taif clone         2049 ATAATCCAAGACTCTATAGGTTCCTATATAATTCTTATGTAGTCGAATGTGAATCCATTTTTATTTTCTTCGTAAATCATCCTCTTATT 2138
Tanomah clone         2007 ATAATCCAAGACTCTATAGGTTCCTATATAATTCTTATGTAGTCGAATGTGAATCCATTTTTATTTTCTTCGTAAATCATCCTCTTATT 2096
Al-Qassim clone       1961 ATAATCCAAGACTCTATAGGTTCCTATATAATTCTTATGTAGTCGAATGCGAATCCATATTTGTTTTCTTCGTAAATCATCCTCTTATT 2050
Madinah clone 1       1918 ATGATA------TCCATTATTCC---TATGATTGGATCATTGGCAAAAGCTAA---ATTTTGTAATCTATCGGGGAATCCTATTAGCAAG 1995
Madinah clone 2       1727 GCAG------------------------------------------------------------------------------------- 1730
Lemna japonica        2123 ATAATCCAAGACTCTATAGGTTCCTATATAATTCTTATGTAGTCGAATGCGAATCCATATTTTATTTTCTTCGTAAATCATCCTCTTATT 2212
Lemna perpusilla      1678 ATAATCCAAGACTCTATAGGTTCCTATATAATTCTTATGTAGTTGAATGCGAATCCATATTTCTTTATCTTCGTAAATCATCCTCTTATT 1767
Landoltia punctata    1978 ATAATCCAAGACTCTATAGGTTCCTATATAATTCTTATGTAGTCGAATGCGAATCCATATTTGTTTTCTTCGTAAATCATCCTCTTATT 2067
Spirodela polyrhiza   2310 ATAATCAAGACTATATAGGTTCCTATATAATTCTTATGTAGTAGAATACGAATCCATATTTGTTTTCTCCGTAAATCATCCTCTTATT 2399
Lemna gibba           2104 ATAATCCAAGACTCTATAGGTTCCTATATAATTCTTATGTAGTCGAATGTGAATCCATTTTTATTTTCTTCGTAAATCATCCTCTTATT 2193
Lemna aequinoctialis  2050 ACGATG------ATATCCATTATTCCTATGATTGGATCATTGGCAAAAGCTAA---ATTTTGTAATCTATCGGGGAATCCTATTAGCAAG 2130
Lemna minor           2033 ATAATCCAAGACTCTATAGGTTCCTATATAATTCTTATGTAGTCGAATGCGAATCCATATTTGATTTCTTCGTAAATCATCCTCTTATT 2122

                               2620      2630      2640      2650      2660      2670      2680      2690      2700
                           ....|....|....|....|....|....|....|....|....|....|....|....|....|....|....|....|....|....|
Riyadh clone          2359 -CTACATAAGAATTATATAGGAACCTATAGAGTCTTGGATTATCTTTTGAAAAACCATAACTAGATTTATTTGG----AGTAATGCTATT 2444
Dhahran clone         2219 TACGATCAAC---ATCTTTTCGACCCCTTCTTGAGCGAACACACTTCTATGGAAAAATGAAACATATTGGAGTAACTTGTTGTAATGATT 2305
Tohama clone          2154 TACGATCAAC---ATCTTTTCGACCCCTTCTTGAGCGAACACACTTCTATGGAAAAATGAAACATATTGGAGTAACTTGTTGTAATGATT 2240
Al-Baha clone         1470 TACGATCAAC---ATCTTTTCGACCCCTTCTTGAGCGAACACACTTCTATGGAAAAATGAAACATATTGGAGTAACTTGTTGTAATGATT 1556
Jazan clone           2161 TACGATCAAG---ATCTTTTGGACCCCTTCTTGAGCGAACACAGTTCTATGGAAAAATGAAACATATTGGAGTAACTTGTTGTAATGATT 2247
Al-Taif clone         2139 TACGATCAAC---ATCTTTTCGACCCCTTCTTGAGCGAACACACTTCTATGGAAAAATGAAACATATTGGAGTAACTTGTTGTAATGATT 2225
Tanomah clone         2097 TACGATCAAC---ATCTTTTCGACCCCTTCTTGAGCGAACACACTTCTATGGAAAAATGAAACATATTGGAGTAACTTGTTGTAATGATT 2183
Al-Qassim clone       2051 TACGATCAAC---ATCTTTTGGATCCCTTCTTGAGCGAACACACTTCTATGGAAAAATGAAACATATTGGAGTAACTTGTTGTAATGATT 2137
Madinah clone 1       1996 CCAGCTTGGGCTGATTTGTCAGATTCGGATATTATTGATCGATTTGGTAGAATATATAGAAACCTTTCTCATTA--TTACAGTGG-GATC 2081
Madinah clone 2       1730 ----------------------------------------------------------------------------------------- 1730
Lemna japonica        2213 TACGATCAAC---ATCTTTTGGACCCCTTCTTGAGCGAACACACTTCTATGGAAAAATGAAACATATTGGAGTAACTTCTTGTAATGATT 2299
Lemna perpusilla      1768 TACGATCAAC---ATCTTTTGGACCCCTTCTTGAGCGAACACACTTCTATGGAAAAATGAAACATATAGGAGTAACTTGTTGTAATGATT 1854
Landoltia punctata    2068 TACGATCAAC---ATCTTTTGGATCCCTTCTTGAGCGAACACACTTCTATGGAAAAATGAAACATATTGGAGTAACTTGTTGTAATGATT 2154
Spirodela polyrhiza   2400 TACGATCAAC---ATCTTTTGGACCCTTCTTGAGCGAACATACTTCTATGGAAAAATGAAAAATATTGGAGTAACTCATTCTAATAATT 2486
Lemna gibba           2194 TACGATCAAC---ATCTTTTCGACCCCTTCTTGAGCGAACACACTTCTATGGAAAAATGAAACATATTGGAGTAACTTGTTGTAATGATT 2280
Lemna aequinoctialis  2131 CCAGCTTGGGCTGATTTGTCAGATTCGGATATTATTGATCGATTTGGTAGAATATATAGAAACCTTTCTCATTA----TTACAGTGGATC 2216
Lemna minor           2123 TACGATCAAC---ATCTTTTGGACCCCTTCTTGAGCGAACACACTTCTATGGAAAAATGAAACATATTGGAGTAACTTGTTGTAATGATT 2209
```

```
                              2710      2720      2730      2740      2750      2760      2770      2780      2790
                          ....|....|....|....|....|....|....|....|....|....|....|....|....|....|....|....|....|....|
Riyadh clone         2445 ACTCCGATTGTGATAGTCGTAAAAAATCAATCGTAATAAATGTAAAGTGGAACATCTTGTA----------------------------- 2506
Dhahran clone        2306 TTCAGAAAACCCTATGGT-TGTTCAAGGATCCTTTCATGCATTATGTTAGGTATCA---------------------------------- 2360
Tohama clone         2241 TTCAGAAAACCCTATGGT-TGTTCAAGGATCCTTTCATGCATTATGTTAGGTA------------------------------------- 2292
Al-Baha clone        1557 TTCAGAAAACCCTATGGT-TGTTCAAGGATCCTTTCATGCATTATGTTAGGTATCAAGGAAAATGCATTATAGCTTCAAAAGGGACTCAT 1645
Jazan clone          2248 TTCATAAAAGCCTATGTT-TATTCAAGGATCCTTTCATGCATTATG----------AGGCGG-------------TCAGCAGTTCCCCTT 2313
Al-Taif clone        2226 TTCAGAAAACCCTATGGT-TGTTCAAGGATCCTTTCATGCAT-------------------------------------CAGTTCCCCTT 2277
Tanomah clone        2184 TTCAGAAAACCCTATGGT-TGTTCAAGGATCCTTTCATGCATTATGTTAGGTATCAAGGAAAATGCATTATA----CAGCAGTTCCCCTT 2268
Al-Qassim clone      2138 TTCAGAAAACCCTATGGT-TGTTCAAGGATCCTTTCATGCATTATGTTAGGTATCAAGGAAAATCCATTATGGCTTCAAAAGGGACTCAT 2226
Madinah clone 1      2082 TTCAAAAAAACAAACTTTGTATCGAATAAAGTATATACTTAGGCTTTCATGTGCTAGAA------------------------------- 2140
Madinah clone 2      1730 ------------------------------------------------------------------------------------------ 1730
Lemna japonica       2300 TTCAGAAAACCCTATGGT-TATTCAAGGATCCTTTCATGCATTATGTTAGGTATCAAGGAAAATGCATTATGGCTTCAAAAGGGACTCAT 2388
Lemna perpusilla     1855 TTCAGAAAACCGTATGTT-TATTCAAGGATCCTTTCATGCATTATGTTAGGTATCAAGGAAAATCCATTATGGCTTCAAAAGGGACTCAT 1943
Landoltia punctata   2155 TTCAGAAAACCCTATGGT-TGTTCAAGGATCCTTTCATGCATTATGTTAGGTATCAAGGAAAATCCATTATGGCTTCAAAAGGGACTCAT 2243
Spirodela polyrhiza  2487 TTCAGAAAACCCTATGGT-TGTTCAAGGATCCTTTTATGCATTATGTTAGGTATCAAGGAAAATCAATTATGGCTTCAAAAGGGACTCAT 2575
Lemna gibba          2281 TTCAGAAAACCCTATGGT-TGTTCAAGGATCCTTTCATGCATTATGTTAGGTATCAAGGAAAATGCATTATAGCTTCAAAAGGGACTCAT 2369
Lemna aequinoctialis 2217 TTCAAAAAAACAAACTTTGTATCGAATAAAGTATATACTTAGGCTTTCATGTGCTAGAACTT---------------------------- 2278
Lemna minor          2210 TTCAGAAAACCCTATGGT-TGTTCAAGGATCCTTTCATGCATTATGTTAGGTATCAAGGAAAATGCATTATGGCTTCAAAAGGGACTCAT 2298

                              2800      2810      2820      2830      2840      2850      2860      2870      2880
                          ....|....|....|....|....|....|....|....|....|....|....|....|....|....|....|....|....|....|
Riyadh clone         2506 -----------------------------------------------------------------------------------TCCAGCA 2513
Dhahran clone        2360 ------------------------------------------------------------------------------TTTTTATGCAGC 2372
Tohama clone         2292 ------------------------------------------------------------------------------------------ 2292
Al-Baha clone        1646 CTTCTGATGAAGAAATGGAAAT---------------------------------------------------------------TGA-- 1670
Jazan clone          2314 TCTCGGTCCGAGAAGTGTA----------------------------------------------------------------------- 2332
Al-Taif clone        2278 TCTCGGTCCGAGAAGTGTA----------------------------------------------------------------------- 2296
Tanomah clone        2269 TCTCGGTCCGAGAAGTGTA----------------------------------------------------------------------- 2287
Al-Qassim clone      2227 CTTCTGATGAAGAAATGGAAATCTTACTTTGTGAATTTATGGCAATGTCATTTTCACTTTTGGTCTCAACCCAGTATTTTTGTTGGAGGG 2316
Madinah clone 1      2140 -----------------------------------------------------------------------------------CTTTGGC 2147
Madinah clone 2      1730 ------------------------------------------------------------------------------------------ 1730
Lemna japonica       2389 CTTCTGATGAAGAAATGGAAATCTTACT------------------------------------------------------TTGTGAAT 2424
Lemna perpusilla     1944 TTCCTGATGAAAAAATGGAAATCTTACT------------------------------------------------------TTGTAAAT 1979
Landoltia punctata   2244 CTTCTGATGAAGAAATGGAAATCTTACT------------------------------------------------------TTGTGAAT 2279
Spirodela polyrhiza  2576 CTTCTGATGAAGAAATGGAAATCTTACT------------------------------------------------------TTGTGAAT 2611
Lemna gibba          2370 CTTCTGATGAAGAAATGGAAATCTTACT------------------------------------------------------TTGTGAAT 2405
Lemna aequinoctialis 2278 -----------------------------------------------------------------------------------TGGCTCG 2285
Lemna minor          2299 CTTCTGATGAAGAAATGGAAATCTTACT------------------------------------------------------TTGTGAAT 2334

                              2890      2900      2910      2920      2930      2940      2950      2960      2970
                          ....|....|....|....|....|....|....|....|....|....|....|....|....|....|....|....|....|....|
Riyadh clone         2514 TTGTAGAATTTCCCCTTTC----TCGGTCCGAGAAGTGTATTGTAGGAACCGGGCTGGAACGCCAAGCGGCTTTAGATTCTG------GC 2593
Dhahran clone        2373 GTCAAGCAGTTCCCCTTTC----TCGGTCCGAGAAGTGTATTGTAGGAACCGGGCTGGAACGCCAAGCGGCTTTAGATTCTG------GC 2452
Tohama clone         2292 ------CAGTTCCCCTTTC----TCGGTCCGAGAAGTGTATTGTAGGAACCGGGCTGGAACGCCAAGCGGCTTTAGATTCTG------GC 2366
Al-Baha clone        1670 -----GCAGTTCCCCTTTC----TCGGTCCGAGAAGTGTATTGTAGGAACCGGGCTGGAACGCCAAGCGGCTTTAGATTCTG------GC 1745
Jazan clone          2332 ------------------------TTGTAGGAACCGGACTGGA---ACGCCAAGCGG-------------CTTTAGATTCTG------GC 2376
Al-Taif clone        2296 ------------------------TTGTAGGAACCGGGCTGGA---ACGCCAAGCGG-------------CTTTAGATTCTG------GC 2340
Tanomah clone        2287 ------------------------TTGTAGGAACCGGGCTGGA---ACGCCAAGCGG-------------CTTTAGATTCTG------GC 2331
Al-Qassim clone      2317 GTCAAGCAGTTCCGCTTTC----TCGGTCCGAGAAGTGCATTGTAGGAACCGGATTGGAACGCCAAGCGGCTTTAGATTCTG------GA 2396
Madinah clone 1      2148 TCGTAACAGTTCCCCTTTC----TCGGTCCGAGAAGTGTATTGTAGGAACCGGACTGGAACGCCAAGCGGCTTTAGATTCTG------GC 2227
Madinah clone 2      1730 ---------TTCCCCTTTC----TCGGTCCGAGAAGTGCATTGTAGGAACCGGACTGGAACGCCAAGCGGCTTTAGATTCTG------GA 1801
Lemna japonica       2425 TTATGGCAATGTCATTTTCACTTTTGGTCTCAACCCAGTAGGATACACATAAACCAATTCCCCCATTTTTCTTTCTATTTTTTGGGTTAT 2514
Lemna perpusilla     1980 TTATGGCAATATCGTTTTCACTTTTGGTCTGAACCTAGTAGGATACACATAAACCAATTCCCCCATTTTTCTTTCTATTTTTTAGGTTAT 2069
Landoltia punctata   2280 TTATGGCAATGTCATTTTCACTTTTGGTCTCAACCCAGTAGGATACACATAAACCAATTCCCCCATTTTTCTTTCTATTTTTTGGGTTAT 2369
Spirodela polyrhiza  2612 TTATGGCAATGTCATTTTCACTTTTGGTCTCAACCCAGTAGGATCCACATAAATCAATTCTCCCATTTTTCTTTCTATTTTTTGGGTTAT 2701
Lemna gibba          2406 TTATGGCAATGTCATTTTCACTTTTGGTCTCAACCCAGTAGGATACACATAAATCAATTCCCCCATTTTTCTTTCTATTTTTTGGGTTAT 2495
Lemna aequinoctialis 2286 TAACAGCAGTTCCCCTTTC----TCGGTCCGAGAAGTGTATTGTAGGAACCGGACTGGAACGCCAAGCGGCTTTAGATTCTG------GC 2365
Lemna minor          2335 TTATGGCAATGTCATTTTCACTTTTGGTCTCAACCCAGTAGGATACACATAAACCAATTCCCCCATTTTTCTTTCTATTTATTGGGTTAT 2424

                              2980      2990      3000      3010      3020      3030      3040      3050      3060
                          ....|....|....|....|....|....|....|....|....|....|....|....|....|....|....|....|....|....|
Riyadh clone         2594 GTTTCAGTTATAGC-----------CGAACACGAGG------------GAAAAGTCATTTCTACTGA-------TACTCACCAAATTGT- 2652
Dhahran clone        2453 GTTTCAGTTATAGC-----------CGAACACGAGG------------GAAAAGTCATTTCTACTGA-------TACTCACCAAATTGT- 2511
Tohama clone         2367 GTTTCAGTTATAGC-----------CGAACACGAGG------------GAAAAGTCATTTCTACTGA-------TACTCACCAAATTGT- 2425
Al-Baha clone        1746 GTTTCAGTTATAGC-----------CGAACACGAGG------------GAAAAGTCATTTCTACTGA-------TACTCACCAAATTGT- 1804
Jazan clone          2377 GTTTCAGTTATAGC-----------CGAACACGAGG------------GAAAAGTCATTTCTACTGA-------TACTCACCAAATTGT- 2435
Al-Taif clone        2341 GTTTCAGTTATAGC-----------CGAACACGAGG------------GAAAAGTCATTTCTACTGA-------TACTCACCAAATTGT- 2399
Tanomah clone        2332 GTTTCAGTTATAGC-----------CGAACACGAGG------------GAAAAGTCATTTCTACTGA-------TACTCACCAAATTGT- 2390
Al-Qassim clone      2397 GTTTCAGTTATAGC-----------CGAACACGAGG------------GAAAAGTCATTTCTACTGA-------TACTCACCGAATTGT- 2455
Madinah clone 1      2228 GTTTCAGTTATAGC-----------CGAACACGAGG------------GAAAAGTCATTTCTACTGA-------TACTCACCAAATTGT- 2286
Madinah clone 2      1802 GTTTCAGTTATAGC-----------CGAACACGAGG------------GAAAGGTCATTTCTACTGA-------TACTCACCAAATTGT- 1860
Lemna japonica       2515 CTTTCAAGTGTACCAATAAATCCTTCATCCGCGAAGAGTCAAATGCTAGAGAATTCCTTTTTAATAGATACCTTTACTCCAAAATTTGAA 2604
Lemna perpusilla     2070 CTTTCAAGTGTACCAATAAATCTTTCATACGCGAAGAGTCAAATTCTAGAGAATTCGTTTTTAATAGAAACCTTTACTCAAAAATTTGAA 2159
Landoltia punctata   2370 CTTTCAAGTGTACCAATAAATCCTTCATCCGTGAAGAGTCAAATGCTAGAGAATTCTTTTTTAATAGATACCGTTACTCCAAAATTTGAA 2459
Spirodela polyrhiza  2702 CTTTCAAGTGTACCTAGAAATCGATCATCGGTAACGACTCAAATGCTAGAGAATTCCTTTTTAATAGAGACCGTTACTAAAAAATTTGAA 2791
Lemna gibba          2496 CTTTCAAGTGTGCCAATAAATCTTTCATCCGCGAAGAGTCAAATGCTAGAGAATGCCTTTTTAATAGATACCTTTACTCCAAAATTTGAA 2585
Lemna aequinoctialis 2366 GTTTCAGTTATAGC-----------CGAACACGAGG------------GAAAAGTCATTTCTACTGA-------TACTCACCAAATTGT- 2424
Lemna minor          2425 CTTTCAAGTGTGCCAATAAATCCTTCATCCGCGAAGAGTCAAATGCTAGAGAATTCCTTTTTAATAGATAGCTTTACTCCAAAATTTGAA 2514
```

```
                                       3070      3080      3090      3100      3110      3120      3130      3140      3150
                              ....|....|....|....|....|....|....|....|....|....|....|....|....|....|....|....|....|....|
Riyadh clone             2652 -------------TTCTCAGGTAATGGAAACACT--------CTAAATATT-----CCATTAGTTA--TGTATCAACGTTCCAACAAAA 2714
Dhahran clone            2511 -------------TTCTCAGGTAATGGAAACACT--------CTAAATATT-----CCATTAGTTA--TGTATCAACGTTCCAACAAAA 2573
Tohama clone             2425 -------------TTCTCAGGTAATGGAAACACT--------CTAAATATT-----CCATTAGTTA--TGTATCAACGTTCCAACAAAA 2487
Al-Baha clone            1804 -------------TTCTCAGGTAATGGAAACACT--------CTAAATATT-----CCATTAGTTA--TGTATCAACGTTCCAACAAAA 1866
Jazan clone              2435 -------------TTCTCAAGTAATGGCAAGACT--------CTAAATATT-----CCATTAGTTA--TGTATCAACGTTCCAACAAAA 2497
Al-Taif clone            2399 -------------TTCTCAGGTAATGGAAACACT--------CTAAATATT-----CCATTAGTTA--TGTATCAACGTTCCAACAAAA 2461
Tanomah clone            2390 -------------TTCTCAGGTAATGGAAACACT--------CTAAATATT-----CCATTAGTTA--TGTATCAACGTTCCAACAAAA 2452
Al-Qassim clone          2455 -------------TTCTCAGGTAATGGAAACACT--------CTAAATATT-----CCATTAGTTA--TGTATCAACGTTCCAACAAAA 2517
Madinah clone 1          2286 -------------TTCTCAAGTAATGGCAAGACT--------CTAAATATT-----CCATTAGTTA--TGTATCAACGTTCCAACAAAA 2348
Madinah clone 2          1860 -------------TTCTCAGGTAATGGAAACACT--------GTAAATATT-----CCATTAGTTA--TGTATGAACGTTCCAACAAAA 1922
Lemna japonica           2605 ACAATGATATCAATTATTCCTATGATTGGGTCATTGGCAAAAGCTAAATTTTGTAATCTATCAGGGAATCCTATTAGCAAGCCAGCTTGG 2694
Lemna perpusilla         2160 ACGATGATATCCATTATTCCTATGATTGGATCATTGGCAAAAGCTAAATTTTGTAATCTATCGGGGAATCCTATTAGCAAGCCAGCTTGG 2249
Landoltia punctata       2460 ACGATGATATCAATTATTCCTATGATTGGGTCATTGGCAAAAGCTAAATTTTGTAATCTATCGGGGAATCCTATTAGCAAGCCAGTTTGG 2549
Spirodela polyrhiza      2792 ACGATGGTATCCATTATTCCTATGATTGGTTCATTGTCAAAAGCTAAATTTTGTAACCTATCGGGGAATCCGATTAGCAAGCCTGTTTGG 2881
Lemna gibba              2586 ACAATGATATCAATTATTCCTATGATTGGGTCATTGGCAAAAGCCAAATTTTGTAATCTATCGGGGAATCCTATTAGCAAGCCAGCTTGG 2675
Lemna aequinoctialis     2424 -------------TTCTCAAGTAATGGCAAGACT--------CTAAATATT-----CCATTAGTTA--TGTATCAACGTTCCAACAAAA 2486
Lemna minor              2515 ACAATGATATCAATTATTCCTATGATTGGGTCATTGGCAAAAGCTAAATTTTGTAATCTATCAGGGAATCCTATTAGCAAGCCAGCTTGG 2604
```

```
                                       3160      3170      3180      3190      3200      3210      3220      3230      3240
                              ....|....|....|....|....|....|....|....|....|....|....|....|....|....|....|....|....|....|
Riyadh clone             2715 AT--ACTTGTATGCACCAAAAACCTCAGGTTCAACGGGGTAAATACGTTAAAA-----------------------------AGGGACAA 2773
Dhahran clone            2574 AT--ACTTGTATGCACCAAAAACCTCAGGTTCAACGGGGTAAATACGTTAAAA-----------------------------AGGGACAA 2632
Tohama clone             2488 AT--ACTTGTATGCACCAAAAACCTCAGGTTCAACGGGGTAAATACGTTAAAA-----------------------------AGGGACAA 2546
Al-Baha clone            1867 AT--ACTTGTATGCACCAAAAACCTCAGGTTCAACGGGGTAAATACGTTAAAA-----------------------------AGGGACAA 1925
Jazan clone              2498 AT--ACTTGTATGCACCAAAAACCACAGGTTAAACGGGGTAAATACGTTAAAA-----------------------------AGGGACAA 2556
Al-Taif clone            2462 AT--ACTTGTATGCACCAAAAACCTCAAGTTCAACGGGGTAAATACGTTAAAA-----------------------------AGGGACAA 2520
Tanomah clone            2453 AT--ACTTGTATGCACCAAAAACCTCAGGTTCAACGGGGTAAATACGTTAAAA-----------------------------AGGGACAA 2511
Al-Qassim clone          2518 AT--ACTTGTATGCACCAAAAACCTCAGGTTCAACGTGGTAAATACGTTAAAA-----------------------------AGGGACAA 2576
Madinah clone 1          2349 AT--ACTTGTATGCACCAAAAACCACAGGTTAAACGGGGTAAATACGTTAAAA-----------------------------AGGGACAA 2407
Madinah clone 2          1923 AT--ACTTGTATGCACCAAAAACCTCAGGTTCAACGGGGTAAATACATTAAAA-----------------------------AGGGACAA 1981
Lemna japonica           2695 GCAGATTTGTCAGATTCTGATATTATTGATCGATTTGGTAGAATATATAGAAACCTTTCTCATTATTACAGTGGGTCTTCAAAAAAACAG 2784
Lemna perpusilla         2250 GCTGATTTGTCAGATTCGGATATTATTGATCGATTTGGTAGAATATATAGAAACCTTTCTCATTATTACAGTGGATCTTCAAAAAAACAA 2339
Landoltia punctata       2550 GCCGATTTGTCAGATTCTGATATTATTGATCGATTTGGTAGAATATGTAGAAACCTTTCTCATTATTACAGTGGGTCTTCAAAAAAACAG 2639
Spirodela polyrhiza      2882 GCCGATTTATCAGATCTGGATATTATTGATCGATTTGGTCGGATATGTAGAAACCTTTCTCATTATTACAGTGGGTCTTCAAAAAAACAG 2971
Lemna gibba              2676 GCAGAATTGTCAGATTCTGATATTATTGATCGATTTGGTAGAATATATAGAAACCTTTCTCATTATTACAGTGGGTCTTCAAAAAAACAG 2765
Lemna aequinoctialis     2487 AT--ACTTGTATGCACCAAAAACCACAGGTTAAACGGGGTAAATACGTTAAAA-----------------------------AGGGACAA 2545
Lemna minor              2605 GCAGATTTGTCAGATTCTGATATTATTGATCGATTTGGTAGAATATATAGAAACCTTTCTCATTATTACAGTGGGTCTTCAAAAAAACAG 2694
```

```
                                       3250      3260      3270      3280      3290      3300      3310      3320      3330
                              ....|....|....|....|....|....|....|....|....|....|....|....|....|....|....|....|....|....|
Riyadh clone             2774 ATTTT--AGCTGACGGGGC---------GGCTACCGTTGGTGGAGAACTCGCTTTGGGA----AAAAATGTATTAGTAGCTTATATGCCG 2848
Dhahran clone            2633 ATTTT--AGCTGACGGGGC---------GGCTACCGTTGGTGGAGAACTCGCTTTGGGA----AAAAATGTATTAGTAGCTTATATGCCG 2707
Tohama clone             2547 ATTTT--AGCTGACGGGGC---------GGCTACCGTTGGTGGAGAACTCGCTTTGGGA----AAAAATGTATTAGTAGCTTATATGCCG 2621
Al-Baha clone            1926 ATTTT--AGCTGACGGGGC---------GGCTACCGTTGGTGGAGAACTCGCTTTGGGA----AAAAATGTATTAGTAGCTTATATGCCG 2000
Jazan clone              2557 ATTTT--AGCTGACGGGGC---------GGCTACCGTTGGTGGAGAACTCGCTTTGGGA----AAAAATGTCTTAGTAGCTTATATGCCA 2631
Al-Taif clone            2521 ATTTT--AGCTGACGGGGC---------GGCTACCGTTGGTGGAGAACTCGCTTTGGGA----AAAAATGTATTAGTAGCTTATATGCCA 2595
Tanomah clone            2512 ATTTT--AGCTGACGGGGC---------GGCTACCGTTGGTGGAGAACTCGCTTTGGGA----AAAAATGTATTAGTAGCTTATATGCCG 2586
Al-Qassim clone          2577 ATTTT--GGCCGACGGGGC---------GGCTACCGTTGGTGGAGAACTCGCTTTGGGA----AAAAATGTATTAGTAGCTTATATGCCA 2651
Madinah clone 1          2408 ATTTT--AGCTGACGGGGC---------GGCTACCGTTGGTGGAGAACTCGCTTTGGGA----AAAAATGTCTTAGTAGCTTATATGCCA 2482
Madinah clone 2          1982 ATTTT--AGCCGACGGGGC---------GGCTACTGTTGGTGGAGAACTCGCTTTGGGA----AAAAATGTATTAGTAGCTTATATGCCA 2056
Lemna japonica           2785 AGTTTGTATCGAATAAAGTATATACTTAGACTTTCCTGTGCTCGAACTTTGGCTCGTAAACATAAAAGTACGGTACGCGCTTTTTTGCAA 2874
Lemna perpusilla         2340 ACTTTGTATCGAATAAAGTATATACTTAGGCTTTCATGTGCTCGAACTTTGGCTCGTAAACATAAAAGTACAGTACGCGCTTTTTTGCAA 2429
Landoltia punctata       2640 AGTTTATATCGAATAAAGTATATACTTAGACTTTCATGTGCTCGAACTTTGGCGCGTAAACATAAAAGTACAGTACGTGCTTTTTTGCAA 2729
Spirodela polyrhiza      2972 AGTTTGTATCGAATAAAGTATATACTTCGACTTTCATGTGCTCGAACTTTGGCGCGTAAGCATAAAAGTACTGTACGCGCTTTTTTGCAA 3061
Lemna gibba              2766 AGTTTGTATCGAATAAAATATATACTTAGACTTTCCTGTGCTCGAACTTTGGCTCGTAAACATAAAAGTACAGTACGCGCTTTTTTGCAA 2855
Lemna aequinoctialis     2546 ATTTT--AGCTGACGGGGC---------GGCTACCGTTGGTGGAGAACTCGCTTTGGGA----AAAAATGTCTTAGTAGCTTATATGCCA 2620
Lemna minor              2695 AGTTTGTATCGAATAAAATATATACTTAGACTTTCCTGTGCTCGAACTTTGGCTCGTAAACATAAAAGTACAGTACGCGCTTTTTTGCAA 2784
```

```
                                       3340      3350      3360      3370      3380      3390      3400      3410      3420
                              ....|....|....|....|....|....|....|....|....|....|....|....|....|....|....|....|....|....|
Riyadh clone             2849 TGGGAAGGCT-------------ACAATTTT--------GAAGATGCGGTACTT----------------------------------AT 2883
Dhahran clone            2708 TGGGAAGGCT-------------ACAATTTT--------GAAGATGCGGTACTT----------------------------------AT 2742
Tohama clone             2622 TGGGAAGGCT-------------ACAATTTT--------GAAGATGCGGTACTT----------------------------------AT 2656
Al-Baha clone            2001 TGGGAAGGCT-------------ACAATTTT--------GAAGATGCGGTACTT----------------------------------AT 2035
Jazan clone              2632 TGGGAAGGCT-------------ACAATTTT--------GAAGATGCGGTACTT----------------------------------AT 2666
Al-Taif clone            2596 TGGGAAGGCT-------------ACAATTTT--------GAAGATGCGGTACTT----------------------------------AT 2630
Tanomah clone            2587 TGGGAAGGCT-------------ACAATTTT--------GAAGATGCGGTACTT----------------------------------AT 2621
Al-Qassim clone          2652 TGGGAAGGCT-------------ACAATTTT--------GAAGATGCGGTACTT----------------------------------AT 2686
Madinah clone 1          2483 TGGGAAGGCT-------------ACAATTTT--------GAAGATGCGGTACTT----------------------------------AT 2517
Madinah clone 2          2057 TGGGAAGGCT-------------ACAATTTT--------GAAGATGCGGTACTT----------------------------------AT 2091
Lemna japonica           2875 AGATTAGGTTCAGAGTTTTTCGAAGAATTCTTTATGGAAGAAGAAAAAGTACTTTCTTTAATCTTACCAAGAACTTCTTATCCTTTACAT 2964
Lemna perpusilla         2430 AGATTAGGTTCAGAGTTTTTCGAAGAATTCTTTATGGAAGAAGAAAAAGTTCTTTCTTTAATCTTACCAAGAACTTGGTATTCTTCACAT 2519
Landoltia punctata       2730 AGATTAGGTTCAGAGTTTTTCGAAGAATTCTTTATGGAAGAAGAAAAAGTTCTTTCTTTAATTTTACCAAGAACTTCTTATCCTTTACAT 2819
Spirodela polyrhiza      3062 AGATTAGGTTCAGAGTTTTTAGAAGAATTCTTTATGGAAGAAGAAAAAGTTCTTTCTTTAATCTTACCAAGAACTTCTTATCCTTTACAC 3151
Lemna gibba              2856 AGATTAGGTTCAGAGTTTTTCGAAGAATTCTTTATGGAAGAAGAAAAAGTACTTTCTTTAATCTTACCAAGAACTTATTATCCTTTACAT 2945
Lemna aequinoctialis     2621 TGGGAAGGCT-------------ACAATTTT--------GAAGATGCGGTACTT----------------------------------AT 2655
Lemna minor              2785 AGATTAGGTTCAGAGTTTTTCGAAGAATTCTTTATGGAACAAGAAAAAGTGCTTTCTTTAATCTTACCAAGAACTTCTTATCCTTTACAT 2874
```

```
                                       3430      3440      3450      3460      3470      3480      3490      3500      3510
                              ....|....|....|....|....|....|....|....|....|....|....|....|....|....|....|....|....|....|
Riyadh clone             2884 TAGT------GAG----CGTTTGGTATATAGCGAT--------------------------ATTTAT----------------------- 2914
Dhahran clone            2743 TAGT------GAG----CGTTTGGTATATAGCGAT--------------------------ATTTATACTTCTTTTCACAT--------- 2787
Tohama clone             2657 TAGT------GAG----CGTTTGGTATATAGCGAT--------------------------ATTTATACTTCTTTTCACATCCG------ 2704
Al-Baha clone            2036 TAGT------GAG----CGTTTGGTATATAGCGAT--------------------------ATTTATACTT------------------- 2070
Jazan clone              2667 TAGT------GAG----CGTTTGGTATATAGCGAT--------------------------------ATTT------------------- 2695
Al-Taif clone            2631 TAGT------GAG----CGTTTGGTATATAGCGAT--------------------------ATTTGGGCTCCTT---------------- 2668
```

```
Tanomah clone             2622 TAGT------GAG----CGTTTGGTATATAGCGAT--------------------------ATTTATACTTCTT---------------- 2659
Al-Qassim clone           2687 TAGT------GAG----CGTTTGGTATATAGCGAT--------------------------ATTTATACTTCTTTTCACAAACC------ 2734
Madinah clone 1           2518 TAGT------GAG----CGTTTGGTATATAGCGAT--------------------------ATTTATACTTCTTTTCATCCA-------- 2563
Madinah clone 2           2092 TAGT------GAG----CGTTTGGTATATAGCGAT--------------------------ATTTATACTTCTTTC-------------- 2131
Lemna japonica            2965 CAGTTATCTAGAGAACCCGTTTGGTATTTGGATATTATTCGTATAAATGACTTGGTAAATCATTTCGATTTGTGAAAGTA---------- 3044
Lemna perpusilla          2520 CAGTTATCTAGAGAACCCGTTTGGTATTTGGATATTGTTCGTATAAATGACTTGGTAAATCATTTAGATTTATGAAAGT----------- 2598
Landoltia punctata        2820 CAGTTATATAGAGAACCTATTTGGTATTTGGATATTGTTCGTATAAATGACTTGGTGAATCATAAGCAGTTCCGCTTTCT---------- 2899
Spirodela polyrhiza       3152 AAGTTATATAGAGCACCCATTTGGTATTTGGATATTATTCGTATAAATGACTTGGTGAAT------AATTTATGAAAGCAGTTCCCCTTT 3235
Lemna gibba               2946 CAGTTATCTAGAGAACCCATTTGGTATTTGGATATTATTCGGATAAATGACTTGGTAAATCATTTCGATTTATGAAAGTA---------- 3025
Lemna aequinoctialis      2656 TAGT------GAG----CGTTTGGTATATAGCGAT--------------------------ATTTATACTTCTTTTCACATCCG------ 2703
Lemna minor               2875 CAGTTATCTAGAGAACCCATTTGGTATTTGGATATTATTCGTATAAATGACTTGGTAAATCATTTCGATTTATGAAAGCAGTTCCCCTTT 2964

                                        3520      3530      3540      3550      3560      3570      3580      3590      3600
                               ....|....|....|....|....|....|....|....|....|....|....|....|....|....|....|....|....|....|
Riyadh clone              2914 ------------------------------------------------------------------------------------------ 2914
Dhahran clone             2787 ------------------------------------------------------------------------------------------ 2787
Tohama clone              2704 --AAAACCACCACGTAGACATT-------------------------------------------------------------------- 2724
Al-Baha clone             2070 ------------------------------------------------------------------------------------------ 2070
Jazan clone               2695 ------------------------------------------------------------------------------------------ 2695
Al-Taif clone             2668 ------------------------------------------------------------------------------------------ 2668
Tanomah clone             2659 ------------------------------------------------------------------------------------------ 2659
Al-Qassim clone           2734 --CAA-------CCTAGACATT-------------------------------------------------------------------- 2747
Madinah clone 1           2563 ---AGTCCACCACGTAGACATT-------------------------------------------------------------------- 2582
Madinah clone 2           2131 ------------------------------------------------------------------------------------------ 2131
Lemna japonica            3044 --TAAAT--ATCGCTATATACCAAACGCTCACTAATAAGTACCGCATCTTCAAAATTGTAGCCTTCCCACGGCATATAAGCTACTAATAC 3130
Lemna perpusilla          2598 ------------GCTGGAT----------------------------------------------------------------------- 2605
Landoltia punctata        2899 --CGGTCCGAGAAGTGCATTGTAGGAACCGGATTGGAACGCCAAGCGGCTTTAGATTCTGGAGTTTCAGTTATAGCCGAACACGAGGGAA 2987
Spirodela polyrhiza       3236 CTCGGTCCGAGAAGTGCATTGTAGGAACCGGACTGGAACGCCAAGCGGCTTTAGATTCTGGAGTTTCAGTTATAGCCGAACACGAGGGAA 3325
Lemna gibba               3025 --TAAAT--ATCGCTATATACCAAACGCTCACTAATAAGTACCGCATCTTCAAAATTGTAGCCTTCCCATGGCATATAAGCTACTAATAC 3111
Lemna aequinoctialis      2703 ------------------------------------------------------------------------------------------ 2703
Lemna minor               2965 CTCGGTCCGAGAAGTGTATTGTAGGAACCGGGCTGGAACGCCAAGCGGCTTTAGATTCTGGCGTTTCAGTTATAGCCGAACACGAGGGAA 3054

                                        3610      3620      3630      3640      3650      3660      3670      3680      3690
                               ....|....|....|....|....|....|....|....|....|....|....|....|....|....|....|....|....|....|
Riyadh clone              2914 ------------------------------------------------------------------------------------------ 2914
Dhahran clone             2787 ------------------------------------------------------------------------------------------ 2787
Tohama clone              2724 ------------------------------------------------------------------------------------------ 2724
Al-Baha clone             2070 ------------------------------------------------------------------------------------------ 2070
Jazan clone               2695 ------------------------------------------------------------------------------------------ 2695
Al-Taif clone             2668 ------------------------------------------------------------------------------------------ 2668
Tanomah clone             2659 ------------------------------------------------------------------------------------------ 2659
Al-Qassim clone           2747 ------------------------------------------------------------------------------------------ 2747
Madinah clone 1           2582 ------------------------------------------------------------------------------------------ 2582
Madinah clone 2           2131 ------------------------------------------------------------------------------------------ 2131
Lemna japonica            3131 ATTTTTTCCCAAAGCGAGTTCTCCACCAACGGTAGCCGCCCCGTCAGCTAAAATTTGTCCCTTTTTAACGTATTTACCCCGTTGAACCTG 3220
Lemna perpusilla          2605 --------TCAAAGC--------------------------------------------------------------------------- 2612
Landoltia punctata        2988 AAGTCATTTCTACTGATACTCACCGAATTGTTTTCTCAGGTAATGGAAACACTCTAAATATTCCATTAGTTATGTATCAACGTTCCAACA 3077
Spirodela polyrhiza       3326 AGGTCATTTCTACTGATACTCACCAAATTGTTTTCTCAGGTAATGGAAACACTGTAAATATTCCATTAGTTATGTATGAACGTTCCAACA 3415
Lemna gibba               3112 ATTTTTTCCCAAAGCGAGTTCTCCACCAACGGTAGCCGCCCCGTCAGCTAAAATTTGTCCCTTTTTAACGTATTTACCCCGTTGAACTTG 3201
Lemna aequinoctialis      2703 ------------------------------------------------------------------------------------------ 2703
Lemna minor               3055 AAGTCATTTCTACTGATACTCACCAAATTGTTTTCTCAGGTAATGGAAACACTCTAAATATTCCATTAGTTATGTATCAACGTTCCAACA 3144

                                        3700      3710      3720      3730      3740      3750      3760      3770      3780
                               ....|....|....|....|....|....|....|....|....|....|....|....|....|....|....|....|....|....|
Riyadh clone              2914 ------------------------------------------------------------------------------------------ 2914
Dhahran clone             2787 ------------------------------------------------------------------------------------------ 2787
Tohama clone              2724 -----------------------------------------------------------CATAAACCGCTCTACCGT------------- 2742
Al-Baha clone             2070 ------------------------------------------------------------------------------------------ 2070
Jazan clone               2695 ------------------------------------------------------------------------------------------ 2695
Al-Taif clone             2668 ------------------------------------------------------------------------------------------ 2668
Tanomah clone             2659 ------------------------------------------------------------------------------------------ 2659
Al-Qassim clone           2747 -----------------------------------------------------------CATAAACCGCTCTACCGT------------- 2765
Madinah clone 1           2582 -----------------------------------------------------------CATAAACCGCTCTACCGT------------- 2600
Madinah clone 2           2131 ------------------------------------------------------------------------------------------ 2131
Lemna japonica            3221 AGGTTTTTGGTGCATACAAGTATTTTTGTTGGAACGTTGATACATAACTAATGGAATATTTAGAGTGTTTCCATTACCTGAGAAAACAAT 3310
Lemna perpusilla          2612 -------TGGTG-----------------------------------------------TTAAAG-------ATTAC------------- 2628
Landoltia punctata        3078 AAAATACTTGTATGCACCAAAAACCTCAGGTTCAACGTGGTAAATACGTTAAAAAGGGACAAATTTTGGCCGACGGGGCGGCTACCGTTG 3167
Spirodela polyrhiza       3416 AAAATACTTGTATGCACCAAAAACCTCAGGTTCAACGGGGTAAATACATTAAAAAGGGACAAATTTTAGCCGACGGGGCGGCTACTGTTG 3505
Lemna gibba               3202 AGGTTTTTGGTGCATACAAGTATTTTTGTTGGAACGTTGATACATAACTAATGGAATATTTAGAGTGTTTCCATTACCTGAGAAAACAAT 3291
Lemna aequinoctialis      2703 ------------------------------------------------------------------------------------------ 2703
Lemna minor               3145 AAAATACTTGTATGCACCAAAAACCTCAGGTTCAACGGGGTAAATACGTTAAAAAGGGACAAATTTTAGCTGACGGGGCGGCTACCGTTG 3234

                                        3790      3800      3810      3820      3830      3840      3850      3860      3870
                               ....|....|....|....|....|....|....|....|....|....|....|....|....|....|....|....|....|....|
Riyadh clone              2914 ------------------------------------------------------------------------------------------ 2914
Dhahran clone             2787 ------------------------------------------------------------------------------------------ 2787
Tohama clone              2742 ------------------------------------------------------------------------------------------ 2742
Al-Baha clone             2070 ------------------------------------------------------------------------------------------ 2070
Jazan clone               2695 ------------------------------------------------------------------------------------------ 2695
Al-Taif clone             2668 ------------------------------------------------------------------------------------------ 2668
Tanomah clone             2659 ------------------------------------------------------------------------------------------ 2659
Al-Qassim clone           2765 ------------------------------------------------------------------------------------------ 2765
Madinah clone 1           2600 ------------------------------------------------------------------------------------------ 2600
Madinah clone 2           2131 ------------------------------------------------------------------------------------------ 2131
Lemna japonica            3311 TTGGTGAGTATCAGTAGAAATGACTTTTCCCTCGTGTTCGGCTATAACTGAAACGCCAGAATCT-AAAGCCGCTTGGCGTTCCAGCCCGG 3399
Lemna perpusilla          2628 ------------------------------------------------------------------------------------------ 2628
Landoltia punctata        3168 GTGGAGAACTCGCTTTGGGAAAAAAATGTATTAGTAGCTTATATGCCATGGGAAGGCTACAATTTTGAAGATGCGGTACTTATTAGTGAGC 3257
Spirodela polyrhiza       3506 GTGGAGAACTCGCTTTGGGAAAAAAATGTATTAGTAGCTTATATGCCATGGGAAGGCTACAATTTTGAAGATGCGGTACTTATTAGTGAGC 3595
```

```
Lemna gibba             3292 TTGGTGAGTATCAGTAGAAATGACTTTTCCCTCGTGTTCGGCTATAACTGAAACGCCAGAATCT-AAAGCCGCTTGGCGTTCCAGCCCGG 3380
Lemna aequinoctialis    2703 ------------------------------------------------------------------------------------------ 2703
Lemna minor             3235 GTGGAGAACTCGCTTTGGGAAAAAATGTATTAGTAGCTTATATGCCGTGGGAAGGCTACAATTTTGAAGATGCGGTACTTATTAGTGAGC 3324

                                     3880      3890      3900      3910      3920      3930      3940      3950      3960
                             ....|....|....|....|....|....|....|....|....|....|....|....|....|....|....|....|....|....|
Riyadh clone            2914 ------------------------------------------------------------------------------------------ 2914
Dhahran clone           2787 ------------------------------------------------------------------------------------------ 2787
Tohama clone            2742 ------------------------------------------------------------------------------------------ 2742
Al-Baha clone           2070 ------------------------------------------------------------------------------------------ 2070
Jazan clone             2695 ------------------------------------------------------------------------------------------ 2695
Al-Taif clone           2668 ------------------------------------------------------------------------------------------ 2668
Tanomah clone           2659 ------------------------------------------------------------------------------------------ 2659
Al-Qassim clone         2765 ------------------------------------------------------------------------------------------ 2765
Madinah clone 1         2600 ------------------------------------------------------------------------------------------ 2600
Madinah clone 2         2131 ------------------------------------------------------------------------------------------ 2131
Lemna japonica          3400 TTCCTACAATACACTTCTCGGACCGAG--------------------------------------------------------------- 3426
Lemna perpusilla        2628 ------------------------------------------------------------------------------------------ 2628
Landoltia punctata      3258 GTTTGGTATATAGCGATATTTATACTTCTTTTCACATCCGAA----------------G----------TGCTGGATTCAAAGCTGGTGT 3321
Spirodela polyrhiza     3596 GTTTGGTATATAGCGATATTTATACTTCTTTCCACATCCGATGTCACCACAAACAGAGACTAAAGCAAGTGTTGGATTCAAAGCTGGTGT 3685
Lemna gibba             3381 TTCCTACAATACACTTCTCGGACCGAGAAGAAACTAAAGCAA----------------G----------TGCTGGATTCAAAGCTGGTGT 3444
Lemna aequinoctialis    2703 ------------------------------------------------------------------AAGTGCTGGATTCAAAGCTGGTGT 2727
Lemna minor             3325 GTTTGGTATATAGCGATATTTATACTTCTTTTCACATCCGAA------------------TAAAGCAAGTGCTGGATTCAAAGCTGGTGT 3396

                                     3970      3980      3990      4000      4010      4020      4030      4040      4050
                             ....|....|....|....|....|....|....|....|....|....|....|....|....|....|....|....|....|....|
Riyadh clone            2914 ----------------------TATACTCCTGAGTATGAGACAAAAGATACGGATATCTTGGCAGCATTCCGAGTAACTCCTCAACCTGG 2982
Dhahran clone           2787 ----------------------GATAATCCCAATTTTG-GTTTGATGGTAC--ATCCCAATAGAGGACGACCA-TACTTGTTCAATTTA- 2850
Tohama clone            2742 -----------AGTTTTTCGCGGATAATCCCAATTTTG-GTTTGATGGTAC--ATCCCAATAGAGGACGACCA-TACTTGTTCAATTTA- 2816
Al-Baha clone           2070 -----------------CTTTTCACATCC---------GAACAAAAGATACGGATATCTTGGCAGCATTCCGAGTAACTCCTCAACCTGG 2134
Jazan clone             2695 -----------------------------------------------------ATACTT------------------CTTTTCA------ 2708
Al-Taif clone           2668 -----------------TTCCATATACTCCTGAGTATGAGACAAAAGATACGGATATCTTGGCAGCATTCCGAGTAACTCCTCAACCTGG 2741
Tanomah clone           2659 -----------------TTC--------------------ACAAAAGATACGGATATCTTGGCAGCATTCCGAGTAACTCCTCAACCTGG 2712
Al-Qassim clone         2765 -----------AGTTTTTCGCGGATAATCCCAATTTTG-GTTTGATGGTAC--ATCCCAATAGAGGACGACCA-TACTTGTTCAATTTA- 2839
Madinah clone 1         2600 -----------AGTTTTTCGCGGATAATCCCAATTTTG-GTTTGATGGTAC--ATCCCAATAGAGGACGACCA-TACTTGTTCAATTTA- 2674
Madinah clone 2         2131 --------------------ATTATACTCCTGAGTATGAGACAAAAGATACGGATATCTTGGCAGCATTCCGAGTAACTCCTCAACCTGG 2201
Lemna japonica          3426 ----------AAATTGAATTATTATACTCCTGAGTATGAGACAAAAGATACGGATATCTTGGCAGCATTCCGAGTAACTCCTCAACCTGG 3506
Lemna perpusilla        2628 ----------AAATTGACTTATTATACTCCTGAGTATGAGACAAAAGATACGGATATCTTGGCAGCATTCCGAGTAACTCCTCAACCTGG 2708
Landoltia punctata      3322 TAAAGATTACAAATTGACTTATTATACTCCTGAGTATGAGACAAAAGATACGGATATCTTGGCAGCATTCCGAGTAACTCCTCAACCTGG 3411
Spirodela polyrhiza     3686 TAAAGATTACAAATTGACTTATTATACTCCTGAGTATGAGACAAAAGATACGGATATCTTGGCAGCATTCCGAGTAACTCCTCAACCTGG 3775
Lemna gibba             3445 TAAAGATTACAAATTGACTTATTATACTCCTGAGTATGAGACAAAAGATACGGATATCTTGGCAGCATTCCGAGTAACTCCTCAACCTGG 3534
Lemna aequinoctialis    2728 TAAAGATTACAAATTGACTTATTATACTCCTGAGTATGAGACAAAAGATACGGATATCTTGGCAGCATTCCGAGTAACTCCTCAACCTGG 2817
Lemna minor             3397 TAAAGATTACAAATTGACTTATTATACTCCTGAGTATGAGACAAAAGATACGGATATCTTGGCAGCATTCCGAGTAACTCCTCAACCTGG 3486

                                     4060      4070      4080      4090      4100      4110      4120      4130      4140
                             ....|....|....|....|....|....|....|....|....|....|....|....|....|....|....|....|....|....|
Riyadh clone            2983 AGTTCCACCTGAAGAAGCAGGGGCTGCAGTAGCTGCCGAATCTTCTACTGGTACATGGACAACTGTGTGGACTGATGGACTTACCAGCCT 3072
Dhahran clone           2850 ---TCTCTCTCAACTTGGATCCCATGAGGTGGGCCTTGGAAAGTTT--TGGAATACGCA-----GGAGGAATTCGCAAATCTTCCAAACG 2930
Tohama clone            2816 ---TCTCTCTCAACTTGGATCCCATGAGGTGGGCCTTGGAAAGTTT--TGGAATACGCA-----GGAGGAATTCGCAAATCTTCCAAACG 2896
Al-Baha clone           2135 AGTTCCACCTGAAGAAGCAGGGGCTGCAGTAGCTGCCGAATCTTCTACTGGTACATGGACAACTGTGTGGACTGATGGACTTACCAGCCT 2224
Jazan clone             2708 --------CTGAAGAAGCAGGTGCTGCAGTAGCTGCCGAATCTTCTACTGGTACATGGACAACTGTGTGGACTGATGGACTTACCAGCCT 2790
Al-Taif clone           2742 AGTTCCACCTGAAGAAGCAGGGGCTGCAGTAGCTGCCGAATCTTCTACTGGTACATGGACAACTGTGTGGACTGATGGACTTACCAGCCT 2831
Tanomah clone           2713 AGTTCCACCTGAAGAAGCAGGGGCTGCAGTAGCTGCCGAATCTTCTACTGGTACATGGACAACTGTGTGGACTGATGGACTTACCAGCCT 2802
Al-Qassim clone         2839 ---TCTCTCTCAACTTGGATCCCATGAGGTGGGCCTAGGAAAGTTT--TGGAATAAGCA-----GGAGGAATTCGCAAATCTTCCAGACG 2919
Madinah clone 1         2674 ---TCTCTCTCAACTTGGATACCATGAGGTGGGCCTTGGAAAGTTT--TGGAATAAGCA-----GGAGGAATTCTCAAATCTTCCAGACG 2754
Madinah clone 2         2202 AGTTCCACCTGAAGAAGCAGGGGCTGCAGTAGCTGCCGAATCTTCTACTGGTACATGGACAACTGTGTGGACTGATGGACTTACCAGCCT 2291
Lemna japonica          3507 AGTTCCACCTGAAGAAGCAGGGGCTGCAGTAGCTGCCGAATCTTCTACTGGTACATGGACAACTGTGTGGACTGATGGACTTACCAGCCT 3596
Lemna perpusilla        2709 AGTTCCACCTGAAGAAGCAGGTGCTGCAGTAGCTGCCGAATCTTCTACTGGTACATGGACAACTGTGTGGACTGATGGACTTACCAGCCT 2798
Landoltia punctata      3412 AGTTCCTGCTGAAGAAGCAGGGGCTGCAGTAGCTGCCGAATCTTCTACCGGTACATGGACAACTGTGTGGACTGATGGACTTACCAGCCT 3501
Spirodela polyrhiza     3776 AGTTCCACCTGAAGAAGCAGGGGCTGCAGTAGCTGCCGAATCTTCTACTGGTACATGGACAACTGTGTGGACTGATGGACTTACCAGCCT 3865
Lemna gibba             3535 AGTTCCACCTGAAGAAGCAGGGGCTGCAGTAGCTGCCGAATCTTCTACTGGTACATGGACAACTGTGTGGACTGATGGACTTACCAGCCT 3624
Lemna aequinoctialis    2818 AGTTCCACCTGAAGAAGCAGGTGCTGCAGTAGCTGCCGAATCTTCTACTGGTACATGGACAACTGTGTGGACTGATGGACTTACCAGCCT 2907
Lemna minor             3487 AGTTCCACCTGAAGAAGCAGGTGCTGCAGTAGCTGCCGAATCTTCTACTGGTACATGGACAACTGTGTGGACTGATGGACTTACCAGCCT 3576

                                     4150      4160      4170      4180      4190      4200      4210      4220      4230
                             ....|....|....|....|....|....|....|....|....|....|....|....|....|....|....|....|....|....|
Riyadh clone            3073 TGATCGTTACAAAGGACGATGCTACCATATCGAACCTGTTGCTGGAGAAGAAAATCAATTTATTGCTTATGTAGCTTACCCATTAGACCT 3162
Dhahran clone           2931 TAGAGCTCGTAAAG-------CTTTAAACCCAAATACGTTACCTACAATGGAGGTAAACATGTTAGTAACAGAACCTTCTTCAAAAAGGT 3013
Tohama clone            2897 TAGAGCTCGTAAAG-------CTTTAAACCCAAATACGTTACCTACAATGGAGGTAAACATGTTAGTAACAGAACCTTCTTCAAAAAGGT 2979
Al-Baha clone           2225 TGATCGTTACAAAGGACGATGCTACCATATCGAACCTGTTGCTGGAGAAGAAAATCAATTTATTGCTTATGTAGCTTACCCATTAGACCT 2314
Jazan clone             2791 TGATCGTTACAAAGGACGATGCTACCATATCGAACCCGTTGCTGGAGAAGAAAATCAATATATTGCTTATGTAGCTTACCCATTAGACCT 2880
Al-Taif clone           2832 TGATCGTTACAAAGGACGATGCTACCATATCGAACCTGTTGCTGGAGAAGAAAATCAATTTATTGCTTATGTAGCTTACCCATTAGACCT 2921
Tanomah clone           2803 TGATCGTTACAAAGGACGATGCTACCATATCGAACCTGTTGCTGGAGAAGAAAATCAATTTATTGCTTATGTAGCTTACCCATTAGACCT 2892
Al-Qassim clone         2920 TAGAGCTCGTAAAG-------CTTTAAACCCAAATACATTACCTACAATGGAAGTAAACATGTTAGTAACAGAACCTTCTTCAAAAAGGT 3002
Madinah clone 1         2755 TAGAGCTCGTAAAG-------CTTTAAACCCAAATACGTTACCTACAATGGAGGTAAACATGTTAGTAACAGAACCTTCTTCAAAAAGGT 2837
Madinah clone 2         2292 TGATCGTTACAAAGGACGATGCTACCATATCGAACCCGTTGTTGGAGAGGAAAATCAATATATTGCTTATGTAGCTTACCCTTTAGACCT 2381
Lemna japonica          3597 TGATCGTTACAAAGGACGATGCTACCATATCGAACCCGTTGCTGGAGAAGAAAATCAATTTATTGCTTATATAGCTTACCCATTAGACCT 3686
Lemna perpusilla        2799 TGATCGTTACAAAGGACGATGCTACCATATCGAACCCGTTGTTGGAGAGGAAAATCAATTTATTGCTTATGTAGCTTACCCATTAGACCT 2888
Landoltia punctata      3502 TGATCGTTACAAAGGACGATGCTACCATATCGAACCCGTTGCTGGAGAAGAAAATCAATATATTGCTTATGTAGCGTACCCATTAGACCT 3591
Spirodela polyrhiza     3866 TGATCGTTACAAAGGACGATGCTACCATATCGAACCCGTTGTTGGAGAGGAAAATCAATATATTGCTTATGTAGCTTACCCTTTAGACCT 3955
Lemna gibba             3625 TGATCGTTACAAAGGACGATGCTACCATATCGAACCTGTTGCTGGAGAAGAAAATCAATTTATTGCTTATGTAGCTTACCCATTAGACCT 3714
Lemna aequinoctialis    2908 TGATCGTTACAAAGGACGATGCTACCATATCGAACCCGTTGCTGGAGAAGAAAATCAATTTATTGCTTATGTAGCTTACCCATTAGACCT 2997
Lemna minor             3577 TGATCGTTACAAAGGACGATGCTACCATATCGAACCCGTTGCTGGAGAAGAAAATCAATATATTGCTTATGTAGCTTACCCATTAGACCT 3666

                                     4240      4250      4260      4270      4280      4290      4300      4310      4320
                             ....|....|....|....|....|....|....|....|....|....|....|....|....|....|....|....|....|....|
Riyadh clone            3163 TTTTGAAGAAGGTTCTGTTACTAACATGTTTACCTCCATTGTAGGTAACGTATTTGGGTTTAAAGCTTTACGAGCTCTACGTTTGGAAGA 3252
Dhahran clone           3014 CTAATGGGTAAGCTACATAAGCAATAAATTGATTTTCTTCTCCAGCAACAGGTTCGATATGGTAGCAT--CGTCCTTTGTAACGATCAAG 3101
```

```
Tohama clone            2980 CTAATGGGTAAGCTACATAAGCAATAAATTGATTTTCTTCTCCAGCAACAGGTTCGATATGGTAGCAT--CGTCCTTTGTAACGATCAAG 3067
Al-Baha clone           2315 TTTTGAAGAAGGTTCTGTTACTAACATGTTTACCTCCATTGTAGGTAACGTATTTGGGTTTAAAGCTTTACGAGCTCTACGTTTGGAAGA 2404
Jazan clone             2881 TTTTGAAGAAGGTTCTGTTACTAACATGTTTACCTCCATTGTAGGTAACGTATTTGGGTTTAAAGCTTTACGAGCTCTACGTCTGGAAGA 2970
Al-Taif clone           2922 TTTTGAAGAAGGTTCTGTTACTAACATGTTTACCTCCATTGTAGGTAACGTATTTGGGTTTAAAGCTTTACGAGCTCTACGTTTGGAAGA 3011
Tanomah clone           2893 TTTTGAAGAAGGTTCTGTTACTAACATGTTTACCTCCATTGTAGGTAACGTATTTGGGTTTAAAGCTTTACGAGCTCTACGTTTGGAAGA 2982
Al-Qassim clone         3003 CTAATGGGTACGCTACATAAGCAATATATTGATTTTCTTCTCCAGCAACGGGTTCGATATGGTAGCAT--CGTCCTTTGTAACGATCAAG 3090
Madinah clone 1         2838 CTAATGGGTAAGCTACATAAGCAATATATTGATTTTCTTCTCCAGCAACGGGTTCGATATGGTAGCAT--CGTCCTTTGTAACGATCAAG 2925
Madinah clone 2         2382 TTTTGAAGAAGGTTCTGTTACTAACATGTTTACTTCCATTGTAGGTAATGTATTTGGGTTTAAAGCTTTACGAGCTCTACGTCTGGAAGA 2471
Lemna japonica          3687 TTTTGAAGAAGGTTCTGTTACTAACATGTTTACCTCCATTGTAGGTAACGTATTTGGGTTTAAAGCTTTACGAGCTCTACGTTTGGAAGA 3776
Lemna perpusilla        2889 TTTTGAAGAAGGTTCTGTTACTAACATGTTTACCTCCATTGTAGGTAACGTATTTGGGTTTAAAGCTTTACGAGCTCTACGTCTGGAAGA 2978
Landoltia punctata      3592 TTTTGAAGAAGGTTCTGTTACTAACATGTTTACTTCCATTGTAGGTAATGTATTTGGGTTTAAAGCTTTACGAGCTCTACGTCTGGAAGA 3681
Spirodela polyrhiza     3956 TTTTGAAGAAGGTTCTGTTACTAACATGTTTACTTCCATTGTAGGTAATGTATTTGGGTTTAAAGCTTTACGAGCTCTACGTCTGGAAGA 4045
Lemna gibba             3715 TTTTGAAGAAGGTTCTGTTACTAACATGTTTACCTCCATTGTAGGTAACGTATTTGGGTTTAAAGCTTTACGAGCTCTACGTTTGGAAGA 3804
Lemna aequinoctialis    2998 TTTTGAAGAAGGTTCTGTTACTAACATGTTTACCTCCATTGTAGGTAACGTATTTGGGTTTAAAGCTTTACGAGCTCTACGTCTGGAAGA 3087
Lemna minor             3667 TTTTGAAGAAGGTTCTGTTACTAACATGTTTACCTCCATTGTAGGTAACGTATTTGGGTTTAAAGCTTTACGAGCTCTACGTCTGGAAGA 3756

                                      4330      4340      4350      4360      4370      4380      4390      4400      4410
                             ....|....|....|....|....|....|....|....|....|....|....|....|....|....|....|....|....|....|
Riyadh clone            3253 TTTGCGAATTCCTCCTGCGTATTCCAAAACTTTCCAAGGCCCACCTCATGGGATCCAAGTTGAGAGAGATAAATTGAACAAGTATGGTCG 3342
Dhahran clone           3102 GCTGGTAAGTCCATCAGTCCA---CACAGTTGTCCATGTACCA---------------GTAGA-------AGATTCGGCAGCTACTGCAG 3166
Tohama clone            3068 GCTGGTAAGTCCATCAGTCCA---CACAGTTGTCCATGTACCA---------------GTAGA-------AGATTCGGCAGCTACTGCAG 3132
Al-Baha clone           2405 TTTGCGAATTCCTCCTGCGTATTCCAAAACTTTCCAAGGCCCACCTCATGGGATCCAAGTTGAGAGAGATAAATTGAACAAGTATGGTCG 2494
Jazan clone             2971 TTTGAGAATTCCTCCTGCTTATTCCAAAACTTTCCAAGGCCCACCTCATGGTATCCAAGTTGAGAGAGATAAATTGAACAAGTATGGTCG 3060
Al-Taif clone           3012 TTTGCGAATTCCTCCTGCGTATTCCAAAACTTTCCAAGGCCCACCTCATGGGATCCAAGTTGAGAGAGATAAATTGAACAAGTATGGTCG 3101
Tanomah clone           2983 TTTGCGAATTCCTCCTGCGTATTCCAAAACTTTCCAAGGCCCACCTCATGGGATCCAAGTTGAGAGAGATAAATTGAACAAGTATGGTCG 3072
Al-Qassim clone         3091 GCTGGTAAGTCCATCAGTCCA---CACAGTTGTCCATGTACCG---------------GTAGA-------AGATTCGGCAGCTACTGCAG 3155
Madinah clone 1         2926 GCTGGTAAGTCCATCAGTCCA---CACAGTTGTCCATGTACCA---------------GTAGA-------AGATTCGGCAGCTACTGCAG 2990
Madinah clone 2         2472 TTTGCGAATTCCTCCTGCTTATTCCAAAACTTTCCAAGGCCCACCTCATGGGATCCAAGTTGAGAGAGATAAATTGAACAAGTATGGTCG 2561
Lemna japonica          3777 TTTGCGAATTCCTCCTGCTTATTCCAAAACTTTCCAAGGCCCACCTCATGGGATCCAAGTTGAGAGAGATAAATTGAACAAGTATGGTCG 3866
Lemna perpusilla        2979 TTTGAGAATTCCTCCTGCTTATTCCAAAACTTTCCAAGGCCCACCTCATGGTATCCAAGTTGAGAGAGATAAATTGAACAAGTATGGTCG 3068
Landoltia punctata      3682 TTTGCGAATTCCTCCTGCTTATTCCAAAACTTTCCTAGGCCCACCTCATGGGATCCAAGTTGAGAGAGATAAATTGAACAAGTATGGTCG 3771
Spirodela polyrhiza     4046 TTTGCGAATTCCTCCTGCTTATTCCAAAACTTTCCAAGGCCCACCTCATGGGATCCAAGTTGAGAGAGATAAATTGAACAAGTATGGTCG 4135
Lemna gibba             3805 TTTGCGAATTCCTCCTGCGTATTCCAAAACTTTCCAAGGCCCACCTCATGGGATCCAAGTTGAGAGAGATAAATTGAACAAGTATGGTCG 3894
Lemna aequinoctialis    3088 TTTGAGAATTCCTCCTGCTTATTCCAAAACTTTCCAAGGCCCACCTCATGGTATCCAAGTTGAGAGAGATAAATTGAACAAGTATGGTCG 3177
Lemna minor             3757 TTTGAGAATTCCTCCTGCTTATTCCAAAACTTTCCAAGGCCCACCTCATGGTATCCAAGTTGAGAGAGATAAATTGAACAAGTATGGTCG 3846

                                      4420      4430      4440      4450      4460      4470      4480      4490      4500
                             ....|....|....|....|....|....|....|....|....|....|....|....|....|....|....|....|....|....|
Riyadh clone            3343 TCCTCTATTGGGATGTACCATCAAACCAAAATTGGCATTATCCGCGAAAAACTACGGTAGAGCGGTTTATGAATGTCTACGTGGTGGACT 3432
Dhahran clone           3167 CCCC---------TGCTTCTTCAGGTGGAACTCCAGGTT------GAGGAGTTACT-CGGAATGCTGCCAAGATATCCGTATCTTTTGTC 3240
Tohama clone            3133 CCCC---------TGCTTCTTCAGGTGGAACTCCAGGTT------GAGGAGTTACT-CGGAATGCTGCCAAGATATCCGTATCTTTTGTC 3206
Al-Baha clone           2495 TCCTCTATTGGGATGTACCATCAAACCAAAATTGGCATTATCCGCGAAAAACTACGGTAGAGCGGTTTATGAATGTCTACGTGGTGGACT 2584
Jazan clone             3061 TCCTCTATTGGGATGTACCATCAAACCAAAATTGGCATTATCCGCGAAAAACTACGGTAGAGCGGTTTATGAATGTCTACGTGGTGGACT 3150
Al-Taif clone           3102 TCCTCTATTGGGATGTACCATCAAACCAAAATTGGCATTATCCGCGAAAAACTACGGTAGAGCGGTTTATGAATGTCTACGTGGTGGACT 3191
Tanomah clone           3073 TCCTCTATTGGGATGTACCATCAAACCAAAATTGGCATTATCCGCGAAAAACTACGGTAGAGCGGTTTATGAATGTCTACGTGGTGGACT 3162
Al-Qassim clone         3156 CCCC---------TGCTTCTTCAGCAGGAACTCCAGGTT------GAGGAGTTACT-CGGAATGCTGCCAAGATATCCGTATCTTTTGTC 3229
Madinah clone 1         2991 CACC---------TGCTTCTTCAGGTGGAACTCCAGGTT------GAGGAGTTACT-CGGAATGCTGCCAAGATATCCGTATCTTTTGTC 3064
Madinah clone 2         2562 TCCTCTATTGGGATGTACCATCAAACCAAAATTGGCATTATCCGCGAAAAACTACGGTAGAGCGGTTTATGAATGTCTACGTGGTGGACT 2651
Lemna japonica          3867 TCCTCTATTGGGATGTACCATCAAACCAAAATTGGCATTATCCGCGAAAAACTACGGTAGAGCGGTTTATGAATGTCTACGTGGTGGACT 3956
Lemna perpusilla        3069 TCCTCTATTGGGATGTACCATCAAACCAAAATTGGCATTATCTGCGAAAAACTACGGTAGAGCGGTTTATGAATGTCTACGTGGTGGACT 3158
Landoltia punctata      3772 TCCTCTATTGGGATGTACCATCAAACCAAAATTGGCATTATCCGCGAAAAACTACGGTAGAGCGGTTTATGAATGTCTACGTGGTGGACT 3861
Spirodela polyrhiza     4136 TCCTCTATTGGGATGTACCATCAAACCAAAATTGGCATTATCCGCGAAAAACTACGGTAGAGCGGTTTATGAATGTCTACGTGGTGGACT 4225
Lemna gibba             3895 TCCTCTATTGGGATGTACCATCAAACCAAAATTGGCATTATCCGCGAAAAACTACGGTAGAGCGGTTTATGAATGTCTACGTGGTGGACT 3984
Lemna aequinoctialis    3178 TCCTCTATTGGGATGTACCATCAAACCAAAATTGGCATTATCCGCGAAAAACTACGGTAGAGCGGTTTATGAATGTCTACGTGGTGGACT 3267
Lemna minor             3847 TCCTCTATTGGGATGTACCATCAAACCAAAATTGGCATTATCCGCGAAAAACTACGGTAGAGCGGTTTATGAATGTCTACGTGGTGGACT 3936

                                      4510      4520      4530      4540      4550      4560      4570      4580      4590
                             ....|....|....|....|....|....|....|....|....|....|....|....|....|....|....|....|....|....|
Riyadh clone            3433 TGATTTTACCAAGGATGATGAAAACGTAAACTCACAACCATTTATGC-GTTGG------------------------------------- 3484
Dhahran clone           3241 TCAT---ACTCAGGAGTATAATAA-GTCAATTTGTAATCTTTAACACCAGCTTTGAATCCAGCACTTGCTTTAGTTTCTGT--------- 3317
Tohama clone            3207 TCAT---ACTCAGGAGTATAATAA-GTCAATTTGTAATCTTTAACACCAGCTTTGAATCCAGCACTTGCTTTAGTTTCTGTT-------- 3284
Al-Baha clone           2585 TGATTTTACCAAGGATGATGAAAACGTAAACTCACAACCATTTATGC-GTTGGAGAGATCGT---------------------------- 2645
Jazan clone             3151 TGATTTTACCAAGGATGATGAAAACGTAAACTCACAACCATTTATGC-GTTGGAGAGACCGTTTCCTATTTTGTGCGGAAGCAATTTATA 3239
Al-Taif clone           3192 TGATTTTACCAAGGATGATGAAAACGTAAACTCACAACCATTTATGC-GTTGGAGAGATCGT---------------------------- 3252
Tanomah clone           3163 TGATTTTACCAAGGATGATGAAAACGTAAACTCACAACCATTTATGC-GTTGGAGAGATCGT---------------------------- 3223
Al-Qassim clone         3230 TCAT---ACTCAGGAGTATAATAA-GTCAATTTGTAATCTTTAACACCAGCTTTGAATCCAGCACTTGCTTTAGTCTCTGT--------- 3306
Madinah clone 1         3065 TCAT---ACTCAGGAGTATAATAA-GTCAATTTGTAATCTTTAACACCAGCTTTGAATCCAGCACTTGCTTTAGTCTCTG---------- 3140
Madinah clone 2         2652 TGATTTTACCAAGGATGATGAAAACGTGAACTCACAACCATTTATGC-GTTGGAG----------------------------------- 2705
Lemna japonica          3957 TGATTTTACCAAGGATGATGAAAACGTAAACTCACAACCATTTATGC-GTTGGAGAGACCGTTTCTTATTTTGTGCCGAAGCAATTTATA 4045
Lemna perpusilla        3159 TGATTTTACCAAGGATGATGAAAACGTGAACTCACAACCATTTATGC-GTTGGAGAGACCGTTTCCTATTTTGTGCGGAAGCAATTTATA 3247
Landoltia punctata      3862 TGATTTTACCAAGGATGATGAAAACGTAAACTCACAACCATTTATGC-GTTGGAGAGACCGTTTCTTATTTTGTGCTGAAGCAATTTATA 3950
Spirodela polyrhiza     4226 TGATTTTACCAAGGATGATGAAAACGTGAACTCACAACCATTTATGC-GTTGGAGAGACCGTTTCTTATTTTGTGCTGAAGCAATTTATA 4314
Lemna gibba             3985 TGATTTTACCAAGGATGATGAAAACGTAAACTCACAACCATTTATGC-GTTGGAGAGATCGT---------------------------- 4045
Lemna aequinoctialis    3268 TGATTTTACCAAGGATGATGAAAACGTAAACTCACAACCATTTATGC-GTTGGAGAGACCGTTTCCTATTTTGTGCGGAAGCAATTTTTA 3356
Lemna minor             3937 TGATTTTACCAAGGATGATGAAAACGTAAACTCACAACCATTTATGC-GTTGGAGAGACCGTTTCCTATTTTGTGCGGAAGCAATTTATA 4025

                                      4600      4610      4620      4630      4640      4650      4660      4670      4680
                             ....|....|....|....|....|....|....|....|....|....|....|....|....|....|....|....|....|....|
Riyadh clone            3484 ------------------------------------------------------------------------------------------ 3484
Dhahran clone           3317 ------------------------------------------------------------------------------------------ 3317
Tohama clone            3284 ------------------------------------------------------------------------------------------ 3284
Al-Baha clone           2645 ------------------------------------------------------------------------------------------ 2645
Jazan clone             3240 AATCACAAGCCGAAACAGGTGAAATTAAAGGGCATTACCTAAAT---------------------------------------------- 3283
Al-Taif clone           3252 ------------------------------------------------------------------------------------------ 3252
Tanomah clone           3223 ------------------------------------------------------------------------------------------ 3223
Al-Qassim clone         3306 ------------------------------------------------------------------------------------------ 3306
Madinah clone 1         3140 ------------------------------------------------------------------------------------------ 3140
Madinah clone 2         2705 ------------------------------------------------------------------------------------------ 2705
```

```
Lemna japonica         4046 AAGCACAAGCCGAAACAGGTGAAATTAAAGGGCATTACCTAAATGCTACTGCAGGTACTTGTGAAGAAATGATCAAAAGGGCTGTGTTTG 4135
Lemna perpusilla       3248 AATCACAAGCCGAAACAGGTGAAATTAAAGGGCATTACCTAAATGCTACTGCAGGTACTTGTGAAGAAATGATCAAAAGGGCTGTGTTTG 3337
Landoltia punctata     3951 AAGCACAAGCTGAAACAGGTGAAATTAAAGGGCATTACCTAAATGCTACTGCAGGTAATTGTGAAGACATGATGAAAAGGGCTGTGTTTG 4040
Spirodela polyrhiza    4315 AAGCACAAGCTGAAACAGGTGAAATTAAAGGGCATTACTTAAATGCTACTGCAGGTACATGCGA-------------------------- 4378
Lemna gibba            4045 ------------------------------------------------------------------------------------------ 4045
Lemna aequinoctialis   3357 AATCACAAGCCGAAACAGGTGAAATTAAAGGGCATTACCTAAATGCTACTGCAGGTACTTGTGAAGAAATGATCAAAAGGGCTGTGTTTG 3446
Lemna minor            4026 AATCACAAGCCGAAACAGGTGAAATTAAAGGGCATTACCTAAAT---------------------------------------------- 4069

                                  4690      4700      4710      4720      4730      4740      4750      4760      4770
                            ....|....|....|....|....|....|....|....|....|....|....|....|....|....|....|....|....|....|
Riyadh clone           3484 ------------------------------------------------------------------------------------------ 3484
Dhahran clone          3317 ------------------------------------------------------------------------------------------ 3317
Tohama clone           3284 ------------------------------------------------------------------------------------------ 3284
Al-Baha clone          2645 ------------------------------------------------------------------------------------------ 2645
Jazan clone            3283 ------------------------------------------------------------------------------------------ 3283
Al-Taif clone          3252 ------------------------------------------------------------------------------------------ 3252
Tanomah clone          3223 ------------------------------------------------------------------------------------------ 3223
Al-Qassim clone        3306 ------------------------------------------------------------------------------------------ 3306
Madinah clone 1        3140 ------------------------------------------------------------------------------------------ 3140
Madinah clone 2        2705 ------------------------------------------------------------------------------------------ 2705
Lemna japonica         4136 CTAGAGAATTGGGAGTCCCTATTGTAATGCATGACTACTTAACAGGGGGATTCACTGCAAATACTTCTTTAGCATATTATTGCCGAGACA 4225
Lemna perpusilla       3338 CGAGAGAATTGGGAGTTCCTATTGTAATGCATGACTACTTAACAGGGGGATTCACTGCAAATACTAGTTTAGCACATTATTGCCGAGACA 3427
Landoltia punctata     4041 CTAGAGAATTGGCAGTACCTATTGTAATGCATGACTACTTAACAGGTGGATTCACTGCAAATACTAGTTTAGCAGATTATTGCCGAGACA 4130
Spirodela polyrhiza    4378 ------------------------------------------------------------------------------------------ 4378
Lemna gibba            4045 ------------------------------------------------------------------------------------------ 4045
Lemna aequinoctialis   3447 CGAGAGAATTGGGAGTTCCTATTGTAATGCATGACTACTTAACAGGTGGATTCACTGCAAATACTAGTTTAGCACATTATTGCCGAGACA 3536
Lemna minor            4069 ------------------------------------------------------------------------------------------ 4069

                                  4780      4790      4800      4810      4820      4830      4840      4850      4860
                            ....|....|....|....|....|....|....|....|....|....|....|....|....|....|....|....|....|....|
Riyadh clone           3484 ------------------------------------------------------------------------------------------ 3484
Dhahran clone          3317 ------------------------------------------------------------------------------------------ 3317
Tohama clone           3284 ------------------------------------------------------------------------------------------ 3284
Al-Baha clone          2645 ------------------------------------------------------------------------------------------ 2645
Jazan clone            3283 ------------------------------------------------------------------------------------------ 3283
Al-Taif clone          3252 ------------------------------------------------------------------------------------------ 3252
Tanomah clone          3223 ------------------------------------------------------------------------------------------ 3223
Al-Qassim clone        3306 ------------------------------------------------------------------------------------------ 3306
Madinah clone 1        3140 ------------------------------------------------------------------------------------------ 3140
Madinah clone 2        2705 ------------------------------------------------------------------------------------------ 2705
Lemna japonica         4226 ACGGCCTACTTCTTCACATCCACCGCGCAATGCATGCAGTTATTGATAGACAGAAAAATCATGGTATGCATTTCCGTGTACTAGCTAAAG 4315
Lemna perpusilla       3428 ATGGCCTACTTCTTCACATCCACCGCGCAATGCATGCAGTTATTGATAGACAGAAAAATCATGGTATGCATTTCCGTGTACTAGCTAAAG 3517
Landoltia punctata     4131 ACGGCCTACTTCTTCACATCCACCGCGCAATGCATGCAGTTATTGATAGACAGAAAAATCATGGTATGCATTTCCGTGTACTAGCTAAAG 4220
Spirodela polyrhiza    4378 ------------------------------------------------------------------------------------------ 4378
Lemna gibba            4045 ------------------------------------------------------------------------------------------ 4045
Lemna aequinoctialis   3537 ATGGTCTACTTCTTCACATCCACCGCGCAATGCATGCAGTTATTGATAGACAGAAAAATCATGGTATGCATTTCCGTGTACTAGCTAAAG 3626
Lemna minor            4069 ------------------------------------------------------------------------------------------ 4069

                                  4870      4880      4890      4900      4910      4920      4930      4940      4950
                            ....|....|....|....|....|....|....|....|....|....|....|....|....|....|....|....|....|....|
Riyadh clone           3484 ------------------------------------------------------------------------------------------ 3484
Dhahran clone          3317 ------------------------------------------------------------------------------------------ 3317
Tohama clone           3284 ------------------------------------------------------------------------------------------ 3284
Al-Baha clone          2645 ------------------------------------------------------------------------------------------ 2645
Jazan clone            3283 ------------------------------------------------------------------------------------------ 3283
Al-Taif clone          3252 ------------------------------------------------------------------------------------------ 3252
Tanomah clone          3223 ------------------------------------------------------------------------------------------ 3223
Al-Qassim clone        3306 ------------------------------------------------------------------------------------------ 3306
Madinah clone 1        3140 ------------------------------------------------------------------------------------------ 3140
Madinah clone 2        2705 ------------------------------------------------------------------------------------------ 2705
Lemna japonica         4316 CATTACGTATGTCTGGGGGGGATCACGTTCACTCTGGTACAGTAGTAGGTAAACTGGAAGGTGAACGTGAGATGACTTTAGGTTTTGTTG 4405
Lemna perpusilla       3518 CACTACGTATGTCTGGTGGGGATCATATTCACTCTGGTACAGTAGTAGGTAAACTAGAAGGTGAACGTGAGATGACTTTAGGTTTCGTTG 3607
Landoltia punctata     4221 CATTACGTATGTCTGGTGGGGATCATATTCACGCTGGTACAGTAGTAGGTAAACTGGAAGGTGAACGTGAGATGACTTTAGGTTTTGTTG 4310
Spirodela polyrhiza    4378 ------------------------------------------------------------------------------------------ 4378
Lemna gibba            4045 ------------------------------------------------------------------------------------------ 4045
Lemna aequinoctialis   3627 CACTACGTATGTCTGGTGGGGATCACATTCACTCTGGTACAGTAGTAGGTAAACTAGAAGGTGAACGTGAGATGACTTTAGGTTTCGTTG 3716
Lemna minor            4069 ------------------------------------------------------------------------------------------ 4069

                                  4960      4970      4980      4990      5000      5010      5020      5030      5040
                            ....|....|....|....|....|....|....|....|....|....|....|....|....|....|....|....|....|....|
Riyadh clone           3484 ------------------------------------------------------------------------------------------ 3484
Dhahran clone          3317 ------------------------------------------------------------------------------------------ 3317
Tohama clone           3284 ------------------------------------------------------------------------------------------ 3284
Al-Baha clone          2645 ------------------------------------------------------------------------------------------ 2645
Jazan clone            3283 ------------------------------------------------------------------------------------------ 3283
Al-Taif clone          3252 ------------------------------------------------------------------------------------------ 3252
Tanomah clone          3223 ------------------------------------------------------------------------------------------ 3223
Al-Qassim clone        3306 ------------------------------------------------------------------------------------------ 3306
Madinah clone 1        3140 ------------------------------------------------------------------------------------------ 3140
Madinah clone 2        2705 ------------------------------------------------------------------------------------------ 2705
Lemna japonica         4406 ATTTATTACGTGATGATTTTATTGAAAAAGACAGAAGTCGTGGTATTTTCTTCACTCAAGATTGGGTCTCTATGCCAGGTGTTCTGCCTG 4495
Lemna perpusilla       3608 ATTTATTACGTGATGATTTTATTGAAAAAGACAGAAGTCGTGGTATTTTCTTCACTCAAGATTGGGTCTCTATGCCAGGTGTTATCCCTG 3697
Landoltia punctata     4311 ATTTATTACGTGATGATTTCATTGAAAAAGACAGAAGTCGTGGTATTTTCTTCACTCAAGATTGGGTCTCTATGCCAGGTGTTCTGCCTG 4400
Spirodela polyrhiza    4378 ------------------------------------------------------------------------------------------ 4378
Lemna gibba            4045 ------------------------------------------------------------------------------------------ 4045
Lemna aequinoctialis   3717 ATTTATTACGTGATGATTTTATTGAAAAAGACAGAAGTCGTGGTATTTTCTTCACTCAAGATTGGGTCTCTATGCCAGGTGTTATCCCTG 3806
Lemna minor            4069 ------------------------------------------------------------------------------------------ 4069
```

```
                                     5050       5060       5070       5080       5090       5100       5110       5120       5130
                               ....|....|....|....|....|....|....|....|....|....|....|....|....|....|....|....|....|....|
Riyadh clone              3484 ------------------------------------------------------------------------------------------ 3484
Dhahran clone             3317 ------------------------------------------------------------------------------------------ 3317
Tohama clone              3284 ------------------------------------------------------------------------------------------ 3284
Al-Baha clone             2645 ------------------------------------------------------------------------------------------ 2645
Jazan clone               3283 ------------------------------------------------------------------------------------------ 3283
Al-Taif clone             3252 ------------------------------------------------------------------------------------------ 3252
Tanomah clone             3223 ------------------------------------------------------------------------------------------ 3223
Al-Qassim clone           3306 ------------------------------------------------------------------------------------------ 3306
Madinah clone 1           3140 ------------------------------------------------------------------------------------------ 3140
Madinah clone 2           2705 ------------------------------------------------------------------------------------------ 2705
Lemna japonica            4496 TAGCTTCAGGTGGTATTCATGTTTGGCATATGCCTGCTCT-------------------------------------------------- 4535
Lemna perpusilla          3698 TAGCTTCAGGTGGTATTCATGTTTGGCATATGCCTGCTCTAACCGAGATCTTTGGAGATGATTCCGTACTACAGTTTGGTGGCGGAACTT 3787
Landoltia punctata        4401 TAGCTTCCGGGGGTATTCATGTTTGGCATATGCCTGCCCTGACCGAGATCTTTGGAGATGATTCCGTACTACAGTTTGGTGGCGGAACTT 4490
Spirodela polyrhiza       4378 ------------------------------------------------------------------------------------------ 4378
Lemna gibba               4045 ------------------------------------------------------------------------------------------ 4045
Lemna aequinoctialis      3807 TAGCTTCAGGTGGTATTCATGTTTGGCATATGCCTGCTCTAACCGAGATCTTTGGAGATGATTCCGTACTACAGTTTGGTGGCGGAACTT 3896
Lemna minor               4069 ------------------------------------------------------------------------------------------ 4069

                                     5140       5150       5160       5170       5180       5190       5200       5210       5220
                               ....|....|....|....|....|....|....|....|....|....|....|....|....|....|....|....|....|....|
Riyadh clone              3484 ------------------------------------------------------------------------------------------ 3484
Dhahran clone             3317 ------------------------------------------------------------------------------------------ 3317
Tohama clone              3284 ------------------------------------------------------------------------------------------ 3284
Al-Baha clone             2645 ------------------------------------------------------------------------------------------ 2645
Jazan clone               3283 ------------------------------------------------------------------------------------------ 3283
Al-Taif clone             3252 ------------------------------------------------------------------------------------------ 3252
Tanomah clone             3223 ------------------------------------------------------------------------------------------ 3223
Al-Qassim clone           3306 ------------------------------------------------------------------------------------------ 3306
Madinah clone 1           3140 ------------------------------------------------------------------------------------------ 3140
Madinah clone 2           2705 ------------------------------------------------------------------------------------------ 2705
Lemna japonica            4535 ------------------------------------------------------------------------------------------ 4535
Lemna perpusilla          3788 TAGGACACCCTTGGGGTAATGCACCTGGTGCAGTAGCTAACCGTGTAGCTTTAGAAGCGTGTGTACAAGCTCGTAATGAGGGACGTGACC 3877
Landoltia punctata        4491 TAGGACACCCTTGGGGAAATGCACCTGGTGCAGTAGCTAACCGTGTAGCTTTAGAAGCGTGTGTACAAGCTCGTAATGAGGGACGTGACC 4580
Spirodela polyrhiza       4378 ------------------------------------------------------------------------------------------ 4378
Lemna gibba               4045 ------------------------------------------------------------------------------------------ 4045
Lemna aequinoctialis      3897 TAGGACACCCTTGGGGTAATGCACCTGGTGCAGTAGCTAACCGTGTAGCTTTAGAAGCGTGTGTACAAGCTCGTAATGAGGGACGTGACC 3986
Lemna minor               4069 ------------------------------------------------------------------------------------------ 4069

                                     5230       5240       5250       5260       5270       5280
                               ....|....|....|....|....|....|....|....|....|....|....|....|....|
Riyadh clone              3484 ----------------------------------------------------------------- 3484
Dhahran clone             3317 ----------------------------------------------------------------- 3317
Tohama clone              3284 ----------------------------------------------------------------- 3284
Al-Baha clone             2645 ----------------------------------------------------------------- 2645
Jazan clone               3283 ----------------------------------------------------------------- 3283
Al-Taif clone             3252 ----------------------------------------------------------------- 3252
Tanomah clone             3223 ----------------------------------------------------------------- 3223
Al-Qassim clone           3306 ----------------------------------------------------------------- 3306
Madinah clone 1           3140 ----------------------------------------------------------------- 3140
Madinah clone 2           2705 ----------------------------------------------------------------- 2705
Lemna japonica            4535 ----------------------------------------------------------------- 4535
Lemna perpusilla          3878 TTGCTCGTGAAGGTAATGAAATTATCCGTGAAGCTTGCAAATGGAGTCCTGAATTAGCTGCTGCT 3942
Landoltia punctata        4581 TTGCTCGTGAAGGTAATGAAATTATCCGTGAAGCTTGCAAATGGAGTCCTGAATTAGCCGCTGCT 4645
Spirodela polyrhiza       4378 ----------------------------------------------------------------- 4378
Lemna gibba               4045 ----------------------------------------------------------------- 4045
Lemna aequinoctialis      3987 TTGCTCGTGAAGGTAATGAAATTATCCGTGAAGCTTGCAAATGGAGTCCTGAATTAGCTGCTGCT 4051
Lemna minor               4069 ----------------------------------------------------------------- 4069
```

**Figure S1.** Multiple nucleotide sequence alignments of the concatenated sequences of psbK-psbI, trnH-psbA, and atpF-atpH intergenic spacer region, and matK, rpoC1, and rbcL genes generated using ClustalW alignment software. The identical nucleotides are shaded in black.

| **Name** | | | | | | | | | | | | | | | | | |
|---|---|---|---|---|---|---|---|---|---|---|---|---|---|---|---|---|---|
| **Riyadh clone** | *** | | | | | | | | | | | | | | | | |
| **Dhahran clone** | 0.262 | *** | | | | | | | | | | | | | | | |
| **Tohama clone** | 0.219 | 0.245 | *** | | | | | | | | | | | | | | |
| **Al-Baha clone** | 0.626 | 0.644 | 0.636 | *** | | | | | | | | | | | | | |
| **Jazan clone** | 0.797 | 0.793 | 0.799 | 0.757 | *** | | | | | | | | | | | | |
| **Al-Taif clone** | 0.491 | 0.5 | 0.491 | 0.438 | 0.734 | *** | | | | | | | | | | | |
| **Tanomah clone** | 0.626 | 0.644 | 0.636 | 0.2 | 0.757 | 0.438 | *** | | | | | | | | | | |
| **Al-Qassim clone** | 0.534 | 0.549 | 0.538 | 0.601 | 0.773 | 0.499 | 0.601 | *** | | | | | | | | | |
| **Madinah clone 1** | 0.797 | 0.793 | 0.799 | 0.757 | 0.2 | 0.734 | 0.757 | 0.773 | *** | | | | | | | | |
| **Madinah clone 2** | 0.846 | 0.838 | 0.845 | 0.811 | 0.718 | 0.778 | 0.811 | 0.782 | 0.718 | *** | | | | | | | |
| *Lemna japonica* | 0.776 | 0.777 | 0.773 | 0.605 | 0.81 | 0.649 | 0.605 | 0.723 | 0.81 | 0.902 | *** | | | | | | |
| *Lemna perpusilla* | 0.789 | 0.793 | 0.789 | 0.593 | 0.786 | 0.649 | 0.593 | 0.726 | 0.786 | 0.92 | 0.444 | *** | | | | | |
| *Landoltia punctata* | 0.75 | 0.746 | 0.747 | 0.592 | 0.754 | 0.619 | 0.592 | 0.601 | 0.754 | 0.845 | 0.473 | 0.562 | *** | | | | |
| *Spirodela polyrhiza* | 0.78 | 0.761 | 0.782 | 0.72 | 0.654 | 0.706 | 0.72 | 0.706 | 0.654 | 0.493 | 0.796 | 0.818 | 0.736 | *** | | | |
| *Lemna gibba* | 0.68 | 0.673 | 0.684 | 0.515 | 0.711 | 0.525 | 0.515 | 0.659 | 0.711 | 0.821 | 0.389 | 0.562 | 0.435 | 0.699 | *** | | |
| *Lemna aequinoctialis* | 0.81 | 0.806 | 0.808 | 0.656 | 0.83 | 0.681 | 0.656 | 0.714 | 0.83 | 0.893 | 0.6 | 0.655 | 0.432 | 0.817 | 0.542 | *** | |
| *Lemna minor* | 0.712 | 0.707 | 0.71 | 0.567 | 0.716 | 0.577 | 0.567 | 0.666 | 0.716 | 0.831 | 0.452 | 0.551 | 0.31 | 0.716 | 0.381 | 0.447 | *** |

**Figure S2.** Divergence score matrix of the concatenated sequences of psbK-psbI, trnH-psbA, and atpF-atpH intergenic spacer region, and matK, rpoC1, and rbcL gene, based on a ClustalW alignment with the BLOSUM62 matrix.

**Table S1.** All sequences with their accessions number used for species identification.

```
>MA_LJ_Ri_SA_psbK-psbI(OK546023)
GTTTGGCAAGCTGCTGTAAGTTTTCGATAAAGTTATTAAAACTCTACTGAAAAAATTCATGATTTATTTGATA
AAAAAGATTCTAATAAAAATTGATAACGTAATAACAATCTTAGTTTATACATCCTCATAAAAAATATTTGAAT
TCTTGTATATTGGATAAAAAGAGCGATAAGTTTGGATCAGTCCATTTCACCGTTCTGGACGCTCTTCCAGTGA
GGAAGTACTTTATTTTTTTATTAGCTTTTGTTTTACACAATACTTTATTGTTATAATTACAGTTAATTTTAGA
ATAAGCTTTTCCGTAATGAACAAGTCATAATCTTAATTTAAAATGCATTCATGAGTTTGAAAATTCAGTTTTT
GTAGAAAAAAAACTTAACTTAAGACTATAAAAAAGAAAGGATTGTTCTTTATTTTTTCATAGTTTTTTCTTGG
CATGCCAAAATAATATATGTGTTACATAACTCAAATGGATAATCTATTCCCTTTTACCCCAAAAATGATCCTA
TCTTGGAGATTGTGTAATGCTTACTC
>MA_LJ_DH_SA_psbK-psbI(OK571365)
TTCGATAAAGTTATTAAAACTCTACTGAAAAAATTCATGATTTATTTGATAAAAAAGATTCTAATAAAAATTG
ATAACGTAATAACAATCTTAGTTTATACATCCTCATAAAAAATATTTGAATTCTTGTATATTGGATAAAAAGA
GCGATAAGTTTGGATCAGTCCATTTCACCGTTCTGGACGCTCTTCCAGTGAGGAAGTACTTTATTTTTTTATT
AGCTTTTGTTTTACACAATACTTTATTGTTATAATTACAGTTAATTTTAGAATAAGCTTTTCCGTAATGAACA
AGTCATAATCTTAATTTAAAATGCATTCATGAGTTTGAAAATTCAGTTTTTGTAGAAAAAAAACTTAACTTAA
GACTATAAAAAAGAAAGGATTGTTCTTTATTTTTTCATAGTTTTTTCTTGGCATGCCAAAATAATATATGTGT
TACATAACTCAAATGGATAATCTATTCCCTTTTACCCCAAAAATGATCCTATCTTGGAGATTGTGTAATGCTT
A
>MA_LJ_Toh_SA_psbK-psbI(OK350360)
GTTGGCAAGCTGCTGTAAGTTTTCGATAAAGTTATTAAAACTCTACTGAAAAAATTCATGATTTATTTGATAA
AAAAGATTCTAATAAAAATTGATAACGTAATAACAATCTTAGTTTATACATCCTCATAAAAAATATTTGAATT
CTTGTATATTGGATAAAAAGAGCGATAAGTTTGGATCAGTCCATTTCACCGTTCTGGACGCTCTTCCAGTGAG
GAAGTACTTTATTTTTTTATTAGCTTTTGTTTTACACAATACTTTATTGTTATAATTACAGTTAATTTTAGAA
TAAGCTTTTCCGTAATGAACAAGTCATAATCTTAATTTAAAATGCATTCATGAGTTTGAAAATTCAGTTTTTG
TAGAAAAAAAACTTAACTTAAGACTATAAAAAAGAAAGGATTGTTCTTTATTTTTTCATAGTTTTTTCTTGGC
ATGCCAAAATAATATATGTGTTACATAACTCAAATGGATAATCTATTCCCTTTTACCCCAAAAATGATCCTAT
CTTGGAGATTGTGTA
>MA_LJ_AB_SA_psbK-psbI(OK350359)
TTTGGCAAGCTGCTGTAAGTTTTCGATAAAGTTATTAAAACTCTACTGAAAAAATTCATGATTTATTTGATAA
AAAAGATTCTAATAAAAATTGATAACGTAATAACAATCTTAGTTTATACATCCTCATAAAAAATATTTGAATT
CTTGTATATTGGATAAAAAGAGCGATAAGTTTGGATCAGTCCATTTCACCGTTCTGGACGCTCTTCCAGTGAG
GAAGTACTTTATTTTTTTATTAGCTTTTGTTTTACACAATACTTTATTGTTATAATTACAGTTAATTTTAGAA
TAAGCTTTTCCGTAATGAACAAGTCATAATCTTAATTTAAAATGCATTCATGAGTTTGAAAATTCAGTTTTTG
TAGAAAAAAAACTTAACTTAAGACTATAAAAAAGAAAGGATTGTTCTTTATTTTTTCATAGTTTTTTCTTGGC
ATGCCAAAATAATATATGTGTTACATAACTCAAATGGATAATCTATTCCCTTTTACCCCAAAAATGATCCTAT
CTTGGAGATTGTGTAATGCTACCTCCCAAA
>MA_LJ_AT_SA_psbK-psbI(OK546024)
TTTGGCAAGCTGCTGTAAGTTTTCGATAAAGTTATTAAAACTCTACTGAAAAAATTCATGATTTATTTGATAA
AAAAGATTCTAATAAAAATTGATAACGTAATAACAATCTTAGTTTATACATCCTCATAAAAAATATTTGAATT
CTTGTATATTGGATAAAAAGAGCGATAAGTTTGGATCAGTCCATTTCACCGTTCTGGACGCTCTTCCAGTGAG
GAAGTACTTTATTTTTTTATTAGCTTTTGTTTTACACAATACTTTATTGTTATAATTACAGTTAATTTTAGAA
TAAGCTTTTCCGTAATGAACAAGTCATAATCTTAATTTAAAATGCATTCATGAGTTTGAAAATTCAGTTTTTG
TAGAAAAAAAACTTAACTTAAGACTATAAAAAAGAAAGGATTGTTCTTTATTTTTTCATAGTTTTTTCTTGGC
ATGCCAAAATAATATATGTGTTACATAACTCAAATGGATAATCTATTCCCTTTTACCCCAAAAATGATCCTAT
CTTGGAGATTGTGTAATGCTTAT
>MA_LA_J_SA_psbK-psbI(OK350358)
GGCAAGCTGCTGTAAGTTTTCGATAAAGTCATTAAAACTCTACTGAAAAATTCATGATTTATTTGATAAAAAA
AGATTCTAATAATAATTGATAACGTAATAGCAATCTTAGTTTATACATCCTCATAAAAAATAGGTGAATTCTT
GTATATTGGATAAAAAGAGCGCTAAGTTTGGATCTTGCCGTTCTAGCCGCTCTTCCAGTGAGTAACTACTTTA
TTAGCTTTTGTTTTACACAAAACTTTATTGTTATATTATTAGAGTTAATAGTCGAATAACCTTTTGGATAATG
AACAAGTTATAATCTTAATTCAAAAAATTCATGAATTTGAAAATTCAGTTTTTCTAGAAAAAACACTGAATAC
```

TTAATTCAATTAAAAAACAATTGTTCTTTTTTTCACTGTTTTTTTTTATTCGCATGTCAAACTAATACATGTG
TTTACATAACTGAAATGGATAATCTATTCCCTTTTACTCCAAAAATGATCCAATCTTGGAGATTGTGTAATG
>MA_LJ_Ton_SA_psbK-psbI(OK350361)
TGCAAGCTGCTGTAAGTTTTCGATAAAGTTATTAAAACTCTACTGAAAAAATTCATGATTTATTTGATAAAAA
AGATTCTAATAAAAATTGATAACGTAATAACAATCTTAGTTTATACATCCTCATAAAAAATATTTGAATTCTT
GTATATTGGATAAAAAGAGCGATAAGTTTGGATCAGTCCATTTCACCGTTCTGGACGCTCTTCCAGTGAGGAA
GTACTTTATTTTTTTATTAGCTTTTGTTTTACACAATACTTTATTGTTATAATTACAGTTAATTTTAGAATAA
GCTTTTCCGTAATGAACAAGTCATAATCTTAATTTAAAATGCATTCATGAGTTTGAAAATTCAGTTTTTGTAG
AAAAAAAACTTAACTTAAGACTATAAAAAAGAAAGGATTGTTCTTTATTTTTTCATAGTTTTTTCTTGGCATG
CCAAAATAATATATGTGTTACATAACTCAAATGGATAATCTATTCCCTTTTACCCCAAAAATGATCCTATCTT
GGAGATTGTGTAATGCTA
>MA_LP_AQ_SA_psbK-psbI(OK350363)
GTGGCAAGCTTGCTGTAAGTTTTCGATGAAGTTATTAAAACTCTATTGAAAAAATTCATGATTTATTTGATAA
AAAAAGATTCTAATAAAGATAAGATAATATCAATTTTACGTTATACATCCTCATTCCAAAATTTGGAATTCTT
GTATATTGAATAAAAAGAGCGATAAGTTTTGATCAGTCCATTTCGGCCTTCTGGAGGCTCTTCCAGTGAGCAA
GTACTTTATTTATTAGCTTCTGTTTTCCACAATAATTTATTGAATATTAGAGTTAGTATTAGAAAAACCTTTT
GGGTAACGAACAAATCATAATCTTAATTTCAACAACAAAAAATTCATGAATTTTGTAATTCAGTTTTTTTAGA
AAAAAACACTTAATAAAGAAAAAATTGTTCTTTATTTTTACTAGTTTTTTTTGTGGCATGTCAAAATAAGACA
TGTGTTACATAACTCAAATGGATAATCTATTCCCTTTTACCCCAAAAATGATCCTATCTTGGAGATTGTGTAA
TGCT
>MA_LA_M1_SA_psbK-psbI(OK350362)
CTCTACTGAAAAATTCATGATTTATTTGATAAAAAAAGATTCTAATAATAATTGATAACGTAATAGCAATCTT
AGTTTATACATCCTCATAAAAAATAGGTGAATTCTTGTATATTGGATAAAAAGAGCGCTAAGTTTGGATCTTG
CCGTTCTAGCCGCTCTTCCAGTGAGTAACTACTTTATTAGCTTTTGTTTTACACAAAACTTTATTGTTATATT
ATTAGAGTTAATAGTCGAATAACCTTTTGGATAATGAACAAGTTATAATCTTAATTCAAAAAATTCATGAATT
TGAAAATTCAGTTTTTCTAGAAAAAACACTGAATACTTAATTCAATTAAAAAACAATTGTTCTTTTTTTCACT
GTTTTTTTTTATTCGCATGTCAAACTAATACATGTGTTTACATAACTGAAATGGATAATCTATTCCCTTTTAC
TCCAAAAATGATCCAATCTTGGAGATTGTGTAATGCTT
>MA_SP_M2_SA_psbK-psbI(OK546025)
AGCTGCTGTAAGTTTTCGATGAAGTTCTTAATACTCTACTGAAAAAATTCATGATTTTTTTGATAAAAAAGGA
TTCTTATATCTAATAATAATTGATAACATAATATGAGTCTTAGCTTATAAATATAAATCCTCATTAGAAAAGA
AATAAATTTAAATTCTTGTATATTGGATAAAAGGAGCGATAAGTTTGGATCAGTCAATTTCCCCGTTCCGTAC
GCTCTTCCAGTGAGCAAGTACTTTCTTTATTAGCTTATGTTTTTCCACAATACTTTATTGTTAATATTAGAAT
AACCATTTTGGTAACGAACAAATCATAATCTTAATTTCAGAAAAAAAATTCATGAATTTGAAAATTCAGTTTTT
TTAGAAAAAACACTTAATTAAAAAAAATTTGTTCTTTATTTTTTCATATTTTTTTTTTTGTTTTTGGTGGCATGT
CAAAATAATACATGTGTTACATAACTCAAATGGATAATCTATTCCCTTTTACCCCAAAAATGATCCTATCTTG
GAGATTGTGTA
>KJ921758.1
GTTTGGCAAGCTGCTGTAAGTTTTCGATAAAGTTATTAAAACTCTACTGAAAAAATTCATGATTTATTTGATA
AAAAAGATTCTAATAAAAATTGATAACGTAATAACAATCTTAGTTTATACATCCTCATAAAAAATATTTGAAT
TCTTGTATATTGGATAAAAAGAGCGATAAGTTTGGATCAGTCCATTTCACCGTTCTGGACGCTCTTCCAGTGA
GGAAGTACTTTATTTTTTTATTAGCTTTTGTTTTACACAATACTTTATTGTTATAATTACAGTTAATTTTAGA
ATAAGCTTTTCCGTAATGAACAAGTCATAATCTTAATTTAAAATGCATTCATGAGTTTGAAAATTCAGTTTTT
GTAGAAAAAAAACTTAACTTAAGACTATAAAAAAGAAAGGATTGTTCTTTATTTTTTCATAGTTTTTTCTTGG
CATGCCAAAATAATATATGTGTTACATAACTCAAATGGATAATCTATTCCCTTTTACCCCAAAAATGATCCTA
TCTTGGAGATTGTGTAATGCTTACTC
>KJ136047.1
CTCTACTGAAAAATTCATGATTTATTTGATAAAAAAAGATTCTAATAATAATTGATAACGTAATAGCAATCTT
AGTTTATACATCCTCATAAAAAATAGGTGAATTCTTGTATATTGGATAAAAAGAGCGCTAAGTTTGGATCTTG
CCGTTCTAGCCGCTCTTCCAGTGAGTAACTACTTTATTAGCTTTTGTTTTACACAAAACTTTATTGTTATATT
ATTAGAGTTAATAGTCGAATAACCTTTTGGATAATGAACAAGTTATAATCTTAATTCAAAAAATTCATGAATT
TGAAAATTCAGTTTTTCTAGAAAAAACACTGAATACTTAATTCAATTAAAAAACAATTGTTCTTTTTTTCACT
GTTTTTTTTTATTCGCATGTCAAACTAATACATGTGTTTACATAACTGAAATGGATAATCTATTCCCTTTTAC
TCCAAAAATGATCCAATCTTGGAGATTGTGTAATGCTT

```
>GU454305.1
GTAAGTTTTCGATGAAGTTATTAAAACTCTATTGAAAAAATTCATGATTTATTTGATAAAAAAAGATTCTAAT
AAAGATAAGATAATATCAATTTTACGTTATACATCCTCATTCCAAAATTTGGAATTCTTGTATATTGAATAAA
AAGAGCGATAAGTTTTGATCAGTCCATTTCGGCCTTCTGGAGGCTCTTCCAGTGAGCAAGTACTTTATTTATT
AGCTTCTGTTTTCCACAATAATTTATTGAATATTAGAGTTAGTATTAGAAAAACCTTTTGGGTAACGAACAAA
TCATAATCTTAATTTCAACAACAAAAAATTCATGAATTTTGTAATTCAGTTTTTTTAGAAAAAAACACTTAAT
AAAGAAAAAAATTGTTCTTTATTTTTACTAGTTTTTTTTGTGGCATGTCAAAATAAGACATGTGTTACATAACT
CAAATGGATAATCTATTCCC
>GU454300.1
GTAAGTTTTCGATGAAGTTCTTAATACTCTACTGAAAAAATTCATGATTTTTTTGATAAAAAAGGATTCTTAT
ATCTAATAATAATTGATAACATAATATGAGTCTTAGCTTATAAATATAAATCCTCATTAGAAAAGAAATAAAT
TTAAATTCTTGTATATTGGATAAAAGGAGCGATAAGTTTGGATCAGTCAATTTCCCCGTTCCGTACGCTCTTC
CAGTGAGCAAGTACTTTCTTTATTAGCTTATGTTTTTCCACAATACTTTATTGTTAATATTAGAATAACCATT
TTGGTAACGAACAAATCATAATCTTAATTTCAGAAAAAAATTCATGAATTTGAAAATTCAGTTTTTTTAGAAA
AAACACTTAATTAAAAAAATTTGTTCTTTATTTTTTCATATTTTTTTTTTTGTTTTTGGTGGCATGTCAAAATA
ATACATGTGTTACATAACTCAAATGGATAATCTATTCCC
>MA_LJ_R_SA_trnH-psbA(OK103562)
GTTTGGCAAGCTGCTGTAAGTTTTCGATAAAGTTATTAAAACTCTACTGAAAAAATTCATGATTTATTTGATA
AAAAAGATTCTAATAAAAATTGATAACGTAATAACAATCTTAGTTTATACATCCTCATAAAAAATATTTGAAT
TCTTGTATATTGGATAAAAAGAGCGATAAGTTTGGATCAGTCCATTTCACCGTTCTGGACGCTCTTCCAGTGA
GGAAGTACTTTATTTTTTTATTAGCTTTTGTTTTACACAATACTTTATTGTTATAATTACAGTTAATTTTAGA
ATAAGCTTTTCCGTAATGAACAAGTCATAATCTTAATTTAAAATGCATTCATGAGTTTGAAAATTCAGTTTTT
GTAGAAAAAAAACTTAACTTAAGACTATAAAAAAGAAAGGATTGTTCTTTATTTTTTCATAGTTTTTTCTTGG
CATGCCAAAATAATATATGTGTTACATAACTCAAATGGATAATCTATTCCCTTTTACCCCAAAAATGATCCTA
TCTTGGAGATTGTGTAATGCTTACTC
>MG000445.1
AACTCTACTGAAAAAATTCATGATTTATTTGATAAAAAAGATTCTAATAAAAATTGATAACGTAATAACAATC
TTAGTTTATACATCCTCATAAAAAATATTTGAATTCTTGTATATTGGATAAAAAGAGCGATAAGTTTGGATCA
GTCCATTTCACCGTTCTGGACGCTCTTCCAGTGAGGAAGTACTTTATTTTTTTATTAGCTTTTGTTTTACACA
ATACTTTATTGTTATAATTACAGTTAATTTTAGAATAAGCTTTTCCGTAATGAACAAGTCATAATCTTAATTT
AAAATGCATTCATGAGTTTGAAAATTCAGTTTTTGTAGAAAAAAAACTTAACTTAAGACTATAAAAAAGAAAG
GATTGTTCTTTATTTTTTCATAGTTTTTTCTTGGCATGCCAAAATAATATATGTGTTACATAACTCAAATGGA
TAATCTATTCCCTTTTACCCCAAAAATGATCCTATCTTGGAGATTGTGTAATGCTT
>MA_LG_DH_SA_trnH-psbA(OK383787)
ACGTAATGCTCACAACTTCCCTCTAGACTTAGCTGCAGTTGAAGCTCCATCTACCATTGGATAAGACTTTTTG
TCTTAGTGTATAGGGCTTGATGACGGAATAATACCAAACCTCTTTTTTAGAGGTTTGGTATTACTCCTTGCGT
TAATAGAATGGTATTTTGTCTACATAAGTATTTTTTGCATTTGTAGTTTTAGTAAAAAAGAAATAAAAAAGTG
AAATACTTTAGCTAAGCTTAAGTAAGGGGGCGGATGTAGCCAAGTGGATCAAGGCAGTGGATTGTGAATCCAC
C
>MA_LG_TO_SA_TrnH-PsbA(OK383788)
CACAACTTCCCTCTAGACTTAGCTGCAGTTGAAGCTCCATCTACCATTGGATAAGACTTTTTGTCTTAGTGTA
TAGGGCTTGATGACGGAATAATACCAAACCTCTTTTTTAGAGGTTTGGTATTACTCCTTGCGTTAATAGAATG
GTATTTTGTCTACATAAGTATTTTTTGCATTTGTAGTTTTAGTAAAAAAGAAATAAAAAAGTGAAATACTTTA
GCTAAGCTTAAGTAAGGGGGCGGATGTAGCCAAGTGGATCAAGGCAGTGGATTGT
>MA_LG_AB_SA_trnH-PsbA(OK103563)
ATTCACAATCCACTGCCTTGATCCACTTGGCTACATCCGCCCCCTTACTTAAGCTTAGCTAAAGTATTTCACT
TTTTTATTTCTTTTTTACTAAAACTACAAATGCAAAAAATACTTATGTAGACAAAATACCATTCTATTAACGC
AAGGAGTAATACCAAACCTCTAAAAAAGAGGTTTGGTATTATTCCGTCATCAAGCCCTATACACTAAGACAAA
AAGTCTTATCCAATGGTAGATGGAGCTTCAACTGCAGCTAAGTCTAGAGGGAAGTTGTGAGCATTAC
>MA_LA_J_SA_trnH-psbA(OK383786)
TCACAACTTCCCCCTAGAATTAGGGGCAGTTGAAGCTCCATCTACCATTGGATAAGATTTTTTGTCTTAGTGT
ATAGGGCTTTATGAAGGAATAATACCAAACCTCCTTATTAGAGGTTTGGTATTACTCCTTTTCTATGAGTTAG
TACAATGATATTTTGTCTACATAAGTATTTTTTACATTTGTAGTTTTAGTAAAAAAAAAAAAAGAAATAAAAA
AGTTAAATACTTTAGCTAAGCTTAAGTAAGGGGGCGGAGTAGCCAAGTGGATCAAGGCAGTGGATTGTGAT
```

>GU454502.1
ATTCACAATCCACTGCCTTGATCCACTTGGCTACTCCGCCCCCTTACTTAAGCTTAGCTAAAGTATTTAACTT
TTTTATTTCTTTTTTTTTTTTTACTAAAACTACAAATGTAAAAAATACTTATGTAGACAAAATATCATTGTAC
TAACTCATAGAAAAGGAGTAATACCAAACCTCTAATAAGGAGGTTTGGTATTATTCCTTCATAAAGCCCTATA
CACTAAGACAAAAAATCTTATCCAATGGTAGATGGAGCTTCAACTGCCCCTAATTCTAGGGGGAAGTTGTGAG
CATTAC
>MA_LG_AT_SA_trnH-psbA(OK383789)
TGTAATGCTCACAACTTCCCTCTAGACTTAGCTGCAGTTGAAGCTCCATCTACCATTGGATAAGACTTTTTGT
CTTAGTGTATAGGGCTTGATGACGGAATAATACCAAACCTCTTTTTTAGAGGTTTGGTATTACTCCTTGCGTT
AATAGAATGGTATTTTGTCTACATAAGTATTTTTTGCATTTGTAGTTTTAGTAAAAAAGAAATAAAAAAGTGA
AATACTTTAGCTAAGCTTAAGTAAGGGGGCGGATGTAGCCAAGTGGATCAAGGCAGTGGATTGTGA
>MA_LG_TON_SA_trnH-psbA(OK383790)
TGTAATGCTCACAACTTCCCTCTAGACTTAGCTGCAGTTGAAGCTCCATCTACCATTGGATAAGACTTTTTGT
CTTAGTGTATAGGGCTTGATGACGGAATAATACCAAACCTCTTTTTTAGAGGTTTGGTATTACTCCTTGCGTT
AATAGAATGGTATTTTGTCTACATAAGTATTTTTTGCATTTGTAGTTTTAGTAAAAAAGAAATAAAAAAGTGA
AATACTTTAGCTAAGCTTAAGTAAGGGGGCGGATGTAGCCAAGTGGATCAAGGCAGTGGATTGTGA
>MA_LP_AQ_SA_TrnH-psbA(OK247674)
GTAATGCTCACAACTTCCCTCTAGACTTAGCTGCAGTTGAAGCTCCATCTACCATTGGATAAGACTTTTTGTC
TTAGTGTATAGGGCTTGATCAAGGAATAATACCAAACCTCTAATAAAGAGGTTTGGTATTACTCCTTTTGTAT
GAGTTAGTACAATGGTATTTTGTCTACATAAGGATTTTTGACATTTCGAGTTTCAGGCATAAAAGAAATAAAA
AAGTGCAATACTTTAGCTAAGCTTAAGTAAGGGGGCGGATGTAGCCAAGTGGATCAAGGCAGTGGATTGTGA
>GU454494.1
ATTCACAATCCACTGCCTTGATCCACTTGGCTACATCCGCCCCCTTACTTAAGCTTAGCTAAAGTATTGCACT
TTTTTATTTCTTTTATGCCTGAAACTCGAAATGTCAAAAATCCTTATGTAGACAAAATACCATTGTACTAACT
CATACAAAAGGAGTAATACCAAACCTCTTTATTAGAGGTTTGGTATTATTCCTTGATCAAGCCCTATACACTA
AGACAAAAAGTCTTATCCAATGGTAGATGGAGCTTCAACTGCAGCTAAGTCTAGAGGGAAGTTGTGAGCATTA
C
>MA_LE_M1_SA_trnH-psbA(OK571366)
ACTTGGCTACATCCGCCCCCTTACTTAAGCTTAGCTAAAGTATTTAAGTTTTTTATTTCTTTTTTTTTTTTTA
CTAAAACTACAAATGTAAAAAATACTTATGTAGACAAAATACCATTGTACTAACTCATAGAAAAGGAGTAATA
CCAAACCTCTAATAAGGAGGTTTGGTATTATTCCTTCATAAAGCCCTATACACTAAGATAAAAAATCTTATCC
AATGGTAGATGGAGCTTCAA
>MA_SP_M2_SA_trnH-psbA(OK247675)
GAACGTAATGCTCACAACTTCCCTCTAGACTTAGCTGCAGTTGAAGCTCCATCTACCATTGGATAAGACTTTT
TGTCTTAGTGTATAGGGCTTGATAAAGGAATAATACCAAACCTCCTTATTAGAGGTTTGGTATTGCTCCTTTT
GTGTGAATTAGTGCACTCTTATTTTGTCTACATAAGGATTTTTGACATTTGTACTTAGCATACTTTGTATTTT
TGTCAATTTGTAATTAATGTCCATTCTTTCGTTTTGGAATGAAAGAAATCCTTTATTTAATTATATTTACATA
TTTGATATTTACATATTATATAAATATATCTATTATATAAATAAAATCTATAACTATAATATAATTAAATCTT
AAATCTATAATATAATTCAAGGAAAAAAGAAATCCAATTTTAATATTTAAAAAATAAAAGAAACAAAAAAGGT
GAAATACTTTAGCTAAGCTTAAGTAAGGGGGCGGATGTAGCCAAGTGGATCAAGGCAGTGGATTGTGAAT
>GU454491.1
ATTCACAATCCACTGCCTTGATCCACTTGGCTACATCCGCCCCCTTACTTAAGCTTAGCTAAAGTATTTCACC
TTTTTTGTTTCTTTTATTTTTTAAATATTAAAATTGGATTTCTTTTTTCCTTGAATTATATTATAGATTTAAG
ATTTAATTATATTATAGTTATAGATTTTATTTATATAATAGATATATTTATATAATATGTAAATATCAAATAT
GTAAATATAATTAAATAAAGGATTTCTTTCATTCCAAAACGAAAGAATGGACATTAATTACAAATTGACAAAA
ATACAAAGTATGCTAAGTACAAATGTCAAAAATCCTTATGTAGACAAAATAAGAGTGCACTAATTCACACAAA
AGGAGCAATACCAAACCTCTAATAAGGAGGTTTGGTATTATTCCTTTATCAAGCCCTATACACTAAGACAAAA
AGTCTTATCCAATGGTAGATGGAGCTTCAACTGCAGCTMAGTCTAGAGGGAAGTTGTGAGCATTAC
>MA_LG_R_SA_mtk(OK095301)
TGTAATAATGAGAAAGGTTTCTATATATTCTACCAAATCGATCAATAATATCAGAATCTGACAATTCTGCCCA
AGCTGGCTTGCTAATAGGATTCCCCGATAGATTACAAAATTTGGCTTTTGCCAATGACCCAATCATAGGAATA
ATTGATATCATTGTTTCAAATTTTGGAGTAAAGGTATCTATTAAAAAGGCATTCTCTAGCATTTGACTCTTCG
CGGATGAAAGATTTATTGGCACACTTGAAAGATAACCCAAAAAATAGAAAGAAAAATGGGGGAATTGATTTAT
GTGTATCCTACTGGGTTGAGACCAAAAGTGAAAATGACATTGCCATAAATTCACAAAGTAAGATTTCCATTTC

TTCATCAGAAGATGAGTCCCTTTTGAAGCTATAATGCATTTTCCTTGATACCTAACATAATGCATGAAAGGAT
CCTTGAACAACCATAGGGTTTTCTGAAAATCATTACAACAAGTTACTCCAATATGTTTCATTTTTCCATAGAA
GTGTGTTCGCTCAAGAAGGGGTCGAAAAGATGTTGATCGTAAATAAGAGGATGATTTACGAAGAAAATAAAAA
ATGGATTCACATTCGACTACATAAGAATTATATAGGAACCTATAGAGTCTTGGATTATCTTTTGAAAAACCAT
AACTAGATTTATTTGGAGTAATGCTATTACTCCGATTGTGATAGTCGTAAAAAATCAATCGTAATAAATGTAA
AGTGGGAACATCTTGTATCCAGCATTGTAGAATT
>MA_LG_Dh_SA_mtk(OK375248)
ATGGAAGAATTCAAAGGATATTTACAAAAAGGTGGATTTAAACAACAACACTTCCTATATCCACTTCTCTTTC
AAGAGTATATTTATGGACTTGCTCACGATCAGGGTTTAAATGTAAATGCATCAACTTTTAACGAACCCCCTGA
AATTTCAGGTTATGGCAATAAATATAGCTCATTACTTGTGAAACGGTTAATTATTCGAATATACCAACAGAAT
AGTTTTCTCTATTCTGTCAATAATTCTAAGCAAAATAGATTCGTAGGACACAATAAGAATTATTATTATAAAA
TGATATGTGAGGGTTTTTCTATTGTTGTAGAAATTCCCTTTTCACTGCGATTAGTATCTTCTATCAAAGAAAC
AAAAGAAATAATGAAATTTCAGAATTTACGATCTATTCATTCACTATTTCCTTTTTTAGAGGATAAATTTTCA
CATTTAAATTCTGTATCAGATATAGTAATACCTTATCCAATCCATCTAGAAATATTGCTTCAAATTCTACAAT
GCTGGATACAAGATGTTCCCACTTTACATTTATTACGATTGATTTTTTACGACTATCACAATCGGAGTAATAG
CATTACTCCAAATAAATCTAGTTATGGTTTTTCAAAAGATAATCCAAGACTCTATAGGTTCCTATATAATTCT
TATGTAGTCGAATGTGAATCCATTTTTTATTTTCTTCGTAAATCATCCTCTTATTTACGATCAACATCTTTTC
GACCCCTTCTTGAGCGAACACACTTCTATGGAAAAATGAAACATATTGGAGTAACTTGTTGTAATGATTTTCA
GAAAACCCTATGGTTGTTCAAGGATCCTTTCATGCATTATGTTAGGTATCA
>MA_LG_Toh_SA_mtk(OK546020)
TATTTACAAAAAGGTGGATTTAAACAACAACACTTCCTATATCCACTTCTCTTTCAAGAGTATATTTATGGAC
TTGCTCACGATCAGGGTTTAAATGTAAATGCATCAACTTTTAACGAACCCCCTGAAATTTCAGGTTATGGCAA
TAAATATAGCTCATTACTTGTGAAACGGTTAATTATTCGAATATACCAACAGAATAGTTTTCTCTATTCTGTC
AATAATTCTAAGCAAAATAGATTCGTAGGACACAATAAGAATTATTATTATAAAATGATATGTGAGGGTTTTT
CTATTGTTGTAGAAATTCCCTTTTCACTGCGATTAGTATCTTCTATCAAAGAAACAAAAGAAATAATGAAATT
TCAGAATTTACGATCTATTCATTCACTATTTCCTTTTTTAGAGGATAAATTTTCACATTTAAATTCTGTATCA
GATATAGTAATACCTTATCCAATCCATCTAGAAATATTGCTTCAAATTCTACAATGCTGGATACAAGATGTTC
CCACTTTACATTTATTACGATTGATTTTTTACGACTATCACAATCGGAGTAATAGCATTACTCCAAATAAATC
TAGTTATGGTTTTTCAAAAGATAATCCAAGACTCTATAGGTTCCTATATAATTCTTATGTAGTCGAATGTGAA
TCCATTTTTTATTTTCTTCGTAAATCATCCTCTTATTTACGATCAACATCTTTTCGACCCCTTCTTGAGCGAA
CACACTTCTATGGAAAAATGAAACATATTGGAGTAACTTGTTGTAATGATTTTCAGAAAACCCTATGGTTGTT
CAAGGATCCTTTCATGCATTATGTTAGGTA
>MA_LG_AB_SA_Matk(OK571364)
TTCAAGAGTATATTTATGGACTTGCTCACGATCAGGGTTTAAATGTAAATGCATCAACTTTTAACGAACCCCC
TGAAATTTCAGGTTATGGCAATAAATATAGCTCATTACTTGTGAAACGGTTAATTATTCGAATATACCAACAG
AATAGTTTTCTCTATTCTGTCAATAATTCTAAGCAAAATAGATTCGTAGGACACAATAAGAATTATTATTATA
AAATGATATGTGAGGGTTTTTCTATTGTTGTAGAAATTCCCTTTTCACTGCGATTAGTATCTTCTATCAAAGA
AACAAAAGAAATAATGAAATTTCAGAATTTACGATCTATTCATTCACTATTTCCTTTTTTAGAGGATAAATTT
TCACATTTAAATTCTGTATCAGATATAGTAATACCTTATCCAATCCATCTAGAAATATTGCTTCAAATTCTAC
AATGCTGGATACAAGATGTTCCCACTTTACATTTATTACGATTGATTTTTTACGACTATCACAATCGGAGTAA
TAGCATTACTCCAAATAAATCTAGTTATGGTTTTTCAAAAGATAATCCAAGACTCTATAGGTTCCTATATAAT
TCTTATGTAGTCGAATGTGAATCCATTTTTTATTTTCTTCGTAAATCATCCTCTTATTTACGATCAACATCTT
TTCGACCCCTTCTTGAGCGAACACACTTCTATGGAAAAATGAAACATATTGGAGTAACTTGTTGTAATGATTT
TCAGAAAACCCTATGGTTGTTCAAGGATCCTTTCATGCATTATGTTAGGTATCAAGGAAAATGCATTATAGCT
TCAAAAGGGACTCATCTTCTGATGAAGAAATGGAAAT
>AY034197.1
ATGGAAGAATTCAAAGGATATTTACAAAAAGGTGGATTTAAACAACAACACTTCCTATATCCACTTCTCTTTC
AAGAGTATATTTATGGACTTGCTCACGATCAGGGTTTAAATGTAAATGCATCAACTTTTAACGAACCCCCTGA
AATTTCAGGTTATGGCAATAAATATAGCTCATTACTTGTGAAACGGTTAATTATTCGAATATACCAACAGAAT
AGTTTTCTCTATTCTGTCAATAATTCTAAGCAAAATAGATTCGTAGGACACAATAAGAATTATTATTATAAAA
TGATATGTGAGGGTTTTTCTATTGTTGTAGAAATTCCCTTTTCACTGCGATTAGTATCTTCTATCAAAGAAAC
AAAAGAAATAATGAAATTTCAGAATTTACGATCTATTCATTCACTATTTCCTTTTTTAGAGGATAAATTTTCA
CATTTAAATTCTGTATCAGATATAGTAATACCTTATCCAATCCATCTAGAAATATTGCTTCAAATTCTACAAT
GCTGGATACAAGATGTTCCCACTTTACATTTATTACGATTGATTTTTTACGACTATCACAATCGGAGTAATAG

CATTACTCCAAATAAATCTAGTTATGGTTTTTCAAAAGATAATCCAAGACTCTATAGGTTCCTATATAATTCT
TATGTAGTCGAATGTGAATCCATTTTTTATTTTCTTCGTAAATCATCCTCTTATTTACGATCAACATCTTTTC
GACCCCTTCTTGAGCGAACACACTTCTATGGAAAAATGAAACATATTGGAGTAACTTGTTGTAATGATTTTCA
GAAAACCCTATGGTTGTTCAAGGATCCTTTCATGCATTATGTTAGGTATCAAGGAAAATGCATTATAGCTTCA
AAAGGGACTCATCTTCTGATGAAGAAATGGAAATCTTACTTTGTGAATTTATGGCAATGTCATTTTCACTTTT
GGTCTCAACCCAGTAGGATACACATAAATCAATTCCCCCATTTTTCTTTCTATTTTTTGGGTTATCTTTCAAG
TGTGCCAATAAATCTTTCATCCGCGAAGAGTCAAATGCTAGAGAATGCCTTTTTAATAGATACCTTTACTCCA
AAATTTGAAACAATGATATCAATTATTCCTATGATTGGGTCATTGGCAAAAGCCAAATTTTGTAATCTATCGG
GGAATCCTATTAGCAAGCCAGCTTGGGCAGAATTGTCAGATCTGATATTATTGATCGATTTGGTAGAATATAT
AGAAACCTTTCTCATTATTACAGTGGGTCTTCAAAAAAACAGAGTTTGTATCGAATAAAATATATACTTAGAC
TTTCCTGTGCTCGAACTTTGGCTCGTAAACATAAAAGTACAGTACGCGCTTTTTTGCAAAGATTAGGTTCAGA
GTTTTTCGAAGAATTCTTTATGGAAGAAGAAAAAGTACTTTCTTTAATCTTACCAAGAACTTATTATCCTTTA
CATCAGTTATCTAGAGAACCCATTTGGTATTTGGATATTATTCGGATAAATGACTTGGTAAATCATTTCGATT
TATGA
>MA_LA_J_SA_mtk(OK375249)
ATGGAAGAATTCAAAGGATATTTACAAAAAGGTGGATTTAAACAACAACACTTCCTATATCCACTTCTCTTTC
AAGAGTATATTTATGCACTTGCTCATGATCAGGGTTTAAATGTAAATGCATCAACTTTTACTGAACCCCCTGA
AATTTCAGGTTATCACAATAAATATAGCTCATTACTTGTGAAACGGTTAATTAAGCGAATATACCAACAGAAT
CGTTTTATCTATTCTGTTACTAATTCTAAGCAAAATAGATTTGTTGGACACAACAATCATTTTTATTCTCAAA
TGATATCAGAGGGTTTTTCTAGTGTTGTCGAAATTCCGTTTTCACCGCGCTTAGTATCTTCTCTCAAAAAAAA
AAAAGAAATACCGAAATATCAGAATTTACGATCTATTCATTCACTATTTCCATTTTTAGAGGATAAATTTACA
CATTTAACTTATGTATCAGATATACTGATACCCTATCCGGTTCATCTAGAAATATTGGTTCAAATTCTACAAT
GCTGGATACAAGATGTTCCCACTTTACATTTATTACGATTGCTTTTTCACGAATATCATAACGGGAATAATAG
CATTACTCCAAATAAATCTACTTATGGTTTTTCAAAAGATAATCCAAGACTCTATAGGTTCCTATATAATTCT
TATGTAGTTGAATGCGAATCCATATTTCTTTTTCTTCGTAAATCATCCTCTTATTTACGATCAAGATCTTTTG
GACCCCTTCTTGAGCGAACACAGTTCTATGGAAAAATGAAACATATTGGAGTAACTTGTTGTAATGATTTTCA
TAAAAGCCTATGTTTATTCAAGGATCCTTTCATGCATTATG
>OK375249.1
ATGGAAGAATTCAAAGGATATTTACAAAAAGGTGGATTTAAACAACAACACTTCCTATATCCACTTCTCTTTC
AAGAGTATATTTATGCACTTGCTCATGATCAGGGTTTAAATGTAAATGCATCAACTTTTACTGAACCCCCTGA
AATTTCAGGTTATCACAATAAATATAGCTCATTACTTGTGAAACGGTTAATTAAGCGAATATACCAACAGAAT
CGTTTTATCTATTCTGTTACTAATTCTAAGCAAAATAGATTTGTTGGACACAACAATCATTTTTATTCTCAAA
TGATATCAGAGGGTTTTTCTAGTGTTGTCGAAATTCCGTTTTCACCGCGCTTAGTATCTTCTCTCAAAAAAAA
AAAAGAAATACCGAAATATCAGAATTTACGATCTATTCATTCACTATTTCCATTTTTAGAGGATAAATTTACA
CATTTAACTTATGTATCAGATATACTGATACCCTATCCGGTTCATCTAGAAATATTGGTTCAAATTCTACAAT
GCTGGATACAAGATGTTCCCACTTTACATTTATTACGATTGCTTTTTCACGAATATCATAACGGGAATAATAG
CATTACTCCAAATAAATCTACTTATGGTTTTTCAAAAGATAATCCAAGACTCTATAGGTTCCTATATAATTCT
TATGTAGTTGAATGCGAATCCATATTTCTTTTTCTTCGTAAATCATCCTCTTATTTACGATCAAGATCTTTTG
GACCCCTTCTTGAGCGAACACAGTTCTATGGAAAAATGAAACATATTGGAGTAACTTGTTGTAATGATTTTCA
TAAAAGCCTATGTTTATTCAAGGATCCTTTCATGCATTATG
>MA_LG_AT_SA_mtk(OK546021)
TTCAAGAGTATATTTATGGACTTGCTCACGATCAGGGTTTAAATGTAAATGCATCAACTTTTAACGAACCCCC
TGAAATTTCAGGTTATGGCAATAAATATAGCTCATTACTTGTGAAACGGTTAATTATTCGAATATACCAACAG
AATAGTTTTCTCTATTCTGTCAATAATTCTAAGCAAAATAGATTCGTAGGACACAATAAGAATTATTATTATA
AAATGATATGTGAGGGTTTTTCTATTGTTGTAGAAATTCCCTTTTCACTGCGATTAGTATCTTCTATCAAAGA
AACAAAAGAAATAATGAAATTTCAGAATTTACGATCTATTCATTCACTATTTCCTTTTTTAGAGGATAAATTT
TCACATTTAAATTCTGTATCAGATATAGTAATACCTTATCCAATCCATCTAGAAATATTGCTTCAAATTCTAC
AATGCTGGATACAAGATGTTCCCACTTTACATTTATTACGATTGATTTTTTACGACTATCACAATCGGAGTAA
TAGCATTACTCCAAATAAATCTAGTTATGGTTTTTCAAAAGATAATCCAAGACTCTATAGGTTCCTATATAAT
TCTTATGTAGTCGAATGTGAATCCATTTTTTATTTTCTTCGTAAATCATCCTCTTATTTACGATCAACATCTT
TTCGACCCCTTCTTGAGCGAACACACTTCTATGGAAAAATGAAACATATTGGAGTAACTTGTTGTAATGATTT
TCAGAAAACCCTATGGTTGTTCAAGGATCCTTTCATGCAT
>MA_LG_Ton_SA_mtk(OK546026)

GGTGGATTTAAACAACAACACTTCCTATATCCACTTCTCTTTCAAGAGTATATTTATGGACTTGCTCACGATC
AGGGTTTAAATGTAAATGCATCAACTTTTAACGAACCCCCTGAAATTTCAGGTTATGGCAATAAATATAGCTC
ATTACTTGTGAAACGGTTAATTATTCGAATATACCAACAGAATAGTTTTCTCTATTCTGTCAATAATTCTAAG
CAAAATAGATTCGTAGGACACAATAAGAATTATTATTATAAAATGATATGTGAGGGTTTTTCTATTGTTGTAG
AAATTCCCTTTTCACTGCGATTAGTATCTTCTATCAAAGAAACAAAAGAAATAATGAAATTTCAGAATTTACG
ATCTATTCATTCACTATTTCCTTTTTTAGAGGATAAATTTTCACATTTAAATTCTGTATCAGATATAGTAATA
CCTTATCCAATCCATCTAGAAATATTGCTTCAAATTCTACAATGCTGGATACAAGATGTTCCCACTTTACATT
TATTACGATTGATTTTTTACGACTATCACAATCGGAGTAATAGCATTACTCCAAATAAATCTAGTTATGGTTT
TTCAAAAGATAATCCAAGACTCTATAGGTTCCTATATAATTCTTATGTAGTCGAATGTGAATCCATTTTTTAT
TTTCTTCGTAAATCATCCTCTTATTTACGATCAACATCTTTTCGACCCCTTCTTGAGCGAACACACTTCTATG
GAAAAATGAAACATATTGGAGTAACTTGTTGTAATGATTTTCAGAAAACCCTATGGTTGTTCAAGGATCCTTT
CATGCATTATGTTAGGTATCAAGGAAAATGCATTATA
>MA_LP_AQ_SA_mtk(OK375250)
GGTTAATTACTCGAATATACCAACAGAATAGTTTGATCCATTCTGTTAATGATTCTAAGCAAAATAGATTCAT
TGGACACAACAAGAATTTTTATTATCAAATGATATCCGAGGGTTTTGCTATCGTAGTAGAAATTCCGTTTTCA
ATGCGATTAGTATCTTCTCTCAAAAAAAAAGAAATACCGAAATATCAGAATTTACGATCTATTCATTCAATAT
TCCCATTTTTAGAGGATAAATTTGCACATTTAAATTATGTATCAGATATACTGATACCTTATCCGGTTCATCT
AGAAATATTGGTTCAAATTCTACAATGCTGGGTACAGGATGTTCCCGCTTTACATTTATTACGATTGCTTTTT
CATGACTATCATAATGGGAGTAATTGCATTACTCCAAAAAAATCTAGTTATGGTTTTTCAAAAGATAATCCAA
GACTCTATAGGTTCCTATATAATTCTTATGTAGTCGAATGCGAATCCATATTTGTTTTTCTTCGTAAATCATC
CTCTTATTTACGATCAACATCTTTTGGATCCCTTCTTGAGCGAACACACTTCTATGGAAAAATGAAACATATT
GGAGTAACTTGTTGTAATGATTTTCAGAAAACCCTATGGTTGTTCAAGGATCCTTTCATGCATTATGTTAGGT
ATCAAGGAAAATCCATTATGGCTTCAAAAGGGACTCATCTTCTGATGAAGAAATGGAAATCTTACTTTGTGAA
TTTATGGCAATGTCATTTTCACTTTTGGTCTCAACCCAGTA
>KX526519.1
GCATCAACTTTTAATGAACCCGCTGAAATTTCCGGTTATGACAATAAATATAGCTCATTACTTGTGAAACGGT
TAATTACTCGAATATACCAACAGAATAGTTTGATCCATTCTGTTAATGATTCTAAGCAAAATAGATTCATTGG
ACACAACAAGAATTTTTATTATCAAATGATATCCGAGGGTTTTGCTATCGTAGTAGAAATTCCGTTTTCAATG
CGATTAGTATCTTCTCTCAAAAAAAAAGAAATACCGAAATATCAGAATTTACGATCTATTCATTCAATATTCC
CATTTTTAGAGGATAAATTTGCACATTTAAATTATGTATCAGATATACTGATACCTTATCCGGTTCATCTAGA
AATATTGGTTCAAATTCTACAATGCTGGGTACAGGATGTTCCCGCTTTACATTTATTACGATTGCTTTTTCAT
GACTATCATAATGGGAGTAATTGCATTACTCCAAAAAAATCTAGTTATGGTTTTTCAAAAGATAATCCAAGAC
TCTATAGGTTCCTATATAATTCTTATGTAGTCGAATGCGAATCCATATTTGTTTTTCTTCGTAAATCATCCTC
TTATTTACGATCAACATCTTTTGGATCCCTTCTTGAGCGAACACACTTCTATGGAAAAATGAAACATATTGGA
GTAACTTGTTGTAATGATTTTCAGAAAACCCTATGGTTGTTCAAGGATCCTTTCATGCATTATGTTAGGTATC
AAGGAAAATCCATTATGGCTTCAAAAGGGACTCATCTTCTGATGAAGAAATGGAAATCTTACTTTGTGAATTT
ATGGCAATGTCATTTTCACTTTTGGTCTCAACCCAGTAGGATACACATAAACCAATTCCCCCATTTTTCTTTC
TATTTTTTGGGTTATCTTTCAAGTGTACCAATAAATCCTTCATCCGTGAAGAGTCAAATGCTAGAGAATTCTT
TTTTAATAGATACCGTTACTCCAAAATTTGAAACGATGATATCAATTATTCCTATGATTGGGTCATTGGCAAA
AGCTAAATTTTGTAATCTATCGGGGAATCCTATTAGCAAGCCAGTTTGGGCCGATTTGTCAGATTCTGATATT
ATTGATCGATTTGGTAGAATATGTAGAAACCTTTCTCATTATTACAGTGGGTCTTCAAAAAAACAGAGTTTAT
ATCGAATAAAGTATATACTTAGACTTTCATGTGCTCGAACTTTGGCGCGTAAACATAAAAGTACAGTACGTGC
TTTTTTGCAAAGATTAGGTTCAGAGTTTTTCGAAGAATTCTTTATGGAAGAAGAAAAAGTTCTTTCTTTAATT
TTACCAAGAACTTCTT
ATCCTTTACATCAGTTATATAGAGAACCTATTTGGTATTTGGATATTGTTCGTATAAATGACTTGGTGAATCA
T
>MA_LA_M1_SA_mtk(OK546022)
CGAATATCATAACGGGAATAATAGCATTACTCCAAATAAATCTACTTATGGTTTTTCAAAAGATAATCCAAGA
CTCTATAGGTTCCTATATAATTCTTATGTAGTTGAATGCGAATCCATATTTCTTTTTCTTCGTAAATCATCCT
CTTATTTACGATCAACATCTTTTGGACCCCTTCTTGAGCGAACACAGTTCTATGGAAAAATGAAACATATTGG
AGTAACTTGTTGTAATGATTTTCAGAAAACCCTATGTTTATTCAAGGATCCTTTCATGCATTATGTTAGGTAT
CAAGGAAAATCCATTATGGCTTCAAAAGGGACTGATTTCCTGATGAAAAAATGGAAATCTTACTTTGTGAATT
TATGGCAATATCGTTTTCACTTTTGGTCTGAACCTAGTAGGATACACATAAACCAATTCCCCCATTTTTCTTT
CTATTTTTTAGGTTATCTTTCAAGTGTACCAATAAATCTTTCATACGCGAAGAGTCAAATGCTAGAGAATTCG

TTTTTAATAGAAACCTTTACTCAAAAATTTGAAACGATGATATCCATTATTCCTATGATTGGATCATTGGCAA
AAGCTAAATTTTGTAATCTATCGGGGAATCCTATTAGCAAGCCAGCTTGGGCTGATTTGTCAGATTCGGATAT
TATTGATCGATTTGGTAGAATATATAGAAACCTTTCTCATTATTACAGTGGATCTTCAAAAAAACAAACTTTG
TATCGAATAAAGTATATACTTAGGCTTTCATGTGCTAGAACTTTGGCTCGTAA
>KP017666.1
ATGTTCCCACTTTACATTTATTACGATTGCTTTTTCACGAATATCATAACGGGAATAATAGCATTACTCCAAA
TAAATCTACTTATGGTTTTTCAAAAGATAATCCAAGACTCTATAGGTTCCTATATAATTCTTATGTAGTTGAA
TGCGAATCCATATTTCTTTTTCTTCGTAAATCATCCTCTTATTTACGATCAACATCTTTTGGACCCCTTCTTG
AGCGAACACAGTTCTATGGAAAAATGAAACATATTGGAGTAACTTGTTGTAATGATTTTCAGAAAACCCTATG
TTTATTCAAGGATCCTTTCATGCATTATGTTAGGTATCAAGGAAAATCCATTATGGCTTCAAAAGGACTGAT
TTCCTGATGAAAAAATGGAAATCTTACTTTGTGAATTTATGGCAATATCGTTTTCACTTTTGGTCTGAACCTA
GTAGGATACACATAAACCAATTCCCCCATTTTTCTTTCTATTTTTTAGGTTATCTTTCAAGTGTACCAATAAA
TCTTTCATACGCGAAGAGTCAAATGCTAGAGAATTCGTTTTTAATAGAAACCTTTACTCAAAAATTTGAAACG
ATGATATCCATTATTCCTATGATTGGATCATTGGCAAAAGCTAAATTTTGTAATCTATCGGGGAATCCTATTA
GCAAGCCAGCTTGGGCTGATTTGTCAGATTCGGATATTATTGATCGATTTGGTAGAATATATAGAAACCTTTC
TCATTATTACAGTGGATCTTCAAAAAAACAAACTTTGTATCGAATAAAGTATATACTTAGGCTTTCATGTGCT
AGAACTTTGGCTCGTAA
>MA_LM_R_SA_rpoC1 (OK376426)
TCCCCTTTCTCGGTCCGAGAAGTGTATTGTAGGAACCGGGCTGGAACGCCAAGCGGCTTTAGATTCTGGCGTT
TCAGTTATAGCCGAACACGAGGGAAAAGTCATTTCTACTGATACTCACCAAATTGTTTTCTCAGGTAATGGAA
ACACTCTAAATATTCCATTAGTTATGTATCAACGTTCCAACAAAAATACTTGTATGCACCAAAAACCTCAGGT
TCAACGGGGTAAATACGTTAAAAAGGGACAAATTTTAGCTGACGGGGCGGCTACCGTTGGTGGAGAACTCGCT
TTGGGAAAAAAATGTATTAGTAGCTTATATGCCGTGGGAAGGCTACAATTTTGAAGATGCGGTACTTATTAGTG
AGCGTTTGGTATATAGCGATATTTAT
>MA_LG_Dh_SA_rpoC1 (Ok546019)
TTTTTATGCAGCGTCAAGCAGTTCCCCTTTCTCGGTCCGAGAAGTGTATTGTAGGAACCGGGCTGGAACGCCA
AGCGGCTTTAGATTCTGGCGTTTCAGTTATAGCCGAACACGAGGGAAAAGTCATTTCTACTGATACTCACCAA
ATTGTTTTCTCAGGTAATGGAAACACTCTAAATATTCCATTAGTTATGTATCAACGTTCCAACAAAAATACTT
GTATGCACCAAAAACCTCAGGTTCAACGGGGTAAATACGTTAAAAAGGGACAAATTTTAGCTGACGGGGCGGC
TACCGTTGGTGGAGAACTCGCTTTGGGAAAAAAATGTATTAGTAGCTTATATGCCGTGGGAAGGCTACAATTTT
GAAGATGCGGTACTTATTAGTGAGCGTTTGGTATATAGCGATATTTATACTTCTTTTCACAT
>MA_LM_To_SA_rpoC1 (OK376427)
CAGTTCCCCTTTCTCGGTCCGAGAAGTGTATTGTAGGAACCGGGCTGGAACGCCAAGCGGCTTTAGATTCTGG
CGTTTCAGTTATAGCCGAACACGAGGGAAAAGTCATTTCTACTGATACTCACCAAATTGTTTTCTCAGGTAAT
GGAAACACTCTAAATATTCCATTAGTTATGTATCAACGTTCCAACAAAAATACTTGTATGCACCAAAAACCTC
AGGTTCAACGGGGTAAATACGTTAAAAAGGGACAAATTTTAGCTGACGGGGCGGCTACCGTTGGTGGAGAACT
CGCTTTGGGAAAAAAATGTATTAGTAGCTTATATGCCGTGGGAAGGCTACAATTTTGAAGATGCGGTACTTATT
AGTGAGCGTTTGGTATATAGCGATATTTATACTTCTTTTCACATCCGAAAA
>MA_LM_AB_SA_rpoC1(OK493446)
TGAGCAGTTCCCCTTTCTCGGTCCGAGAAGTGTATTGTAGGAACCGGGCTGGAACGCCAAGCGGCTTTAGATT
CTGGCGTTTCAGTTATAGCCGAACACGAGGGAAAAGTCATTTCTACTGATACTCACCAAATTGTTTTCTCAGG
TAATGGAAACACTCTAAATATTCCATTAGTTATGTATCAACGTTCCAACAAAAATACTTGTATGCACCAAAAA
CCTCAGGTTCAACGGGGTAAATACGTTAAAAAGGGACAAATTTTAGCTGACGGGGCGGCTACCGTTGGTGGAG
AACTCGCTTTGGGAAAAAAATGTATTAGTAGCTTATATGCCGTGGGAAGGCTACAATTTTGAAGATGCGGTACT
TATTAGTGAGCGTTTGGTATATAGCGATATTTATACTTCTTTTCACATCCGAA
>KF726306.1
AAGCAGTTCCCCTTTCTCGGTCCGAGAAGTGTATTGTAGGAACCGGGCTGGAACGCCAAGCGGCTTTAGATTC
TGGCGTTTCAGTTATAGCCGAACACGAGGGAAAAGTCATTTCTACTGATACTCACCAAATTGTTTTCTCAGGT
AATGGAAACACTCTAAATATTCCATTAGTTATGTATCAACGTTCCAACAAAAATACTTGTATGCACCAAAAAC
CTCAGGTTCAACGGGGTAAATACGTTAAAAAGGGACAAATTTTAGCTGACGGGGCGGCTACCGTTGGTGGAGA
ACTCGCTTTGGGAAAAAAATGTATTAGTAGCTTATATGCCGTGGGAAGGCTACAATTTTGAAGATGCGGTACTT
ATTAGTGAGCGTTTGGTATATAGCGATATTTATACTTCTTTTCACATCCGAA
>MA_LA_J_SA_rpoC1(OK493447)

AGGCGGTCAGCAGTTCCCCTTTCTCGGTCCGAGAAGTGTATTGTAGGAACCGGACTGGAACGCCAAGCGGCTT
TAGATTCTGGCGTTTCAGTTATAGCCGAACACGAGGGAAAAGTCATTTCTACTGATACTCACCAAATTGTTTT
CTCAAGTAATGGCAAGACTCTAAATATTCCATTAGTTATGTATCAACGTTCCAACAAAAATACTTGTATGCAC
CAAAAACCACAGGTTAAACGGGGTAAATACGTTAAAAAGGGACAAATTTTAGCTGACGGGGCGGCTACCGTTG
GTGGAGAACTCGCTTTGGGAAAAAAATGTCTTAGTAGCTTATATGCCATGGGAAGGCTACAATTTTGAAGATGC
GGTACTTATTAGTGAGCGTTTGGTATATAGCGATATTTATACTTCTTTTCA
>KP017715.1
CAGCAGTTCCCCTTTCTCGGTCCGAGAAGTGTATTGTAGGAACCGGACTGGAACGCCAAGCGGCTTTAGATTC
TGGCGTTTCAGTTATAGCCGAACACGAGGGAAAAGTCATTTCTACTGATACTCACCAAATTGTTTTCTCAAGT
AATGGCAAGACTCTAAATATTCCATTAGTTATGTATCAACGTTCCAACAAAAATACTTGTATGCACCAAAAAC
CACAGGTTAAACGGGGTAAATACGTTAAAAAGGGACAAATTTTAGCTGACGGGGCGGCTACCGTTGGTGGAGA
ACTCGCTTTGGGAAAAAAATGTCTTAGTAGCTTATATGCCATGGGAAGGCTACAATTTTGAAGATGCGGTACTT
ATTAGTGAGCGTTTGGTATATAGCGATATTTATACTTCTTTTCACATCCG
>MA_LG_AT_SA_rpoC1(OK493448)
CAGTTCCCCTTTCTCGGTCCGAGAAGTGTATTGTAGGAACCGGGCTGGAACGCCAAGCGGCTTTAGATTCTGG
CGTTTCAGTTATAGCCGAACACGAGGGAAAAGTCATTTCTACTGATACTCACCAAATTGTTTTCTCAGGTAAT
GGAAACACTCTAAATATTCCATTAGTTATGTATCAACGTTCCAACAAAAATACTTGTATGCACCAAAAACCTC
AAGTTCAACGGGGTAAATACGTTAAAAAGGGACAAATTTTAGCTGACGGGGCGGCTACCGTTGGTGGAGAACT
CGCTTTGGGAAAAAAATGTATTAGTAGCTTATATGCCATGGGAAGGCTACAATTTTGAAGATGCGGTACTTATT
AGTGAGCGTTTGGTATATAGCGATATTTGGGCTCCTTTTCCA
>MA_LM_Ton_SA_rpoC1(OK493449)
CAGCAGTTCCCCTTTCTCGGTCCGAGAAGTGTATTGTAGGAACCGGGCTGGAACGCCAAGCGGCTTTAGATTC
TGGCGTTTCAGTTATAGCCGAACACGAGGGAAAAGTCATTTCTACTGATACTCACCAAATTGTTTTCTCAGGT
AATGGAAACACTCTAAATATTCCATTAGTTATGTATCAACGTTCCAACAAAAATACTTGTATGCACCAAAAAC
CTCAGGTTCAACGGGGTAAATACGTTAAAAAGGGACAAATTTTAGCTGACGGGGCGGCTACCGTTGGTGGAGA
ACTCGCTTTGGGAAAAAAATGTATTAGTAGCTTATATGCCGTGGGAAGGCTACAATTTTGAAGATGCGGTACTT
ATTAGTGAGCGTTTGGTATATAGCGATATTTATACTTCTTTTCAC
>MA_LP_AQ_SA_rpoC1(OK493450)
TTTTTGTTGGAGGGGTCAAGCAGTTCCGCTTTCTCGGTCCGAGAAGTGCATTGTAGGAACCGGATTGGAACGC
CAAGCGGCTTTAGATTCTGGAGTTTCAGTTATAGCCGAACACGAGGGAAAAGTCATTTCTACTGATACTCACC
GAATTGTTTTCTCAGGTAATGGAAACACTCTAAATATTCCATTAGTTATGTATCAACGTTCCAACAAAAATAC
TTGTATGCACCAAAAACCTCAGGTTCAACGTGGTAAATACGTTAAAAAGGGACAAATTTTGGCCGACGGGGCG
GCTACCGTTGGTGGAGAACTCGCTTTGGGAAAAAAATGTATTAGTAGCTTATATGCCATGGGAAGGCTACAATT
TTGAAGATGCGGTACTTATTAGTGAGCGTTTGGTATATAGCGATATTTATACTTCTTTTCACAAACCCA
>KP017720.1
AAGCAGTTCCGCTTTCTCGGTCCGAGAAGTGCATTGTAGGAACCGGATTGGAACGCCAAGCGGCTTTAGATTC
TGGAGTTTCAGTTATAGCCGAACACGAGGGAAAAGTCATTTCTACTGATACTCACCGAATTGTTTTCTCAGGT
AATGGAAACACTCTAAATATTCCATTAGTTATGTATCAACGTTCCAACAAAAATACTTGTATGCACCAAAAAC
CTCAGGTTCAACGTGGTAAATACGTTAAAAAGGGACAAATTTTGGCCGACGGGGCGGCTACCGTTGGTGGAGA
ACTCGCTTTGGGAAAAAAATGTATTAGTAGCTTATATGCCATGGGAAGGCTACAATTTTGAAGATGCGGTACTT
ATTAGTGAGCGTTTGGTATATAGCGATATTTATACTTCTTTTCACATCCG
>MA_LE_M1_SA_rpoC1(OK493451)
CAGTTCCCCTTTCTCGGTCCGAGAAGTGTATTGTAGGAACCGGACTGGAACGCCAAGCGGCTTTAGATTCTGG
CGTTTCAGTTATAGCCGAACACGAGGGAAAAGTCATTTCTACTGATACTCACCAAATTGTTTTCTCAAGTAAT
GGCAAGACTCTAAATATTCCATTAGTTATGTATCAACGTTCCAACAAAAATACTTGTATGCACCAAAAACCAC
AGGTTAAACGGGGTAAATACGTTAAAAAGGGACAAATTTTAGCTGACGGGGCGGCTACCGTTGGTGGAGAACT
CGCTTTGGGAAAAAAATGTCTTAGTAGCTTATATGCCATGGGAAGGCTACAATTTTGAAGATGCGGTACTTATT
AGTGAGCGTTTGGTATATAGCGATATTTATACTTCTTTTCA
>MA_SP_M2_SA_rpoC1(OK493452)
TTTTTTATGGGAGGGGGTCAAGCAGTTCCCCTTTCTCGGTCCGAGAAGTGCATTGTAGGAACCGGACTGGAAC
GCCAAGCGGCTTTAGATTCTGGAGTTTCAGTTATAGCCGAACACGAGGGAAAGGTCATTTCTACTGATACTCA
CCAAATTGTTTTCTCAGGTAATGGAAACACTGTAAATATTCCATTAGTTATGTATGAACGTTCCAACAAAAAT
ACTTGTATGCACCAAAAACCTCAGGTTCAACGGGGTAAATACATTAAAAAGGGACAAATTTTAGCCGACGGGG

CGGCTACTGTTGGTGGAGAACTCGCTTTGGGAAAAAATGTATTAGTAGCTTATATGCCATGGGAAGGCTACAA
TTTTGAAGATGCGGTACTTATTAGTGAGCGTTTGGTATATAGCGATATTTATACTTCTTTC
>KP017721.1
AAGCAGTTCCCCTTTCTCGGTCCGAGAAGTGCATTGTAGGAACCGGACTGGAACGCCAAGCGGCTTTAGATTC
TGGAGTTTCAGTTATAGCCGAACACGAGGGAAAGGTCATTTCTACTGATACTCACCAAATTGTTTTCTCAGGT
AATGGAAACACTGTAAATATTCCATTAGTTATGTATGAACGTTCCAACAAAAATACTTGTATGCACCAAAAAC
CTCAGGTTCAACGGGGTAAATACATTAAAAAGGGACAAATTTTAGCCGACGGGGCGGCTACTGTTGGTGGAGA
ACTCGCTTTGGGAAAAAATGTATTAGTAGCTTATATGCCATGGGAAGGCTACAATTTTGAAGATGCGGTACTT
ATTAGTGAGCGTTTGGTATATAGCGATATTTATACTTCTTTCCACATCCG
>MA_LG_R_SA_atpFH-atpH(OK095300)
TTTCCAAAATAAATTACTACACCAAGTACTACGCTTAGATTTATTGGATTTGTTGCTAAAATATCGGTATTAA
ACCCAAAACCACCGGCGGATGGCCAGTGGCCCAAGGAAACAAAAGAATCAGTTACATTTTGCATATACTCTCC
TCTTATAGATAGGACTAAAAAAGAACAGAGTTCTGTTCTTTTTGTATTACTTCGCCCCCTTTGTTTGATTTCT
TTTTTTTTATGGGATTTTAAAATGGAATAGATTAAATTAATTAATTTAATTGAGAACTTTTTTATTTATTATT
TAATTCTAATTAAAGTTTACAATTACAAGAACATACTTATTGGGTTAGGTCCTGGCTATTTTGTCAATTGGTA
AATACCTTGTTTATTGCGTTACAACGCATACTCTAAAAAATTTTGCATTACATTATACTAAGAACTGAAAACG
GGAAGGAAGAAAGCGAGAGGATCTGCTAATTACTAATCCTAAAATCAGTCCTTCCCGGAGGTATTCTCTCAAC
GAATAAGTAATTGTTAGAGTACAATGTTGATATAATTCGAAGAAGCAAAAAGCAAGTCTAAGTCAAAAAGTCT
ATTACGTACTTTTTTTGATTCTAGAATTAAACAAATGGATTCGCAAATAAAAGTGCTAATGCCACAACCAGTC
CATAAATTGTTAAAGCTTCCATAAAAGC
>MA_LG_Dh_SA_atpFH-atpH (OK383787)
ACTCGCACACACTCCCTTTCCAAAATAAATTACTACACCAAGTACTACGCTTAGATTTATTGGATTTGTTGCT
AAAATATCGGTATTAAACCCAAAACCACCGGCGGATGGCCAGTGGCCCAAGGAAACAAAAGAATCAGTTACAT
TTTGCATATACTCTCCTCTTATAGATAGGACTAAAAAAGAACAGAGTTCTGTTCTTTTTGTATTACTTCGCCC
CCTTTGTTTGATTTCTTTTTTTTTTATGGGATTTTAAAATGGAATAGATTAAATTAATTAATTTAATTGAGAAC
TTTTTTATTTATTATTTAATTCTAATTAAAGTTTACAATTACAAGAACATACTTATTGGGTTAGGTCCTGGCT
ATTTTGTCAATTGGTAAATACCTTGTTTATTGCGTTACAACGCATACTCTAAAAAATTTTGCATTACATTATA
CTAAGAACTGAAAACGGGAAGGAAGAAAGCGAGAGGATCTGCTAATTACTAATCCTAAAATCAGTCCTTCCCG
GAGGTATTCTCTCAACGAATAAGTAATTGTTAGAGTACAATGTTGATATAATTCGAAGAAGCAAAAAGCAAGT
CTAAGTCAAAAAGTCTATTACGTACTTTTTTTGATTCTAGAATTAAACAAATGGATTCGCAAATAAAAGTGCT
AATGCCACAACCAGTCCATAAATTGTTAAAGCTTCCATAAAAGC
>MA_LG_Toh_SA_atpFH-atpH (OK598959)
CCTTTCCAAAATAAATTACTACACCAAGTACTACGCTTAGATTTATTGGATTTGTTGCTAAAATATCGGTATT
AAACCCAAAACCACCGGCGGATGGCCAGTGGCCCAAGGAAACAAAAGAATCAGTTACATTTTGCATATACTCT
CCTCTTATAGATAGGACTAAAAAAGAACAGAGTTCTGTTCTTTTTGTATTACTTCGCCCCCTTTGTTTGATTT
CTTTTTTTTTTATGGGATTTTAAAATGGAATAGATTAAATTAATTAATTTAATTGAGAACTTTTTTATTTATTA
TTTAATTCTAATTAAAGTTTACAATTACAAGAACATACTTATTGGGTTAGGTCCTGGCTATTTTGTCAATTGG
TAAATACCTTGTTTATTGCGTTACAACGCATACTCTAAAAAATTTTGCATTACATTATACTAAGAACTGAAAA
CGGGAAGGAAGAAAGCGAGAGGATCTGCTAATTACTAATCCTAAAATCAGTCCTTCCCGGAGGTATTCTCTCA
ACGAATAAGTAATTGTTAGAGTACAATGTTGATATAATTCGAAGAAGCAAAAAGCAAGTCTAAGTCAAAAAGT
CTATTACGTACTTTTTTTGATTCTAGAATTAAACAAATGGATTCGCAAATAAAAGTGCTAATGCCACAACCAG
TC
>KX212889.1
ACTCGCACACACTCCCTTTCCAAAATAAATTACTACACCAAGTACTACGCTTAGATTTATTGGATTTGTTGCT
AAAATATCGGTATTAAACCCAAAACCACCGGCGGATGGCCAGTGGCCCAAGGAAACAAAAGAATCAGTTACAT
TTTGCATATACTCTCCTCTTATAGATAGGACTAAAAAAGAACAGAGTTCTGTTCTTTTTGTATTACTTCGCCC
CCTTTGTTTGATTTCTTTTTTTTTTATGGGATTTTAAAATGGAATAGATTAAATTAATTAATTTAATTGAGAAC
TTTTTTATTTATTATTTAATTCTAATTAAAGTTTACAATTACAAGAACATACTTATTGGGTTAGGTCCTGGCT
ATTTTGTCAATTGGTAAATACCTTGTTTATTGCGTTACAACGCATACTCTAAAAAATTTTGCATTACATTATA
CTAAGAACTGAAAACGGGAAGGAAGAAAGCGAGAGGATCTGCTAATTACTAATCCTAAAATCAGTCCTTCCCG
GAGGTATTCTCTCAACGAATAAGTAATTGTTAGAGTACAATGTTGATATAATTCGAAGAAGCAAAAAGCAAGT
CTAAGTCAAAAAGTCTATTACGTACTTTTTTTGATTCTAGAATTAAACAAATGGATTCGCAAATAAAAGTGCT
AATGCCACAACCAGTCCATAAATTGTTAAAGCTTCCATAAAAG
>MA_LG_AT_SA_atpFH-atpH (OK598958)

CAAAATAAATTACTACACCAAGTACTACGCTTAGATTTATTGGATTTGTTGCTAAAATATCGGTATTAAACCC
AAAACCACCGGCGGATGGCCAGTGGCCCAAGGAAACAAAAGAATCAGTTACATTTTGCATATACTCTCCTCTT
ATAGATAGGACTAAAAAAGAACAGAGTTCTGTTCTTTTTGTATTACTTCGCCCCCTTTGTTTGATTTCTTTTT
TTTTATGGGATTTTAAAATGGAATAGATTAAATTAATTAATTTAATTGAGAACTTTTTTATTTATTATTTAAT
TCTAATTAAAGTTTACAATTACAAGAACATACTTATTGGGTTAGGTCCTGGCTATTTTGTCAATTGGTAAATA
CCTTGTTTATTGCGTTACAACGCATACTCTAAAAAATTTTGCATTACATTATACTAAGAACTGAAAACGGGAA
GGAAGAAAGCGAGAGGATCTGCTAATTACTAATCCTAAAATCAGTCCTTCCCGGAGGTATTCTCTCAACGAAT
AAGTAATTGTTAGAGTACAATGTTGATATAATTCGAAGAAGCAAAAAGCAAGTCTAAGTCAAAAAGTCTATTA
CGTACTTTTTTTGATTCTAGAATTAAACAAATGGATTCGCAAATAAAAGTGCTAATGCCACAACCAGTCCATA
AATTGTTGAACTGCTGTAAA
>MA_LG_Ton_SA_atpFH-atpH (OK598957)
CTCCTTTCCAAAATAAATTACTACACCAAGTACTACGCTTAGATTTATTGGATTTGTTGCTAAAATATCGGTA
TTAAACCCAAAACCACCGGCGGATGGCCAGTGGCCCAAGGAAACAAAAGAATCAGTTACATTTTGCATATACT
CTCCTCTTATAGATAGGACTAAAAAAGAACAGAGTTCTGTTCTTTTTGTATTACTTCGCCCCCTTTGTTTGAT
TTCTTTTTTTTTATGGGATTTTAAAATGGAATAGATTAAATTAATTAATTTAATTGAGAACTTTTTTATTTAT
TATTTAATTCTAATTAAAGTTTACAATTACAAGAACATACTTATTGGGTTAGGTCCTGGCTATTTTGTCAATT
GGTAAATACCTTGTTTATTGCGTTACAACGCATACTCTAAAAAATTTTGCATTACATTATACTAAGAACTGAA
AACGGGAAGGAAGAAAGCGAGAGGATCTGCTAATTACTAATCCTAAAATCAGTCCTTCCCGGAGGTATTCTCT
CAACGAATAAGTAATTGTTAGAGTACAATGTTGATATAATTCGAAGAAGCAAAAAGCAAGTCTAAGTCAAAAA
GTCTATTACGTACTTT
>MA_LA_J_SA_atpFH-atpH (OK598956)
CAAAATAAATCCCTACCCCAAGTACTACGCTTAGATTTATTGGATTTGTTGCTAAAATATCGGTATTAAACCC
AAAACCCCCGGCAGACGGCCAGTGGCCCAAGGAAACAAAAGAATCAGTTACATTTTGCATATACTTTCCTCTT
ATAGATAGGACTAAAAAAGAACAGAGTTCTTTTTGTATTACTTCGCCCCTTTTCTTTGATTTCTTTTTTTTTT
TATGGGATTTTAAAATAGAATAGATGACATTAATTAACTTAATTGAGAACTTTTTTATTTATTATTTTATTCT
AATTAAAGTTTACAATTACAAGAGCATACTTATTGGGTTAGGGCCTGACTATTTTGTCAATAAATACCTTGTT
TGTTGCGTTAGAACGCATACTCAAAAAAGTTTTCCCTTACATTATACTAAGAACTAAAAACGGGAAGGAAGAA
AGCGAGAGGATCTGCTAATTACTAATCCTAAAATCAGTCCTTCCCGGAGGTATTCTCTCAACGAATAAGTAAT
TGTTAGAGTGCAATGTTGATAGAATTCGAAGAAGCAAAAAGCAAGTCTAAGTCAAAAAGTACTTTCTTTTTTG
TAGAATTAAACAAATGGATTCGCAAATAAAAGTGCTAATGCCACAACCAGTCCATAAATTGACA
>KJ630548.1
ACTCGCACACACTCCCTTTCCAAAATAAATCCCTACCCCAAGTACTACGCTTAGATTTATTGGATTTGTTGCT
AAAATATCGGTATTAAACCCAAAACCCCCGGCAGACGGCCAGTGGCCCAAGGAAACAAAAGAATCAGTTACAT
TTTGCATATACTTTCCTCTTATAGATAGGACTAAAAAAGAACAGAGTTCTTTTTGTATTACTTCGCCCCTTTT
CTTTGATTTCTTTTTTTTTTTTATGGGATTTTAAAATAGAATAGATGACATTAATTAACTTAATTGAGAACTTT
TTTATTTATTATTTTATTCTAATTAAAGTTTACAATTACAAGAGCATACTTATTGGGTTAGGGCCTGACTATT
TTGTCAATAAATACCTTGTTTGTTGCGTTAGAACGCATACTCAAAAAAGTTTTCCCTTACATTATACTAAGAA
CTAAAAACGGGAAGGAAGAAAGCGAGAGGATCTGCTAATTACTAATCCTAAAATCAGTCCTTCCCGGAGGTAT
TCTCTCAACGAATAAGTAATTGTTAGAGTGCAATGTTGATAGAATTCGAAGAAGCAAAAAGCAAGTCTAAGTC
AAAAAGTACTTTCTTTTTTGTAGAATTAAACAAATGGATTCGCAAATAAAAGTGCTAATGCCACAACCAGTCC
ATAAATTGTTAAAGCTTCCATAAAAGC
>MA_LP_AQ_SA_atpFH-atpH (OK598955)
TCGCACACACTCCCTTTCCAAAATAAATCACTACACCAAGTACTACGCTTAGATTTATTGGATTTGTTGCTAA
AATATCGGTATTAAACCCAAAACCCACGGCGGATGGCCCCTGGGCCAAGGAAACAAAAGAATCAGTTACATTT
TGCATATACTCTCCTCTTATAGATAGGACTAACAAAGAACAGAGTTCTTTTTGTATTACTTCGCCCCCTTTGG
TTGATTTCTTTTTTTTTTATGGTATGGGATTTTAAAATGGAATATATTGAATTAATTAATTGAGAACTTTTTA
ATTTATTTATTATTTTATAATAATTAAATATTCTAAATTAAATATTCTAATTAAATTAAAGTTTACAATTACA
AGAAGATACTTATTGGGTTAGGTCCTGGGTATTTTGTCAATTGATAAATACCTTGTTTGTTGCGTTGCAACGC
ATACTAAAAAAAGGTTTCCATTATACTAAGAACTAAAAACGGGAAGGAAGAAAGCGAGAGGATCTCCTAATTA
CTAATCCTAAAATCAGTCCTTCCCGGAGGTATTCTATCAACTAATAAGTAATTGTTAGAGTGCAATGTTGATA
TAATTCGAAGAAGCAGAAAACAAGTCTAAGTAAAAAAAGTCTATTATGTACTTTTTTCTTATAGAATTAAACA
AATGGATTCGCAAATAAAAGTGCTAATGCCACGACCAGTCCATAAATTGTTAAAGCTTCCATAAAAGC
>KJ630555.1

ACTCGCACACACTCCCTTTCCAAAATAAATCACTACACCAAGTACTACGCTTAGATTTATTGGATTTGTTGCT
AAAATATCGGTATTAAACCCAAAACCCACGGCGGATGGCCCCTGGGCCAAGGAAACAAAAGAATCAGTTACAT
TTTGCATATACTCTCCTCTTATAGATAGGACTAACAAAGAACAGAGTTCTTTTTGTATTACTTCGCCCCCTTT
GGTTGATTTCTTTTTTTTTTATGGTATGGGATTTTAAAATGGAATATATTGAATTAATTAATTGAGAACTTTT
TAATTTATTTATTATTTTATAATAATTAAATATTCTAAATTAAATATTCTAATTAAATTAAAGTTTACAATTA
CAAGAAGATACTTATTGGGTTAGGTCCTGGGTATTTTGTCAATTGATAAATACCTTGTTTGTTGCGTTGCAAC
GCATACTAAAAAAAGGTTTCCATTATACTAAGAACTAAAAACGGGAAGGAAGAAAGCGAGAGGATCTCCTAAT
TACTAATCCTAAAATCAGTCCTTCCCGGAGGTATTCTATCAACTAATAAGTAATTGTTAGAGTGCAATGTTGA
TATAATTCGAAGAAGCAGAAAACAAGTCTAAGTAAAAAAAGTCTATTATGTACTTTTTTCTTATAGAATTAAA
CAAATGGATTCGCAAATAAAAGTGCTAATGCCACGACCAGTCCATAAATTGTTAAAGCTTCCATAAAAGC
>MA_LA_M1_SA_atpFH-atpH (OK598954)
CGCACACACTCCCTTTCCAAAATAAATCCCTACCCCAAGTACTACGCTTAGATTTATTGGATTTGTTGCTAAA
ATATCGGTATTAAACCCAAAACCCCCGGCAGACGGCCAGTGGCCCAAGGAAACAAAAGAATCAGTTACATTTT
GCATATACTTTCCTCTTATAGATAGGACTAAAAAAGAACAGAGTTCTTTTTGTATTACTTCGCCCCTTTTCTT
TGATTTCTTTTTTTTTTTATGGGATTTTAAAATAGAATAGATGACATTAATTAACTTAATTGAGAACTTTTTT
ATTTATTATTTTATTCTAATTAAAGTTTACAATTACAAGAGCATACTTATTGGGTTAGGGCCTGACTATTTTG
TCAATAAATACCTTGTTTGTTGCGTTAGAACGCATACTCAAAAAAGTTTTCCCTTACATTATACTAAGAACTA
AAAACGGGAAGGAAGAAAGCGAGAGGATCTGCTAATTACTAATCCTAAAATCAGTCCTTCCCGGAGGTATTCT
CTCAACGAATAAGTAATTGTTAGAGTGCAATGTTGATAGAATTCGAAGAAGCAAAAAGCAAGTCTAAGTCAAA
AAGTACTTTCTTTTTTGTAGAATTAAACAAATGGATTCGCAAATAAAAGTGCTAATGCCACAACCAGTCCA
>MA_SP_M2_SA_atpFH-atpH(OK247676)
CCTTTCCAAAATAAATCAATACACCAAGTACTACGCTTAGATTTATTGGATTTGTTGCTAAAATATCGGTATT
AAACCCAAAACCCCCGGCGGATGGCCAGTGGCCCAAGGAAACAAAAGAATCAGTTACATTTTTCATATACTCT
CCTCTTATAGATAGGACTAACAAAGAACAGAGTTCTTTTTGTATCACTTCGCCCCCTTTTTTTGGTTGATTTC
TTTTTTTTTTATGGGATTTTTTAATGGAATAGATTAAATCAATTAATTTAATTGAGAACTTTTTAATTTATAA
TTTTATTCTAATTAAATTAAAGTTTACAAGAAGATACTTATTGGGTTAGGTCCTGGGTATTTTGTCAATTGAT
AAATACCTTGTTTGTTGCGTTGCAACGCATACAAAAAAAGGTTTCCGTTACATTATACTAAGAACTAAAAACG
GGAAGGAAGAAAGCGAGAGGATCTGCTAATTACTAATCCTAAAATCAGTCCTTCCCGGAGGTATTCTCTCAAC
GAATAAGTAATTGTTAGAGTGCAATGTTGATATAATTCGAAGAAACAAAAAGCAAGTCTAAGTCAAAAAAAAT
TACGTACGTACTTTTATTTCTTCTAAAATTAAATTAAACAAATGGATTCGCAAATAAAAGTGCTAATGCCACA
ACCAGTCCATAAATTGTT
>KJ630513.1
ACTCGCACACACTCCCTTTCCAAAATAAATCAATACACCAAGTACTACGCTTAGATTTATTGGATTTGTTGCT
AAAATATCGGTATTAAACCCAAAACCCCCGGCGGATGGCCAGTGGCCCAAGGAAACAAAAGAATCAGTTACAT
TTTTCATATACTCTCCTCTTATAGATAGGACTAACAAAGAACAGAGTTCTTTTTGTATCACTTCGCCCCCTTT
TTTTGGTTGATTTCTTTTTTTTTTATGGGATTTTTTAATGGAATAGATTAAATCAATTAATTTAATTGAGAAC
TTTTTAATTTATAATTTTATTCTAATTAAATTAAAGTTTACAAGAAGATACTTATTGGGTTAGGTCCTGGGTA
TTTTGTCAATTGATAAATACCTTGTTTGTTGCGTTGCAACGCATACAAAAAAAGGTTTCCGTTACATTATACT
AAGAACTAAAAACGGGAAGGAAGAAAGCGAGAGGATCTGCTAATTACTAATCCTAAAATCAGTCCTTCCCGGA
GGTATTCTCTCAACGAATAAGTAATTGTTAGAGTGCAATGTTGATATAATTCGAAGAAACAAAAAGCAAGTCT
AAGTCAAAAAAAATTACGTACGTACTTTTATTTCTTCTAAAATTAAATTAAACAAATGGATTCGCAAATAAAA
GTGCTAATGCCACAACCAGTCCATAAATTGTTAAAGCTTCCATAAAAGC
>MA_LG_R_SA_rbcl (OK571367)
TATACTCCTGAGTATGAGACAAAAGATACGGATATCTTGGCAGCATTCCGAGTAACTCCTCAACCTGGAGTTC
CACCTGAAGAAGCAGGGGCTGCAGTAGCTGCCGAATCTTCTACTGGTACATGGACAACTGTGTGGACTGATGG
ACTTACCAGCCTTGATCGTTACAAAGGACGATGCTACCATATCGAACCTGTTGCTGGAGAAGAAAATCAATTT
ATTGCTTATGTAGCTTACCCATTAGACCTTTTTGAAGAAGGTTCTGTTACTAACATGTTTACCTCCATTGTAG
GTAACGTATTTGGGTTTAAAGCTTTACGAGCTCTACGTTTGGAAGATTTGCGAATTCCTCCTGCGTATTCCAA
AACTTTCCAAGGCCCACCTCATGGGATCCAAGTTGAGAGAGATAAATTGAACAAGTATGGTCGTCCTCTATTG
GGATGTACCATCAAACCAAAATTGGGATTATCCGCGAAAAACTACGGTAGAGCGGTTTATGAATGTCTACGTG
GTGGACTTGATTTTACCAAGGATGATGAAAACGTAAACTCACAACCATTTATGCGTTGG
>MA_LG_DH_SA_rbcl (OK598946)
GATAATCCCAATTTTGGTTTGATGGTACATCCCAATAGAGGACGACCATACTTGTTCAATTTATCTCTCTCAA
CTTGGATCCCATGAGGTGGGCCTTGGAAAGTTTTGGAATACGCAGGAGGAATTCGCAAATCTTCCAAACGTAG

AGCTCGTAAAGCTTTAAACCCAAATACGTTACCTACAATGGAGGTAAACATGTTAGTAACAGAACCTTCTTCA
AAAAGGTCTAATGGGTAAGCTACATAAGCAATAAATTGATTTTCTTCTCCAGCAACAGGTTCGATATGGTAGC
ATCGTCCTTTGTAACGATCAAGGCTGGTAAGTCCATCAGTCCACACAGTTGTCCATGTACCAGTAGAAGATTC
GGCAGCTACTGCAGCCCCTGCTTCTTCAGGTGGAACTCCAGGTTGAGGAGTTACTCGGAATGCTGCCAAGATA
TCCGTATCTTTTGTCTCATACTCAGGAGTATAATAAGTCAATTTGTAATCTTTAACACCAGCTTTGAATCCAG
CACTTGCTTTAGTTTCTGT
>MA_LG_Toh_SA_rbcl (OK598947)
CCACCACGTAGACATTCATAAACCGCTCTACCGTAGTTTTTCGCGGATAATCCCAATTTTGGTTTGATGGTAC
ATCCCAATAGAGGACGACCATACTTGTTCAATTTATCTCTCTCAACTTGGATCCCATGAGGTGGGCCTTGGAA
AGTTTTGGAATACGCAGGAGGAATTCGCAAATCTTCCAAACGTAGAGCTCGTAAAGCTTTAAACCCAAATACG
TTACCTACAATGGAGGTAAACATGTTAGTAACAGAACCTTCTTCAAAAAGGTCTAATGGGTAAGCTACATAAG
CAATAAATTGATTTTCTTCTCCAGCAACAGGTTCGATATGGTAGCATCGTCCTTTGTAACGATCAAGGCTGGT
AAGTCCATCAGTCCACACAGTTGTCCATGTACCAGTAGAAGATTCGGCAGCTACTGCAGCCCCTGCTTCTTCA
GGTGGAACTCCAGGTTGAGGAGTTACTCGGAATGCTGCCAAGATATCCGTATCTTTTGTCTCATACTCAGGAG
TATAATAAGTCAATTTGTAATCTTTAACACCAGCTTTGAATCCAGCACTTGCTTTAGTTTCTGTT
>MA_LG_AB_SA_rbcl (OK598949)
CAAAAGATACGGATATCTTGGCAGCATTCCGAGTAACTCCTCAACCTGGAGTTCCACCTGAAGAAGCAGGGGC
TGCAGTAGCTGCCGAATCTTCTACTGGTACATGGACAACTGTGTGGACTGATGGACTTACCAGCCTTGATCGT
TACAAAGGACGATGCTACCATATCGAACCTGTTGCTGGAGAAGAAAATCAATTTATTGCTTATGTAGCTTACC
CATTAGACCTTTTTGAAGAAGGTTCTGTTACTAACATGTTTACCTCCATTGTAGGTAACGTATTTGGGTTTAA
AGCTTTACGAGCTCTACGTTTGGAAGATTTGCGAATTCCTCCTGCGTATTCCAAAACTTTCCAAGGCCCACCT
CATGGGATCCAAGTTGAGAGAGATAAATTGAACAAGTATGGTCGTCCTCTATTGGGATGTACCATCAAACCAA
AATTGGGATTATCCGCGAAAAACTACGGTAGAGCGGTTTATGAATGTCTACGTGGTGGACTTGATTTTACCAA
GGATGATGAAAACGTAAACTCACAACCATTTATGCGTTGGAGAGATCGT
>JN114815.1
GAAACTAAAGCAAGTGCTGGATTCAAAGCTGGTGTTAAAGATTACAAATTGACTTATTATACTCCTGAGTATG
AGACAAAAGATACGGATATCTTGGCAGCATTCCGAGTAACTCCTCAACCTGGAGTTCCACCTGAAGAAGCAGG
GGCTGCAGTAGCTGCCGAATCTTCTACTGGTACATGGACAACTGTGTGGACTGATGGACTTACCAGCCTTGAT
CGTTACAAAGGACGATGCTACCATATCGAACCTGTTGCTGGAGAAGAAAATCAATTTATTGCTTATGTAGCTT
ACCCATTAGACCTTTTTGAAGAAGGTTCTGTTACTAACATGTTTACCTCCATTGTAGGTAACGTATTTGGGTT
TAAAGCTTTACGAGCTCTACGTTTGGAAGATTTGCGAATTCCTCCTGCGTATTCCAAAACTTTCCAAGGCCCA
CCTCATGGGATCCAAGTTGAGAGAGATAAATTGAACAAGTATGGTCGTCCTCTATTGGGATGTACCATCAAAC
CAAAATTGGGATTATCCGCGAAAAACTACGGTAGAGCGGTTTATGAATGTCTACGTGGTGGACTTGATTTTAC
CAAGGATGATGAAAACGTAAACTCACAACCATTTATGCGTTGGAGAGATCGT
>MA_LM_J_SA_rbcl (OK598948)
CTGAAGAAGCAGGTGCTGCAGTAGCTGCCGAATCTTCTACTGGTACATGGACAACTGTGTGGACTGATGGACT
TACCAGCCTTGATCGTTACAAAGGACGATGCTACCATATCGAACCCGTTGCTGGAGAAGAAAATCAATATATT
GCTTATGTAGCTTACCCATTAGACCTTTTTGAAGAAGGTTCTGTTACTAACATGTTTACCTCCATTGTAGGTA
ACGTATTTGGGTTTAAAGCTTTACGAGCTCTACGTCTGGAAGATTTGAGAATTCCTCCTGCTTATTCCAAAAC
TTTCCAAGGCCCACCTCATGGTATCCAAGTTGAGAGAGATAAATTGAACAAGTATGGTCGTCCTCTATTGGGA
TGTACCATCAAACCAAAATTGGGATTATCCGCGAAAAACTACGGTAGAGCGGTTTATGAATGTCTACGTGGTG
GACTTGATTTTACCAAGGATGATGAAAACGTAAACTCACAACCATTTATGCGTTGGAGAGACCGTTTCCTATT
TTGTGCGGAAGCAATTTATAAATCACAAGCCGAAACAGGTGAAATTAAAGGGCATTACCTAAAT
>GQ436374.1
TAAAGCAAGTGCTGGATTCAAAGCTGGTGTTAAAGATTACAAATTGACTTATTATACTCCTGAGTATGAGACA
AAAGATACGGATATCTTGGCAGCATTCCGAGTAACTCCTCAACCTGGAGTTCCACCTGAAGAAGCAGGTGCTG
CAGTAGCTGCCGAATCTTCTACTGGTACATGGACAACTGTGTGGACTGATGGACTTACCAGCCTTGATCGTTA
CAAAGGACGATGCTACCATATCGAACCCGTTGCTGGAGAAGAAAATCAATATATTGCTTATGTAGCTTACCCA
TTAGACCTTTTTGAAGAAGGTTCTGTTACTAACATGTTTACCTCCATTGTAGGTAACGTATTTGGGTTTAAAG
CTTTACGAGCTCTACGTCTGGAAGATTTGAGAATTCCTCCTGCTTATTCCAAAACTTTCCAAGGCCCACCTCA
TGGTATCCAAGTTGAGAGAGATAAATTGAACAAGTATGGTCGTCCTCTATTGGGATGTACCATCAAACCAAAA
TTGGGATTATCCGCGAAAAACTACGGTAGAGCGGTTTATGAATGTCTACGTGGTGGACTTGATTTTACCAAGG
ATGATGAAAACGTAAACTCACAACCATTTATGCGTTGGAGAGACCGTTTCCTATTTTGTGCGGAAGCAATTTA
TAAATCACAAGCCGAAACAGGTGAAATTAAAGGGCATTACCTAAAT

```
>MA_LG_AT_SA_rbcl (OK598951)
TATACTCCTGAGTATGAGACAAAAGATACGGATATCTTGGCAGCATTCCGAGTAACTCCTCAACCTGGAGTTC
CACCTGAAGAAGCAGGGGCTGCAGTAGCTGCCGAATCTTCTACTGGTACATGGACAACTGTGTGGACTGATGG
ACTTACCAGCCTTGATCGTTACAAAGGACGATGCTACCATATCGAACCTGTTGCTGGAGAAGAAAATCAATTT
ATTGCTTATGTAGCTTACCCATTAGACCTTTTTGAAGAAGGTTCTGTTACTAACATGTTTACCTCCATTGTAG
GTAACGTATTTGGGTTTAAAGCTTTACGAGCTCTACGTTTGGAAGATTTGCGAATTCCTCCTGCGTATTCCAA
AACTTTCCAAGGCCCACCTCATGGGATCCAAGTTGAGAGAGATAAATTGAACAAGTATGGTCGTCCTCTATTG
GGATGTACCATCAAACCAAAATTGGGATTATCCGCGAAAAACTACGGTAGAGCGGTTTATGAATGTCTACGTG
GTGGACTTGATTTTACCAAGGATGATGAAAACGTAAACTCACAACCATTTATGCGTTGGAGAGATCGT
>MA_LG_Ton_SA_rbcl (OK598952)
AAAAGATACGGATATCTTGGCAGCATTCCGAGTAACTCCTCAACCTGGAGTTCCACCTGAAGAAGCAGGGGCT
GCAGTAGCTGCCGAATCTTCTACTGGTACATGGACAACTGTGTGGACTGATGGACTTACCAGCCTTGATCGTT
ACAAAGGACGATGCTACCATATCGAACCTGTTGCTGGAGAAGAAAATCAATTTATTGCTTATGTAGCTTACCC
ATTAGACCTTTTTGAAGAAGGTTCTGTTACTAACATGTTTACCTCCATTGTAGGTAACGTATTTGGGTTTAAA
GCTTTACGAGCTCTACGTTTGGAAGATTTGCGAATTCCTCCTGCGTATTCCAAAACTTTCCAAGGCCCACCTC
ATGGGATCCAAGTTGAGAGAGATAAATTGAACAAGTATGGTCGTCCTCTATTGGGATGTACCATCAAACCAAA
ATTGGGATTATCCGCGAAAAACTACGGTAGAGCGGTTTATGAATGTCTACGTGGTGGACTTGATTTTACCAAG
GATGATGAAAACGTAAACTCACAACCATTTATGCGTTGGAGAGATCGT
>MA_LP_AQ_SA_rbcl(OK571368)
ACCTAGACATTCATAAACCGCTCTACCGTAGTTTTTCGCGGATAATCCCAATTTTGGTTTGATGGTACATCCC
AATAGAGGACGACCATACTTGTTCAATTTATCTCTCTCAACTTGGATCCCATGAGGTGGGCCTAGGAAAGTTT
TGGAATAAGCAGGAGGAATTCGCAAATCTTCCAGACGTAGAGCTCGTAAAGCTTTAAACCCAAATACATTACC
TACAATGGAAGTAAACATGTTAGTAACAGAACCTTCTTCAAAAAGGTCTAATGGGTACGCTACATAAGCAATA
TATTGATTTTCTTCTCCAGCAACGGGTTCGATATGGTAGCATCGTCCTTTGTAACGATCAAGGCTGGTAAGTC
CATCAGTCCACACAGTTGTCCATGTACCGGTAGAAGATTCGGCAGCTACTGCAGCCCCTGCTTCTTCAGCAGG
AACTCCAGGTTGAGGAGTTACTCGGAATGCTGCCAAGATATCCGTATCTTTTGTCTCATACTCAGGAGTATAA
TAAGTCAATTTGTAATCTTTAACACCAGCTTTGAATCCAGCACTTGCTTTAGTCTCTGT
>AY034223.1
AAGTGCTGGATTCAAAGCTGGTGTTAAAGATTACAAATTGACTTATTATACTCCTGAGTATGAGACAAAAGAT
ACGGATATCTTGGCAGCATTCCGAGTAACTCCTCAACCTGGAGTTCCTGCTGAAGAAGCAGGGGCTGCAGTAG
CTGCCGAATCTTCTACCGGTACATGGACAACTGTGTGGACTGATGGACTTACCAGCCTTGATCGTTACAAAGG
ACGATGCTACCATATCGAACCCGTTGCTGGAGAAGAAAATCAATATATTGCTTATGTAGCGTACCCATTAGAC
CTTTTTGAAGAAGGTTCTGTTACTAACATGTTTACTTCCATTGTAGGTAATGTATTTGGGTTTAAAGCTTTAC
GAGCTCTACGTCTGGAAGATTTGCGAATTCCTCCTGCTTATTCCAAAACTTTCCTAGGCCCACCTCATGGGAT
CCAAGTTGAGAGAGATAAATTGAACAAGTATGGTCGTCCTCTATTGGGATGTACCATCAAACCAAAATTGGGA
TTATCCGCGAAAAACTACGGTAGAGCGGTTTATGAATGTCTACGTGGTGGACTTGATTTTACCAAGGATGATG
AAAACGTAAACTCACAACCATTTATGCGTTGGAGAGACCGTTTCTTATTTTGTGCTGAAGCAATTTATAAAGC
ACAAGCTGAAACAGGTGAAATTAAAGGGCATTACCTAAATGCTACTGCAGGTAATTGTGAAGACATGATGAAA
AGGGCTGTGTTTGCTAGAGAATTGGCAGTACCTATTGTAATGCATGACTACTTAACAGGTGGATTCACTGCAA
ATACTAGTTTAGCAGATTATTGCCGAGACAACGGCCTACTTCTTCACATCCACCGCGCAATGCATGCAGTTAT
TGATAGACAGAAAAATCATGGTATGCATTTCCGTGTACTAGCTAAAGCATTACGTATGTCTGGTGGGGATCAT
ATTCACGCTGGTACAGTAGTAGGTAAACTGGAAGGTGAACGTGAGATGACTTTAGGTTTTGTTGATTTATTAC
GTGATGATTTCATTGAAAAAGACAGAAGTCGTGGTATTTTCTTCACTCAAGATTGGGTCTCTATGCCAGGTGT
TCTGCCTGTAGCTTCCGGGGGTATTCATGTTTGGCATATGCCTGCCCTGACCGAGATCTTTGGAGATGATTCC
GTACTACAGTTTGGTGGCGGAACTTTAGGACACCCTTGGGGAAATGCACCTGGTGCAGTAGCTAACCGTGTAG
CTTTAGAAGCGTGTGTACAAGCTCGTAATGAGGGACGTGACCTTGCTCGTGAAGGTAATGAAATTATCCGTGA
AGCTTGCAAATGGAGTCCTGAATTAGCCGCTGCT
>MA_LM_M1_SA_rbcl(OK571369)
TCCAAGTCCACCACGTAGACATTCATAAACCGCTCTACCGTAGTTTTTCGCGGATAATCCCAATTTTGGTTTG
ATGGTACATCCCAATAGAGGACGACCATACTTGTTCAATTTATCTCTCTCAACTTGGATACCATGAGGTGGGC
CTTGGAAAGTTTTGGAATAAGCAGGAGGAATTCTCAAATCTTCCAGACGTAGAGCTCGTAAAGCTTTAAACCC
AAATACGTTACCTACAATGGAGGTAAACATGTTAGTAACAGAACCTTCTTCAAAAAGGTCTAATGGGTAAGCT
ACATAAGCAATATATTGATTTTCTTCTCCAGCAACGGGTTCGATATGGTAGCATCGTCCTTTGTAACGATCAA
GGCTGGTAAGTCCATCAGTCCACACAGTTGTCCATGTACCAGTAGAAGATTCGGCAGCTACTGCAGCACCTGC
```

TTCTTCAGGTGGAACTCCAGGTTGAGGAGTTACTCGGAATGCTGCCAAGATATCCGTATCTTTTGTCTCATAC
TCAGGAGTATAATAAGTCAATTTGTAATCTTTAACACCAGCTTTGAATCCAGCACTTGCTTTAGTCTCTG
>MA_SP_M2_SA_rbcl (OK598950)
ATTATACTCCTGAGTATGAGACAAAAGATACGGATATCTTGGCAGCATTCCGAGTAACTCCTCAACCTGGAGT
TCCACCTGAAGAAGCAGGGGCTGCAGTAGCTGCCGAATCTTCTACTGGTACATGGACAACTGTGTGGACTGAT
GGACTTACCAGCCTTGATCGTTACAAAGGACGATGCTACCATATCGAACCCGTTGTTGGAGAGGAAAATCAAT
ATATTGCTTATGTAGCTTACCCTTTAGACCTTTTTGAAGAAGGTTCTGTTACTAACATGTTTACTTCCATTGT
AGGTAATGTATTTGGGTTTAAAGCTTTACGAGCTCTACGTCTGGAAGATTTGCGAATTCCTCCTGCTTATTCC
AAAACTTTCCAAGGCCCACCTCATGGGATCCAAGTTGAGAGAGATAAATTGAACAAGTATGGTCGTCCTCTAT
TGGGATGTACCATCAAACCAAAATTGGGATTATCCGCGAAAAACTACGGTAGAGCGGTTTATGAATGTCTACG
TGGTGGACTTGATTTTACCAAGGATGATGAAAACGTGAACTCACAACCATTTATGCGTTGGAG
>KC584885.1
ATGTCACCACAAACAGAGACTAAAGCAAGTGTTGGATTCAAAGCTGGTGTTAAAGATTACAAATTGACTTATT
ATACTCCTGAGTATGAGACAAAAGATACGGATATCTTGGCAGCATTCCGAGTAACTCCTCAACCTGGAGTTCC
ACCTGAAGAAGCAGGGGCTGCAGTAGCTGCCGAATCTTCTACTGGTACATGGACAACTGTGTGGACTGATGGA
CTTACCAGCCTTGATCGTTACAAAGGACGATGCTACCATATCGAACCCGTTGTTGGAGAGGAAAATCAATATA
TTGCTTATGTAGCTTACCCTTTAGACCTTTTTGAAGAAGGTTCTGTTACTAACATGTTTACTTCCATTGTAGG
TAATGTATTTGGGTTTAAAGCTTTACGAGCTCTACGTCTGGAAGATTTGCGAATTCCTCCTGCTTATTCCAAA
ACTTTCCAAGGCCCACCTCATGGGATCCAAGTTGAGAGAGATAAATTGAACAAGTATGGTCGTCCTCTATTGG
GATGTACCATCAAACCAAAATTGGGATTATCCGCGAAAAACTACGGTAGAGCGGTTTATGAATGTCTACGTGG
TGGACTTGATTTTACCAAGGATGATGAAAACGTGAACTCACAACCATTTATGCGTTGGAGAGACCGTTTCTTA
TTTTGTGCTGAAGCAATTTATAAAGCACAAGCTGAAACAGGTGAAATTAAAGGGCATTACTTAAATGCTACTG
CAGGTACATGCGA
